# Adverse event reporting in randomized trials of spinal manipulative therapy: a scoping review of practices and characteristics

**DOI:** 10.1186/s12998-026-00658-8

**Published:** 2026-06-17

**Authors:** Martha Funabashi, Jacob Mangor Normand, Jonas Koch Kristensen, Casper Nim

**Affiliations:** 1 Division of Research and Innovation, Canadian Memorial Chiropractic College, Toronto, Canada; 2 Department of Sports Science and Clinical Biomechanics, University of Southern Denmark, Odense, Denmark; 3 Department of Chiropractic, Université du Québec à Trois-Rivières, Trois-Rivières, Canada; 4 Medical Research Unit, Lillebaelt Hospital, Spine Centre of Southern Denmark, University Hospital of Southern Denmark, Kolding, Denmark; 5 Department of Regional Health Research, University of Southern Denmark, Odense, Denmark

**Keywords:** Adverse events, Randomized controlled trials, Patient safety, Spinal manipulation

## Abstract

**Background:**

Despite the availability of guidelines such as the Consolidated Standards of Reporting Trials (CONSORT) Harms extension, adverse event (AE) reporting in randomized controlled trials (RCTs) of spinal manipulative therapy (SMT) remains inconsistent and incomplete. Many trials fail to provide essential AE details hindering the accurate assessment of SMT-related risks. Mapping the current landscape of AE reporting and identifying deficiencies is critical to improving transparency and advancing patient safety in SMT research and practice.

**Purpose:**

To provide a comprehensive overview of AE reporting in randomized controlled trials (RCTs) investigating high-velocity, low-amplitude SMT for spinal pain, including AE types, characteristics and reporting practices.

**Study design/setting:**

Scoping review based on a previous systematic review.

**Methods:**

We searched PubMed and Epistemonikos for systematic reviews indexed up to February 2022 and conducted an updated systematic search in MEDLINE, EMBASE, Cochrane Central Register of Controlled Trials, Physiotherapy Evidence Database, and Index to Chiropractic Literature from January 1, 2018, to September 12, 2023, with a final update on March 8, 2026. Eligible studies were RCTs involving SMT for spinal pain in adults, including multimodal SMT trials. Studies were included if published in English, Spanish, Portuguese, French, German, Norwegian, Swedish, or Danish. AE data were extracted in three steps: whether AEs were mentioned, collected, and reported. For studies reporting AEs, detailed data on type, severity, duration, onset, relatedness, and additional care were categorized into meaningful subgroups. Summary statistics and visualizations were used to describe reporting patterns.

**Results:**

Of 253 included RCTs, 55% mentioned AEs, 53% collected them, and only 28% reported their occurrence. AE characteristics were inconsistently reported, with wide variation in terminology and classification. Pain-related AEs were most common, typically described as mild in severity. However, characteristics such as duration, onset, and additional care required were often omitted. Relatedness was also inconsistently assessed.

**Conclusion:**

AE reporting in SMT RCTs remains suboptimal and inconsistent with established standards. Improved adherence to standardized reporting frameworks, such as the CONSORT Harms extension, and a cultural shift toward treating AE monitoring as a core component of trial design are urgently needed to enhance transparency and strengthen patient safety.

**Supplementary Information:**

The online version contains supplementary material available at 10.1186/s12998-026-00658-8.

## Introduction

Spinal manipulative therapy (SMT) is a manual therapy intervention that has been increasingly used [1, 2] by several healthcare professions (including physiotherapists, chiropractors, osteopaths, among others) in the management of musculoskeletal conditions, including spinal pain [3, 4]. It consists of the application of a high-velocity, low-amplitude impulse force [5], and is recommended by several clinical practice guidelines and best practice recommendations [3, 4, 6]. Similar to other medical interventions, adverse events (AEs) have been observed following SMT. While serious AEs (e.g., vertebral artery dissection) have been reported following SMT [7], these are rare, typically described in case reports or case series [8], and have not been observed in larger retrospective chart reviews [9]. Most AEs following SMT have been described as mild in severity and transient, lasting up to 48 h, including discomfort/pain, stiffness and headache [10, 11].

Randomized controlled trials (RCTs) are known for being the gold standard study design for measuring effectiveness of interventions as they allow for assessing potential causal relationships between an intervention and its outcomes. From a safety perspective, RCTs are often unable to capture rare and more serious AEs due to the limited sample size and follow-up duration. On the other hand, RCTs also offer a unique opportunity to prospectively identify and record AEs in a controlled setting, providing robust evidence related to both the risks and benefits of an intervention [12]. However, the monitoring and reporting of AEs in RCTs have been greatly discussed to be inconsistent and often insufficient, not only within SMT [13, 14], but also across healthcare disciplines [15–17]. Specifically, RCTs have been observed to frequently underreport AEs, omit definitions, and fail to describe how AEs were monitored, identified, recorded, classified, and analyzed [18, 19]. The CONSORT Harms extension was introduced in 2004 and updated in 2022 aiming to improve the transparency and completeness of harms reporting [12, 20]. Specifically, CONSORT Harms extension provides detailed guidance for transparent reporting of AEs in RCTs, including detailed reporting of AE severity, timing, and relatedness [12, 20]. However, adherence remains low both within SMT and across healthcare disciplines [14, 15, 18].

A growing body of literature highlights that while trial design, efficacy and effectiveness analysis have evolved over the years, the monitoring, collection and reporting of AEs remain rudimentary [19]. Systemic challenges in AE reporting have been emphasized within healthcare disciplines and SMT. These include not only inconsistencies in AE terminology, but also assumptions of safety in non-pharmacological interventions, the tendency to treat AEs as secondary outcomes, and a widespread lack of standardized methods for collecting, classifying, and reporting adverse events [12, 14, 16, 21]. Collectively, these issues contribute to underreporting of AEs and hinder the accurate assessment of SMT-related risks.

Importantly, inadequate AE reporting limits the ability to combine safety data across studies, compromising the true risk-benefit assessment of SMT. By systematically mapping the current landscape of AEs reported in RCTs using SMT, this study aims to better understand the current AE reporting practices within SMT, identify critical gaps, and inform future efforts to enhance the transparency, consistency, and clinical relevance within SMT research. Ultimately, improving AE reporting is fundamental toward advancing patient safety, patient-centered care and ensuring the responsible integration of SMT into clinical practice.

### Aims

This study aimed to provide a comprehensive overview of the AEs reported in RCTs that used SMT. Specifically, we described the AE reporting practices, as well as the AE types and characteristics reported in RCTs that used SMT.

## Methods

This is a scoping review building upon a previously published protocol [22] registered in PROSPERO (CRD42022375836), initially intended for a network meta-analysis on the effectiveness of SMT application procedures [23]. This scoping review is reported according to the Preferred Reporting Items for Systematic Reviews and Meta-Analyses extension for scoping reviews (PRISMA-ScR) [24], PRISMA harms extension [25] (where possible), and follows a pre-registered protocol in Open Science Framework on December 18th, 2024 (https://osf.io/5tn6d/). As this scoping review builds upon an already published systematic review [23], the protocol was registered after the search and data extraction were completed, but before the additional AE data were extracted (see below).

### Eligibility criteria

Any RCT that included an SMT intervention, defined as a high-velocity low-amplitude impulse force, to treat adults (mean age > 16) with spinal pain (in any region) were included. Multimodal SMT trials such as combined SMT and non-thrust spinal mobilization, were also deemed eligible, which is unique to this study and a deviation from the published protocol from the primary network meta-analysis [22]. To be eligible, studies had to be written in English, Spanish, Portuguese, French, German, Norwegian, Swedish, or Danish. Studies not assessing spinal pain or disability or only applying other degrees of mobilization (e.g., Maitland mobilization grade I-IV) were excluded.

### Search strategy and study selection

Originally, a systematic search identified 85 systematic reviews in PubMed and Epistemonikos up to February 25, 2022 that included 442 RCTs, which all were imported into Covidence along with a systematic search in MEDLINE, EMBASE, Cochrane Central Register of Controlled Trials (CENTRAL), Physiotherapy Evidence Database (PEDro), and Index to Chiropractic Literature from January 1, 2018, to September 12, 2023. This search was last updated on March 8th, 2026. The search strategies are available from Supplementary file 1. More details about the search process employed in the primary network meta-analysis (e.g., additional sources for study identification) can be found elsewhere [22, 23].

In the primary network meta-analysis, study selection and initial data extraction were conducted by two independent authors, with CN resolving any conflicts. Most extracted data were detailed in the original protocol [22, 23], with additional data on AE extracted by JKK and JMN under close supervision by MF and CN (see below) specifically for this scoping review.

### Data extraction on adverse events

Adverse event data were extracted in a three-step process. The first step was to assess whether the study “mentioned AEs” or not. Mentioning means that the study noted AEs (i.e., terms related to AE, harms, or similar were written somewhere in the manuscript). Second step was focused on studies that mentioned AEs and assessed if they collected AE data. To assess mentioning and collecting, each study was systematically searched for terms related to AEs in the full text (i.e. “adverse”, “side effect”, “side-effect”, “harm” and “complication”). If those terms were found, the study was skimmed and the method and results section read carefully, which is where AE information should be provided [12]. The third step was to classify each study that collected AEs into whether an AE was reported or not and the following data were extracted:

- Total number of reported AEs.
- Frequency and number of participants who reported AEs (whichever was available). This was further categorized into three distinct reporting strategies:
  - *Event-based reporting*: The tally of the total number of AEs that were reported, which did not indicate how many individuals experienced an event nor if participants experienced more than a single event (e.g., 10 events of pain aggravation occurred).
  - *Proportion reporting*: This approach reported how many participants experienced the type of AE by proportions (e.g., 10% of participants).
  - *Count reporting AEs*: This approach reported how many participants experienced the type of AE by count (e.g., 10 participants).

If the study reported at least one AE, detailed data were extracted:

- Type of AEs – what AE was reported (e.g., pain, dizziness).
- Characteristics of AEs:
  - Severity – how severe was the AE (e.g., mild, moderate, severe).
  - Duration – how long did the AE last (e.g., up to 48 h, between 1 and 7 days).
  - Onset – when AE symptoms started to be experienced (e.g., during intervention, after intervention).
  - Relatedness (or attribution) – process of determining a causal relationship between the reported AE and study intervention (e.g., not related, possibly related).
  - Additional care required – type of healthcare needed to treat the reported AE (e.g., medication, hospitalization).

The detailed AE extraction was done dependent by JKK and JMN due to the complexity of the data and reporting. Data extraction was checked by MF, who also resolved conflicts or flagged issues. All AE data were further classified into whether it was reported at the arm (e.g., SMT or control arm) or study-level.

### Statistical analysis

Summary count statistics were used to characterize the included studies. Pain region, pain duration, age groups, and continent of study conduct were counted for the total sample and by collecting AE or not. Every AE report was registered in tabular form in a spreadsheet in Microsoft Excel (Windows 11). Given the high heterogeneity of how AE events were reported, each AE type and the reported AE characteristic were categorized into meaningful subgroups, which was developed by the research team in consensus while influenced by prior research [10, 26]. Specifically, each AE was categorized based on their type and categorized into 18 different subgroups. Similarly, the *AE characteristics* were further categorized: severity was categorized into 9 subgroups, duration into 12, onset into 10, additional care into 9, and relatedness into 8 subgroups. Supplementary file 2 presents all subgrouping details. Data were illustrated first providing count data on the study-level, then on arm-level (where applicable) and finally on AE-level. Specifically, the following approach was used:

- A flowchart indicating the number of studies mentioning, collecting, and reporting AEs (study-level).
- Temporal distribution of collecting and reporting AEs using a line plot with time binned into 1994 or older and sequential 5-year categories (study-level).
- Bar plot illustrating the number of studies and the strategies used indicating the proportion or count of AEs (arm-level).
- Bubble plot illustrating the number of studies reporting on the 18 AE type subgroups (arm-level and only unique AE types).
- Heat map illustrating the proportion of AE characteristic reported per study (AE event-level).

Of note, if a single study reported two instances of similar AE types (e.g., aggravation of pain); this was only counted and characterized once.

## Results

**Fig. 1:**
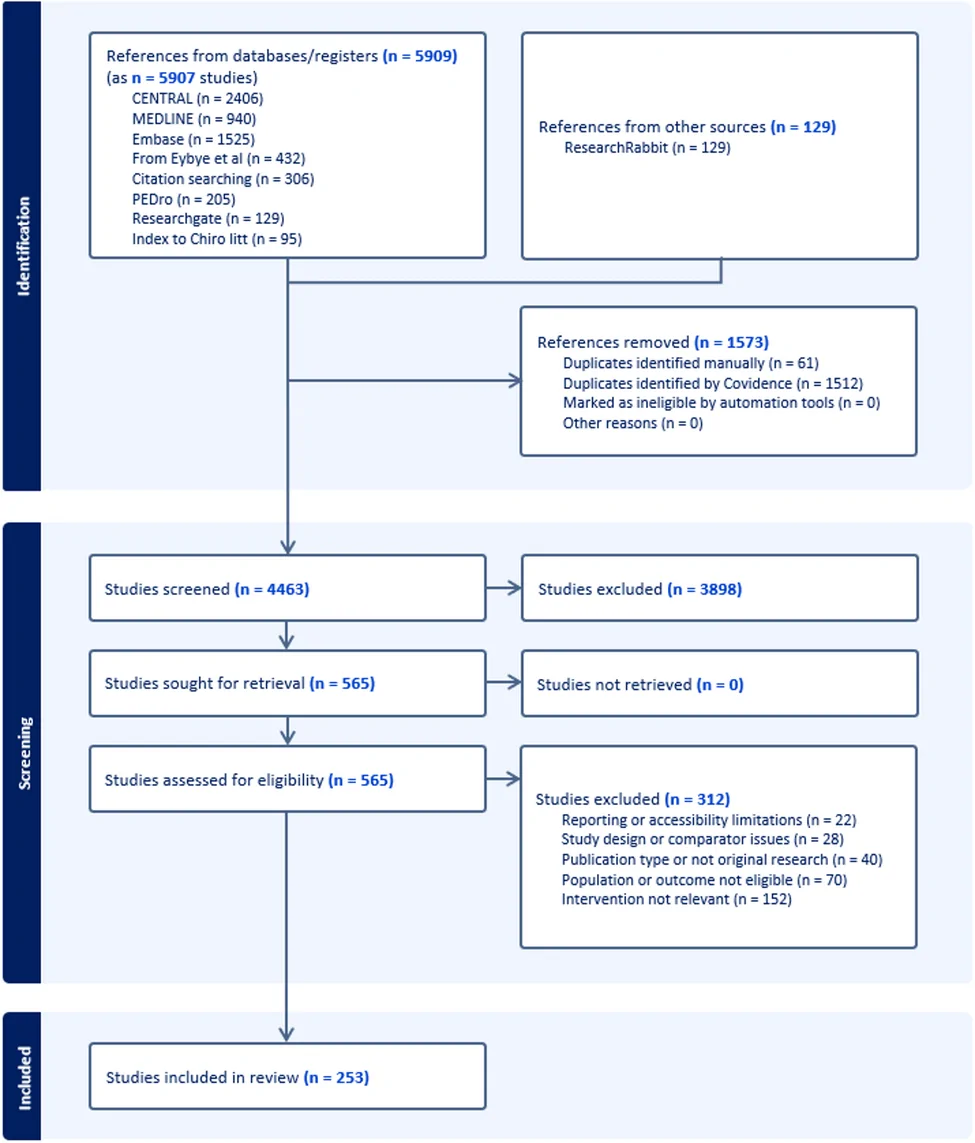
PRISMA flowchart

**Fig. 2:**
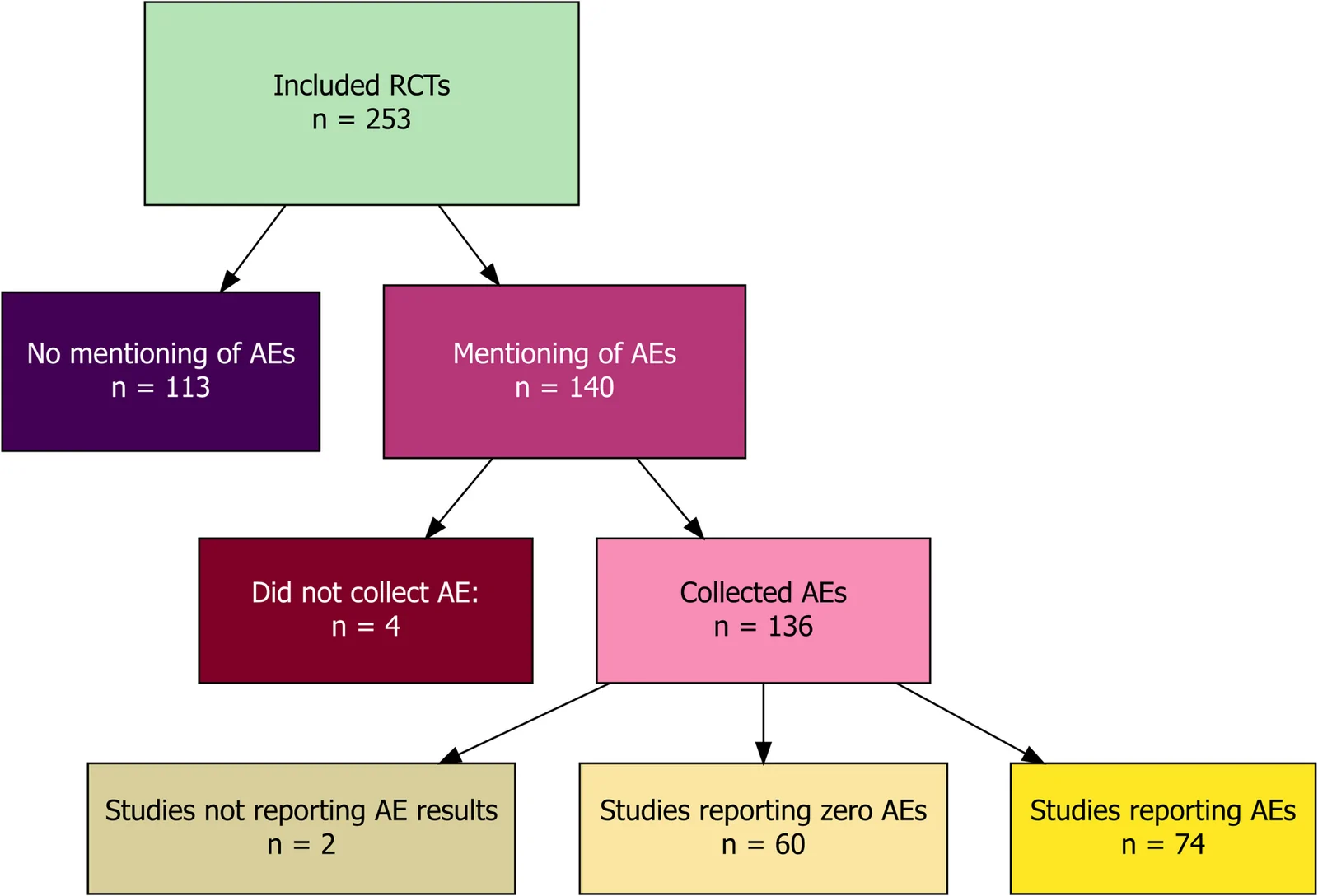
Flowchart illustrating the number of included studies, whether they mentioned, collected and reported adverse events. AE = Adverse event

The original SMT database including the updated search provided 258 SMT trials, where 253 could be included for this analysis (Supplementary file 3). Seven were excluded due to not meeting the language criterion (Fig. 1). Of these, 140 (55%) mentioned AEs in the text and 136 (53%) collected AEs (Fig. 2). When AEs were collected, it was most frequently reported in the Results Sect.  (90%) and few instances in the Discussion (6%) and Methods (4%). The four studies that mentioned AEs but did not collect them, did so in the Discussion (3 studies) or in the Methods Sect.  (1 study).

Studies involving participants with cervical pain with or without headaches, or multiple pain regions collect AEs more often than those focused on low back or thoracic pain. Specifically, 59% of studies on cervical pain with or without headaches and 57% of studies on multiple pain regions collected AEs, compared to 50% of low back pain studies and only 25% of thoracic pain studies. Likewise, studies on older adults (100%) and middle-aged adults (67%) more frequently collected AEs than studies on younger adults (40%). Studies on persistent pain durations also more frequently collected AEs compared to non-persistent durations, 59% versus 47%. Finally, only studies from Oceania, North America, and Europe more frequently collected AEs than not (Table 1). Among the 136 studies that collected AEs, only 74 (54%) of them reported that an AE occurred (Fig. 2).

**Table 1:** , Study characteristics overall and stratified by the mentioning of collecting adverse events

Study characteristics	Overall *N* = 253	Did not collect AEs *N* = 117	Collected AEs *N* = 136
*Region of pain*			
Cervical + headache	110 (100%)	45 (41%)	65 (59%)
Low back pain and pelvic pain	132 (100%)	66 (50%)	66 (50%)
Thoracic pain	4 (100%)	3 (75%)	1 (25%)
Multiple pain regions	7 (100%)	3 (43%)	4 (57%)
*Age groups*			
Young adults (< 40)	123 (100%)	74 (60%)	49 (40%)
Middle age (40–60)	112 (100%)	37 (33%)	75 (67%)
Older age (> 60)	7 (100%)	0 (0%)	7 (100%)
Not able to clarify or not reported	11 (100%)	6 (55%)	5 (45%)
*Duration of pain*			
Non-persistent (< 12 weeks)	53 (100%)	28 (53%)	25 (47%)
Persistent ( > = 12 weeks)	155 (100%)	63 (41%)	92 (59%)
Not able to clarify or not reported	45 (100%)	26 (58%)	19 (42%)
*Geographical region*			
North America	90 (100%)	31 (34%)	59 (66%)
Europe	80 (100%)	39 (49%)	41 (51%)
Asia	38 (100%)	24 (63%)	14 (37%)
South America	21 (100%)	13 (62%)	8 (38%)
Oceania/Australia	16 (100%)	4 (25%)	12 (75%)
Africa	8 (100%)	6 (75%)	2 (25%)

n (%), AE = Adverse event

**Fig. 3:**
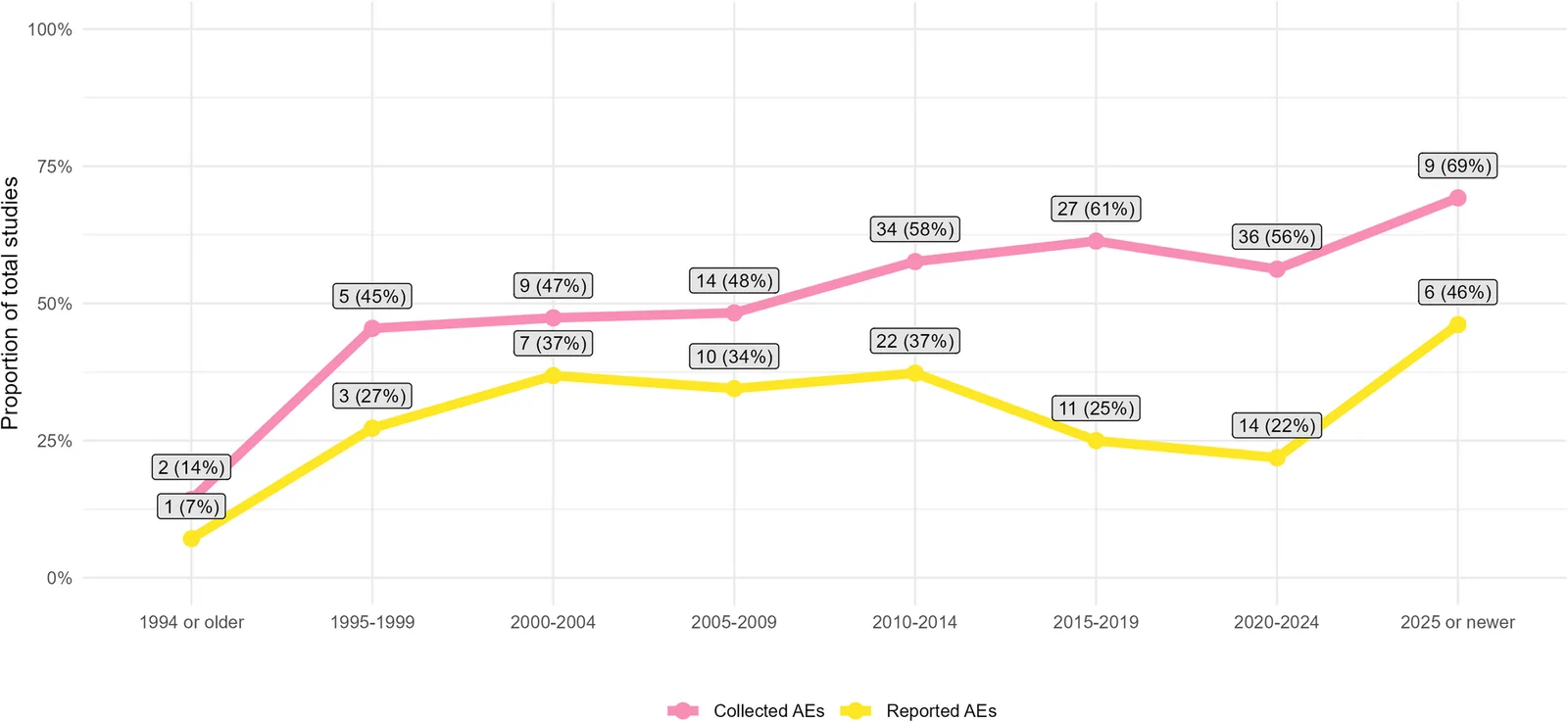
The temporal distribution of studies collecting and reporting adverse events. AE = Adverse event, for Reported AEs, percentages indicate the proportion of studies that collected AEs

When assessing the temporal distribution, the collection of AEs increased substantially from a low of 14% (2 studies) in studies older than 1994, to peaking with 69% (9 studies) recently since 2025. However, the collection of AEs remained stable at 50–60% from 2000 and onwards. Among the studies that collected AEs, the reporting of AE increased slowly until 2014 (65%), decreased to 39% in studies published between 2020 and 2024 and has since increased again recently in 2025 onwards (67%) (Fig. 3).

**Fig. 4:**
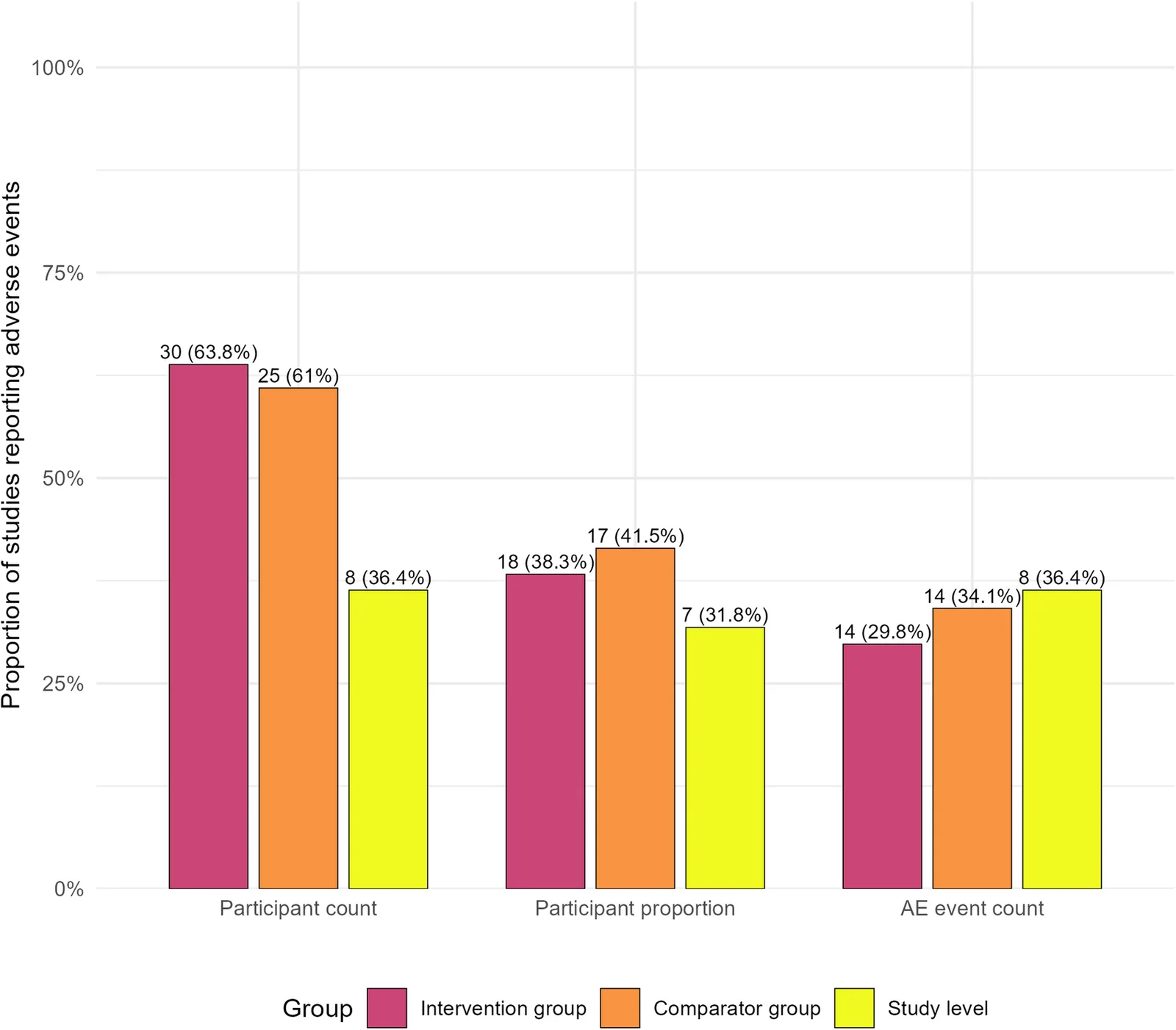
Strategies used to report adverse events. AE = Adverse event. Percentages are indicated from the total tally of 74 studies reporting AEs, and studies could use multiple strategies for reporting AEs

Among the studies that collected AE, 60 (44%) reported zero AEs and 2 (1.5%) did not report any AE results. Among the 74 studies that reported AEs, the most common strategy was to report the count of participants who experienced an AE (63 instances), second was to report the proportion of participants (42 instances), and third was to use the event-based reporting (36 instances). Of note, studies could use multiple strategies for reporting AEs. Reporting AEs on the intervention group-level (the arm including the SMT component) was most common, closely followed by the comparator group (the arm not including SMT), whereas only few studies only provided AEs per study-level (Fig. 4).

**Fig. 5:**
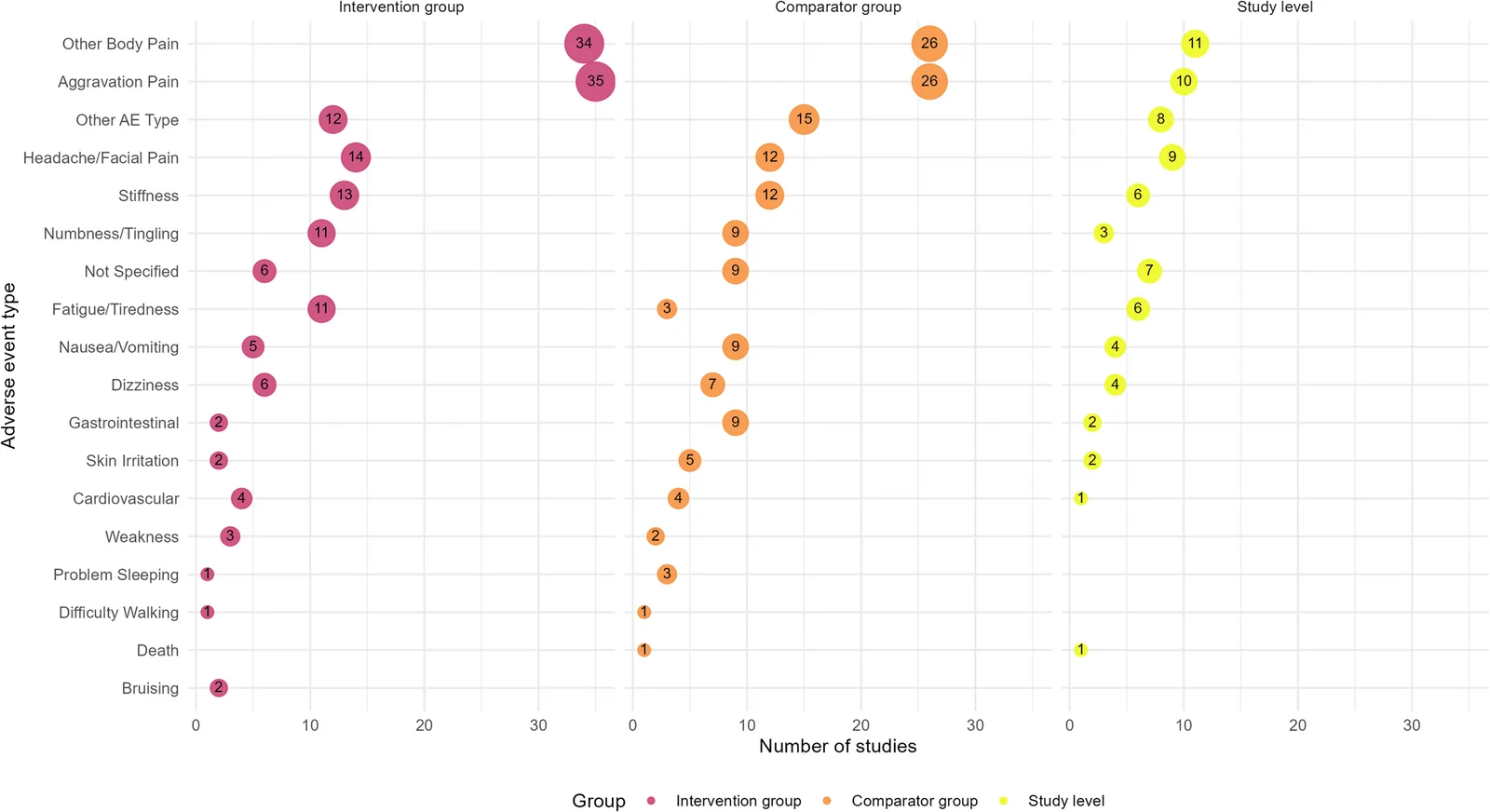
Different types of adverse events reported in 74 studies by intervention group level, comparator group level, and study level. Numbers within bubble indicate the number of studies reporting each adverse event type

At the study level, 16 different types of AEs were reported, and 35 instances reported a different type than the a-priori list created. Furthermore, there were 22 instances where the type could not be classified or was not reported. The most common types of AEs that were reported were related to “pain characteristics” (e.g., other body pain, aggravation of pain, headache/facial pain). Death was reported in two studies, one in the comparator group, and one that was reported on the study-level (Fig. 5).

**Fig. 6:**
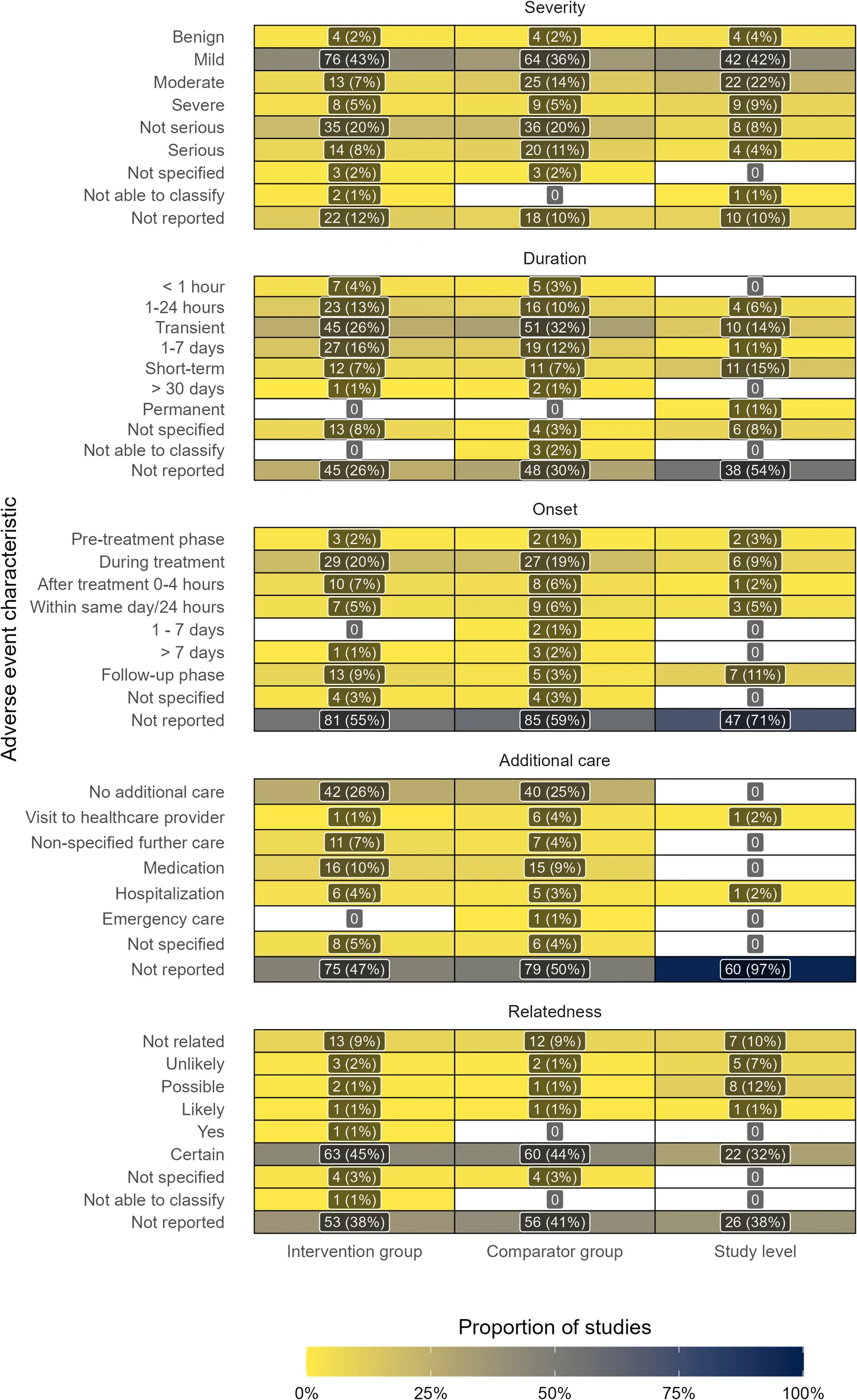
Characteristics of adverse events reported across 74 studies

A total of 389 distinct AE types were reported across groups and study levels. Across all five AE characteristics extracted in this study, most studies only reported the type of AE with severity being the second most common reported AE characteristic. Duration, onset, additional care required and relatedness of AEs were frequently not reported. For severity, the most common AE terminology reported were mild, moderate and not-serious, there were no large differences in the proportion of AE severity that were reported on the intervention, comparator, or the study-level. For duration, transient or within 7 days were most commonly reported and it was slightly more common to report on the intervention/comparator arm than the study-level. The onset of the AE was rarely specified, and when it was, it was either “during the treatment” or “in the follow-up phase,” however, not reporting was by far the most common approach. Again, this was more commonly reported at the intervention/comparator arm rather than the study-level. These results mimicked those of whether additional care was sought; around 25% reported that no additional care was sought, whereas almost 60% did not report any instances of care-seeking. For relatedness, the majority of AEs were classified as either certainly related or not related, whereas the more vague terminology (possible, unlikely) was infrequently used. This was slightly more normal to report at the intervention/comparator arm than the study-level (Fig. 6). The raw extracted data are available from Supplementary file 4 and the data analysis code is available from Supplementary file 5.

## Discussion

This study provides a comprehensive overview of AE reporting practices in RCTs investigating SMT. Our findings show that while over half of the included studies mentioned or collected AEs, only a fraction reported them. When reported, AEs were mostly reported as mild, transient, starting during treatment, and not requiring any additional care. The most commonly reported types of AEs were aggravation of pain and other body pain and were equally reported across study arms. Noteworthy, AEs were reported using distinct approaches (event-based, proportion-based, and count-based), highlighting the lack of standardization in AE reporting practices.

While 55% of included studies mentioned AEs, 23% of included studies reported zero AEs and only 29% of included studies reported the occurrence of AEs. Given that mild, transient symptoms such as post-treatment soreness are commonly observed following SMT [27–30], the complete absence of even mild symptoms raises uncertainty related to the adequacy of AE monitoring. Furthermore, 4 studies actually mentioned AEs, suggesting they were aware, but did not collect any AE data. This suggests that although awareness of AE reporting exists, it may not consistently translate into systematic AE monitoring or transparent reporting. This reflects a weak patient safety culture, where assumptions of safety and superficial acknowledgment of potential AEs may take place over a rigorous commitment to documenting AEs [15, 16, 31, 32]. Although AE collection has improved over time, AE reporting has remained inconsistent, with meaningful improvement apparent only in the most recent publications from 2025 onwards. While the number of trials published since 2025 remains relatively small, this trend only partially aligns with Gorrell et al. (2023), who observed an increase in SMT RCTs reporting of AEs since their previous review in 2016 [13, 14]. This difference can potentially be due to differences in search strategies and eligibility criteria between studies. Regardless of that, findings from both studies (and others) consistently demonstrate that quality of AE reporting in SMT RCTs is generally poor, presenting lack of standardized reporting and is mostly inconsistent with established reporting guidelines and checklists [13, 14, 21].

Trials involving participants with cervical pain with or without headaches, as well as older adults collected AEs more frequently. This likely reflects increased vigilance for potential AEs following cervical SMT [33], particularly given historical concerns about serious AEs reported in the literature and media, or requirements from the ethical boards [34–36]. Similar to our findings, a recent systematic review highlighted substantial limitations in AE reporting, including insufficient detail and considerable heterogeneity across trials, underscoring the need for more rigorous and standardized approaches to AE collection and reporting in SMT research [36]. Based on our findings, future systematic reviews of AE frequency in SMT are unlikely to be informative before more consistent and standardized AE reporting in primary trials is adopted [34, 36]. The current evidence is limited by poor reporting quality and substantial heterogeneity, which significantly limits the confidence in any pooled estimates. Importantly, the variability in AE reporting across studies raises issues related to the credibility and comparability of findings. For example, one study conducted among faculty and students at a chiropractic institution reported 34 AEs from approximately 40 SMT procedures [37], whereas multicenter studies involving more treatment-naïve participants have reported few or no AEs [38–41]. Such differences may reflect variation in adverse event definitions, study populations, and AE monitoring and reporting practices, rather than true differences in underlying risk [36].

The more frequent collection of AEs in studies investigating SMT in older adults is reassuring, considering the increased vulnerability of this population to AEs following SMT [42]. However, the lower rates of AE reporting in studies focused on younger adults and other spinal regions highlight an important area for improvement in AE monitoring and reporting practices. The regional differences in AE collection and reporting should be interpreted cautiously. While language barriers may play a role, the underlying reasons for these differences remain uncertain and may reflect variability in research and safety culture, and reporting norms rather than true differences in AE occurrence.

Among studies that reported AEs, there was great heterogeneity in how AE characteristics (i.e., type, duration, onset, additional care and relatedness) were reported. This highlights that the lack of standardization within the SMT field extends beyond the definition of AEs [21] and it also includes other AE-related terminology and classification such as type categorization and severity grading. The National Cancer Institute’s Common Terminology Criteria for Adverse Events has provided a standardized approach for AE reporting in cancer trials for decades. This includes standardized types and categorization of AEs as well as a severity grading [43]. While the development of standardized AE types and severity grading systems tailored to SMT interventions are currently underway (*manuscripts under review*), this challenge is not unique to SMT; it reflects a broader issue across pain research, where inconsistent terminology and reporting practices continue to hinder the synthesis and interpretation of safety data [15, 16]. Establishing a similar standardized approach within SMT (and across conservative and non-pharmacological interventions more broadly) will significantly improve AE reporting consistency and accuracy, contributing to strengthen the evidence base needed to inform clinical practice and improve patient safety.

Among studies that reported AEs, there was also considerable heterogeneity in how AE frequencies were reported. This reinforces the urgent need for improving the quality of AE reporting in SMT trials. Although, reporting guidelines for AEs exist, such as CONSORT Harms [12], adherence remains low. This gap reflects not only technical and implementation challenges, but also a potential weak patient safety culture. A strong patient safety culture (shared values, attitudes, and behaviors) is fundamental not only for improving the identification and reporting of AEs, but also for ensuring that existing tools and frameworks are valued, appropriately applied, and used [44]. Such a culture also ensures that AEs are not merely acknowledged and then minimized or overlooked, but rather addressed with the necessary seriousness [45]. Beyond the heterogeneity in reporting, it is even more concerning that these AE characteristics were often not reported at all. Researchers should treat AE monitoring and reporting as a core component of trial design rather than a peripheral concern. Journals and peer reviewers also play a vital role in enforcing AE reporting standards and promoting transparency, helping to shift the research culture toward one that fully integrates patient safety into the evidence base, similar to the progress seen with prospective registration [46].

While type and severity were reported, duration, onset and additional care were frequently omitted. When present, the reporting of these AE characteristics was inconsistent and heterogeneous, which is similar to findings from Gorrell et al. (2023). Interestingly, results from Funabashi et al. (2022) indicate that within SMT literature, AE duration, onset and additional care required are often incorporated into AE definitions and used as a component for defining what is an AE and classifying it [21]. This suggests a conceptual inconsistency in how AE characteristics are treated. This disconnect between definition and characteristics may reflect the lack of consensus on what constitutes an AE within SMT that has already been identified and emphasized [42]. The omission of onset and additional care required is concerning, as these characteristics (and others) provide important details for interpreting the impact of AEs and promoting learning and prevention of future AEs [43]. This, once again, reinforces that a standardized definition with clear guidelines for reporting AEs following SMT is urgently needed.

The assessment and reporting of AE relatedness, or attribution to the intervention, is recommended by the CONSORT Harms extension [12]; however, such assessments were inconsistently reported across the included SMT trials. The complexity of assessing AE relatedness has been previously highlighted due to the lack of relevant clinical data at the time of the assessment [44]. Regardless of that, when attribution methods are applied, it is essential that the methods used to determine relatedness are clearly described, along with information on who conducted the assessment [12]. It is also important that all AEs are reported, even those deemed unrelated to the intervention [12]. The lack of transparency in these details has been shown to significantly hinder the comparability of AEs across studies and limit the interpretability of safety data [45].

### Implications for clinical practice and research

The findings of this study have important implications for both clinical practice and future research in SMT. The inconsistent and often incomplete AE reporting precludes accurate assessment of the risk-benefit profile of SMT. For researchers, this study highlights the urgent need to improve the methodological rigor and transparency of AE monitoring and reporting in SMT trials. The lack of standardized definitions, severity classification, and the great variability observed in the details of reported AE characteristics hinder the synthesis of safety data. Adhering to and reinforcing the use of established guidelines (e.g., the CONSORT Harms extension) and contributing to ongoing efforts to develop standardized definitions and guidelines are critical steps forward. Importantly, findings from this study emphasize the need for a cultural shift in how AEs are treated in SMT research. Strengthening patient safety culture, adopting standardized reporting frameworks, and treating AE monitoring as a core component of trial design are essential to advancing both the science and practice of SMT. Additionally, our findings align with the broader context of persistent methodological challenges in SMT research. Specifically, recent evidence [46 ,47] indicate that the overall methodological quality of SMT RCTs has shown limited improvement over time. This indicates that the suboptimal AE reporting is not an isolated issue, but part of a broader pattern underscoring the urgent need for more rigorous trial design, conduct, and reporting.

### Strengths and limitations

A key strength of our study includes the large number of studies included for analysis (*n* = 253) and the detailed mapping of AE characteristics and reporting strategies. By identifying specific gaps, such as the lack of standardized AE terminology and reporting, this study provides actionable directions to guide future research and improve transparency in SMT safety reporting.

Limitations should be acknowledged. First, the classification of AE types and characteristics relied on descriptions available in each study, which were often vague or incomplete and rarely provided clear definitions and may limit the accuracy of categorization. Second, this study used a large database of RCTs investigating SMT for spinal pain and studies using other methodologies or investigating other conditions were not included. This is particularly important as mild AEs following SMT are relatively common, whereas serious or catastrophic AEs are rare. As a result, RCTs (particularly those with modest sample sizes and limited follow‑up) are generally not well suited to detect rare serious AEs. Consequently, alternative research designs, such as large prospective observational studies or registry‑based surveillance, are necessary to adequately capture serious AEs. The absence of serious AEs in this review should therefore not be interpreted as evidence that such events do not occur. This study focused on trials using high-velocity, low-amplitude SMT. However, some studies did not clearly define if mobilization was used in addition to SMT. Therefore, it is possible that our results include AEs that might have occurred following mobilizations. However, it is unlikely that substantial differences would have been found if all trials using mobilizations were excluded as there is very limited evidence related to AEs following mobilization. Finally, included studies were also limited to studies reported in specific languages (English, Spanish, Portuguese, French, German, Norwegian, Swedish, and Danish), and may have missed potentially relevant studies reported in other languages.

## Conclusion

This study highlights significant inconsistencies and gaps in the reporting of AEs in RCTs investigating SMT. Although awareness and collection of AEs have increased over time, progress in AE reporting has shown less consistent improvements. Significant gaps remain in the consistency, completeness, and transparency of how AEs are defined, collected, and reported. Key characteristics such as onset, duration, relatedness, and additional care required are frequently omitted or inconsistently reported, limiting the clinical utility of safety data. Findings from this study highlight the urgent need for standardized AE reporting practices and greater adherence to established guidelines, such as the CONSORT Harms extension, and a cultural shift toward treating AEs as essential outcomes are critical steps toward improving transparency and accountability in SMT research. Given the substantial heterogeneity and inconsistent AE reporting, efforts may be better directed toward improving primary study reporting before undertaking further systematic reviews of AEs in SMT trials.

## Supplementary Information

Below is the link to the electronic supplementary material.

Supplementary Material 1: Search strategies

Supplementary Material 2: Overview of adverse event type and characteristic category subgroups

Supplementary Material 3: List of included and excluded studies

Supplementary Material 4: Extracted raw data

Supplementary Material 5: Data analysis code

## Data Availability

Data used for the analysis are available in Supplementary File 4.

## Abbreviations

AE: Adverse event
CONSORT: Consolidated Standards of Reporting Trials
PRISMA: ScR–Preferred Reporting Items for Systematic Reviews and Meta–Analyses extension for scoping reviews
RCT: Randomized controlled trials
SMT: Spinal manipulative therapy
