# Supplementary material for: Adverse event reporting in randomized trials of spinal manipulative therapy: a scoping review of practices and characteristics
Source: Chiropr Man Therap. 2026 Jun 17;34:37. doi: 10.1186/s12998-026-00658-8 (PMC13488154; doi:10.1186/s12998-026-00658-8)
Supplement: Supplementary file 1 [file 12998_2026_658_MOESM1_ESM.docx]

**The search strategy across databases.**

## MEDLINE (via Ovid)

1 randomized controlled trial.pt.

2 controlled clinical trial.pt.

3 randomized.ab.

4 placebo.ab.

5 randomly.ab.

6 trial.ab.

7 groups.ab.

8 1 or 2 or 3 or 4 or 5 or 6 or 7

9 back pain.tw.

10 exp low back pain/

11 backache.tw.

12 lumbago.tw. (

13 dorsalgia.tw.

14 lumbar vertebra* pain.mp.

15 cervical vertebra* pain.mp

16 thoracic vertebra* pain.mp

17 thoracic* pain.mp

18 chest pain.mp

19 neck pain.tw.

20 spin* diseas*.mp

21 spin* pain.mp

22 thoracic pain.tw

23 9 or 10 or 11 or 12 or 13 or 14 or 15 or 16 or 17 or 18 or 19 or 20 or 21 or 22

24 manipulat*.tw.

25 exp Musculoskeletal Manipulations/

26 high velocity low amplitude.tw.

27 high velocity thrust.tw.

28 ((spinal or joint) adj3 adjust*).tw.

29 mobiliz*.tw.

30 mobilis*.tw.

31 chiroprac*

32 osteopath*

33 exp musculoskeletal manipulations/

34 24 or 25 or 26 or 27 or 28 or 29 or 30 or 31 or 32 or 33

35 8 and 23 and 34

## EMBASE (via Ovid)

1 crossover-procedure/

2 double-blind procedure/

3 randomized controlled trial/

4 single-blind procedure/

5 (random* or factorial* or crossover* or cross over* or placebo* or (doubl* adj blind) or (singl adj blind) or assign or allocat* or volunteer*).mp.

6 1 or 2 or 3 or 4 or 5

7 back pain.tw.

8 exp low back pain/

9 backache.tw.

10 lumbago.tw.

11 dorsalgia.tw.

12 lumbar vertebra* pain.mp

13 cervical vertebra* pain.mp

14 thoracic vertebra* pain.mp

15 thoracic* pain.mp

16 chest pain.mp

17 neck pain.tw.

18 spin* diseas*.mp

19 spin* pain.mp

20 thoracic pain.tw

21 7 or 8 or 9 or 10 or 11 or 12 or 13 or 14 or 15 or 16 or 17 or 18 or 19 or 20

22 manipulat*.tw.

23 exp musculoskeletal manipulation/

24 high velocity low amplitude.tw.

25 high velocity thrust.tw.

26 ((spinal or joint) adj3 adjust*).tw.

27 mobiliz*.tw.

28 exp mobilization/

29 exp joint mobilization/

30 chiroprac*

31 osteopath*

32 22 or 23 or 24 or 25 or 26 or 27 or 28 or 29 or 30 or 31

33 5 and 20 and 32

#### Cochrane Library,

1 (back pain):ti,ab,kw

2 MeSH descriptor: [Low Back Pain] explode all trees

3 MeSH descriptor: [Neck Pain] explode all trees

4 MeSH descriptor: [Spine] explode all trees

5 (backache):ti,ab,kw

6 (lumbago):ti,ab,kw

7 (dorsalgia):ti,ab,kw

8 ((lumb* near/3 pain)):ti,ab,kw

9 ((cerv* near/3 pain)):ti,ab,kw

10 #1 OR #2 OR #3 OR #4 OR #5 OR #6 OR #7 OR #8 OR #9

11 (manipulat*):ti,ab,kw

12 MeSH descriptor: [Manipulation, Spinal] explode all trees

13 (high velocity low amplitude):ti,ab,kw

14 (high velocity thrust):ti,ab,kw

15 (((spinal or joint) near/3 adjust*)):ti,ab,kw

16 (mobiliz*):ti,ab,kw

17 (mobilis*):ti,ab,kw

18 MeSH descriptor: [Musculoskeletal Manipulations] explode all trees

19 #11 OR #12 OR #13 OR #14 OR #15 OR #16 OR #17 OR #18

20 #10 AND #19

## PEDro

1 Abstract & Title: manipulat* AND Body Part: lumbar spine, sacro-iliac joint or pelvis AND Method: clinical trial

2 Abstract & Title: manipulat* AND Body Part: head or neck AND Method: clinical trial

3 Abstract & Title: manipulat* AND Body Part: thoracic spine AND Method: clinical trial

## Index to Chiropractic Literature

1 Publication Type:Clinical Trial

2 Publication Type:Controlled Clinical Trial

3 Publication Type:Randomized Controlled Trial

4 All Fields:random* OR All Fields:placebo* OR All Fields:sham*

5 All Fields:clinical trial

6 All Fields:controlled trial

7 All Fields:double blind

8 All Fields:double-blind

9 All Fields:single blind

10 All Fields:single-blind

11 Publication Type:Clinical Trial OR , Publication Type:Controlled Clinical Trial OR , Publication Type:Randomized Controlled Trial OR All Fields:random* OR All Fields:placebo* OR All Fields:sham* OR All Fields:clinical trial OR All Fields:controlled trial OR All Fields:double blind OR All Fields:double-blind OR All Fields:single blind OR All Fields:single-blind

12 Article Title:“back pain” OR Abstract/Notes:“back pain”

13 Article Title:“neck pain” OR Abstract/Notes:“neck pain”

14 Subject:“Low Back Pain”

15 Subject:“Neck Pain”

16 All Fields:thoracic pain

17 Article Title:backache OR Abstract/Notes:backache

18 Article Title:lumbago OR Abstract/Notes:lumbago

19 Article Title:dorsalgia OR Abstract/Notes:dorsalgia

20 Article Title:“lumbar pain” OR Abstract/Notes:“lumbar pain”

21 Article Title:“lumbosacral pain” OR Abstract/Notes:“lumbosacral pain”

22 All Fields:cervicothoracic pain

23 Article Title:“back pain” OR Abstract/Notes:“back pain” OR Article Title:“neck pain” OR Abstract/Notes:“neck pain” OR Subject:“Low Back Pain” OR Subject:“Neck Pain” OR All Fields:thoracic pain OR Article Title:backache OR Abstract/Notes:backache OR Article Title:lumbago OR Abstract/Notes:lumbago OR Article Title:dorsalgia OR Abstract/Notes:dorsalgia OR Article Title:“lumbar pain” OR Abstract/Notes:“lumbar pain” OR Article Title:“lumbosacral pain” OR Abstract/Notes:“lumbosacral pain” OR All Fields:cervicothoracic pain

24 Article Title:manipulat* OR Abstract/Notes:manipulat*

25 Subject:“Manipulation, Chiropractic”

26 Subject:“Manipulation, Orthopedic”

27 Subject:“Manipulation, Osteopathic”

28 Subject:“Manipulation, Joint”

29 Subject:“Musculoskeletal Manipulations”

30 Article Title:“high velocity low amplitude” OR Abstract/Notes:“high velocity low amplitude”

31 Article Title:“high velocity thrust” OR Abstract/Notes:“high velocity thrust”

32 Article Title:“spinal adjustment” OR Abstract/Notes:“spinal adjustment”

33 Article Title:“joint adjustment” OR Abstract/Notes:“joint adjustment”

34 Article Title:mobiliz* OR Abstract/Notes:mobiliz*

35 Article Title:mobilis* OR Abstract/Notes:mobilis*

36 Subject:“Mobilization”

37 Subject:“Mobilization, Joint”

38 All Fields: gonstead

39 Article Title:manipulat* OR Abstract/Notes:manipulat* OR Subject:“Manipulation, Chiropractic” OR Subject:“Manipulation, Orthopedic” OR Subject:“Manipulation, Osteopathic” OR Subject:“Manipulation, Joint” OR Subject:“Musculoskeletal Manipulations” OR Article Title:“high velocity low amplitude” OR Abstract/Notes:“high velocity low amplitude” OR Article Title:“high velocity thrust” OR Abstract/Notes:“high velocity thrust” OR Article Title:“spinal adjustment” OR Abstract/Notes:“spinal adjustment” OR Article Title:“joint adjustment” OR Abstract/Notes:“joint adjustment” OR Article Title:mobiliz* OR Abstract/Notes:mobiliz* OR Article Title:mobilis* OR Abstract/Notes:mobilis* OR Subject:“Mobilization” OR Subject:“Mobilization, Joint” OR All Fields: gonstead

40 Article Title:pain OR Abstract/Notes:pain

40 Subject:“Pain”

42 Article Title:pain OR Abstract/Notes:pain OR Subject:“Pain”

43 Publication Type:Clinical Trial OR , Publication Type:Controlled Clinical Trial OR , Publication Type:Randomized Controlled Trial OR All Fields:random* OR All Fields:placebo* OR All Fields:sham* OR All Fields:clinical trial OR All Fields:controlled trial OR All Fields:double blind OR All Fields:double-blind OR All Fields:single blind OR All Fields:single-blind AND Article Title:“back pain” OR Abstract/Notes:“back pain” OR Article Title:“neck pain” OR Abstract/Notes:“neck pain” OR Subject:“Low Back Pain” OR Subject:“Neck Pain” OR All Fields:thoracic pain OR Article Title:backache OR Abstract/Notes:backache OR Article Title:lumbago OR Abstract/Notes:lumbago OR Article Title:dorsalgia OR Abstract/Notes:dorsalgia OR Article Title:“lumbar pain” OR Abstract/Notes:“lumbar pain” OR Article Title:“lumbosacral pain” OR Abstract/Notes:“lumbosacral pain” OR All Fields:cervicothoracic pain AND Article Title:pain OR Abstract/Notes:pain
