# Supplementary material for: Adverse event reporting in randomized trials of spinal manipulative therapy: a scoping review of practices and characteristics
Source: Chiropr Man Therap. 2026 Jun 17;34:37. doi: 10.1186/s12998-026-00658-8 (PMC13488154; doi:10.1186/s12998-026-00658-8)
Supplement: Supplementary file 2 [file 12998_2026_658_MOESM3_ESM.docx]

**Included Studies**

| Title | Authors | Year |
| --- | --- | --- |
| The effects of thrust joint manipulation on the resting and contraction thickness of transversus abdominis in patients with low back pain: A randomized control trial. | Fosberg KK; Puentedura E; Schmitz B; Jain TK; Cleland JA | 2020 |
| Immediate effects of cervicothoracic junction mobilization versus thoracic manipulation on the range of motion and pain in mechanical neck pain with cervicothoracic junction dysfunction: A pilot randomized controlled trial. | Joshi S; Balthillaya G; Neelapala YVR | 2020 |
| Immediate effects of a lumbar spine manipulation on pain sensitivity and postural control in individuals with nonspecific low back pain: A randomized controlled trial. | Loss JF; da Silva LDS; Miranda LF; Groisman S; Wagner Neto ES; Souza C; Candotti CT; Affiliation(G) / Brazil / Bahia / Salvador | 2020 |
| Spinal manipulative therapy and exercise for older adults with chronic low back pain: A randomized clinical trial . | Schulz C; Evans R; Maiers M; Schulz K; Leininger B; Bronfort G | 2019 |
| Impact of spinal manipulation on lower extremity motor control in lumbar spinal stenosis patients: A small-scale assessor-blind randomized clinical trial. | Passmore SR; Johnson MG; Aloraini SM; Cooper S; Glazebrook CM | 2019 |
| Changes in cervicocephalic kinesthetic sensibility, widespread pressure pain sensitivity, and neck pain after cervical thrust manipulation in patients with chronic mechanical neck pain: A randomized clinical trial. | García-Pérez-Juana D; Fernández-de-las-Peñas C; Arias-Buria JL; Cleland JA; Plaza-Manzano G; Ortega-Santiago R | 2018 |
| Immediate influence of lumbar spine manipulation on pain, functional reach, static balance, and walking gait kinematics of individuals with acute low back pain. | Ward J; Tyer K; Pourmoghaddam A | 2018 |
| Chiropractic treatment including instrument-assisted manipulation for non-specific dizziness and neck pain in community-dwelling older people: A feasibility randomised sham-controlled trial [randomized controlled trial]. | Kendall JC; French SD; Hartvigsen J; Azari MF | 2018 |
| Immediate Effects of Region-Specific and Non–Region-Specific Spinal Manipulative Therapy in Patients With Chronic Low Back Pain: A Randomized Controlled Trial | de Oliveira, Ronaldo Fernando; Liebano, Richard Eloin; Costa, Lucíola da Cunha Menezes; Rissato, Lívia Leticia; Costa, Leonardo Oliveira Pena | 2013 |
| Efficacy of Cervical Endplay Assessment as an Indicator for Spinal Manipulation: | Haas, Mitchell; Groupp, Elyse; Panzer, David; Partna, Lester; Lumsden, Steve; Aickin, Mikel | 2003 |
| The effect of direction specific thoracic spine manipulation on the cervical spine: a randomized controlled trial | Karas, Steve; Olson Hunt, Megan J.; Temes, Bill; Thiel, Martin; Swoverland, Trenton; Windsor, Brett | 2018 |
| Immediate Changes in Widespread Pressure Pain Sensitivity, Neck Pain, and Cervical Range of Motion After Cervical or Thoracic Thrust Manipulation in Patients With Bilateral Chronic Mechanical Neck Pain: A Randomized Clinical Trial | Martínez-Segura, Raquel; de-la-Llave-Rincón, Ana Isabel; Ortega-Santiago, Ricardo; Cleland, Joshua A.; Fernández-de-las-Peñas, César | 2012 |
| Short-term effects of spinal thrust joint manipulation on postural sway in patients with chronic mechanical neck pain: a randomized controlled trial | Romero del Rey, Raúl; Saavedra Hernández, Manuel; Rodríguez Blanco, Cleofás; Palomeque del Cerro, Luis; Alarcón Rodríguez, Raquel | 2020 |
| A randomized clinical trial to compare the immediate effects of seated thoracic manipulation and targeted supine thoracic manipulation on cervical spine flexion range of motion and pain | Karas, Steve; Olson Hunt, Megan J | 2014 |
| [Chronic low back pain and vertebral manipulation] | Ghroubi, S.; Elleuch, H.; Baklouti, S.; Elleuch, M.-H. | 2007 |
| Clinical effects of short and long lever spinal thrust manipulation in non-specific chronic low back pain: A biomechanical perspective | Nambi, S. Gopal; Inbasekaran, Dipika; Khuman, Ratan; Devi, Surbala; Satani, Kalpesh | 2013 |
| The immediate effect of activator vs. meric adjustment on acute low back pain: a randomised controlled trial | Gemmell HA; Jacobson BH | 1995 |
| Examination of a Clinical Prediction Rule to Identify Patients With Neck Pain Likely to Benefit From Thoracic Spine Thrust Manipulation and a General Cervical Range of Motion Exercise: Multi-Center Randomized Clinical Trial | Cleland, Joshua A.; Mintken, Paul E.; Carpenter, Kristin; Fritz, Julie M.; Glynn, Paul; Whitman, Julie; Childs, John D. | 2010 |
| Upper Cervical and Upper Thoracic Thrust Manipulation Versus Nonthrust Mobilization in Patients With Mechanical Neck Pain: A Multicenter Randomized Clinical Trial | Dunning, James R.; Cleland, Joshua A.; Waldrop, Mark A.; Arnot, Cathy; Young, Ian; Turner, Michael; Sigurdsson, Gisli | 2012 |
| Short-term effects of manipulative treatment versus a therapeutic home exercise protocol for chronic cervical pain: A randomized clinical trial | Galindez-Ibarbengoetxea, Xabier; Setuain, Igor; Ramírez-Velez, Robinson; Andersen, Lars L.; González-Izal, Miriam; Jauregi, Andoni; Izquierdo, Mikel | 2018 |
| Relative effectiveness and adverse effects of cervical manipulation, mobilisation and the activator instrument in patients with sub-acute non-specific neck pain: results from a stopped randomised trial | Gemmell, Hugh; Miller, Peter | 2010 |
| Manual and Instrument Applied Cervical Manipulation for Mechanical Neck Pain: A Randomized Controlled Trial | Lindsay M. Gorrell; Beath, Kenneth; Roger M. Engel | 2016 |
| Pragmatically Applied Cervical and Thoracic Nonthrust Manipulation Versus Thrust Manipulation for Patients With Mechanical Neck Pain: A Multicenter Randomized Clinical Trial | Griswold, David; Learman, Ken; Kolber, Morey J.; O'Halloran, Bryan; Cleland, Joshua A. | 2018 |
| Is one better than another?: A randomized clinical trial of manual therapy for patients with chronic neck pain | Izquierdo Pérez, Honorio; Alonso Perez, Jose Luis; Gil Martinez, Alfonso; La Touche, Roy; Lerma-Lara, Sergio; Commeaux Gonzalez, Noelia; Arribas Perez, Hector; Bishop, Mark D.; Fernández-Carnero, Josue | 2014 |
| The effectiveness of thoracic manipulation on patients with chronic mechanical neck pain – A randomized controlled trial | Lau, Herman Mun Cheung; Wing Chiu, Thomas Tai; Lam, Tai-Hing | 2011 |
| Short-Term Effects of Kinesio Taping Versus Cervical Thrust Manipulation in Patients With Mechanical Neck Pain: A Randomized Clinical Trial | Saavedra-Hernández, Manuel; Castro-Sánchez, Adelaida M.; Arroyo-Morales, Manuel; Cleland, Joshua A.; Lara-Palomo, Inmaculada C.; Fernández-de-las-Peñas, César | 2012 |
| Immediate Changes in Neck Pain Intensity and Widespread Pressure Pain Sensitivity in Patients With Bilateral Chronic Mechanical Neck Pain: A Randomized Controlled Trial of Thoracic Thrust Manipulation vs Non–Thrust Mobilization | Salom-Moreno, Jaime; Ortega-Santiago, Ricardo; Cleland, Joshua Aland; Palacios-Ceña, Maria; Truyols-Domínguez, Sebastian; Fernández-de-las-Peñas, César | 2014 |
| The Effects of Thoracic Manipulation Versus Mobilization for Chronic Neck Pain: a Randomized Controlled Trial Pilot Study | Suvarnnato, Thavatchai; Puntumetakul, Rungthip; Kaber, David; Boucaut, Rose; Boonphakob, Yodchai; Arayawichanon, Preeda; Chatchawan, Uraiwan | 2013 |
| Effect of spinal manipulative treatment on cardiovascular autonomic control in patients with acute low back pain | Younes, Mohamed; Nowakowski, Karine; Didier-Laurent, Benoit; Gombert, Michel; Cottin, François | 2017 |
| Regional manual therapy and motor control exercise for chronic low back pain: a randomized clinical trial | Zafereo, Jason; Wang-Price, Sharon; Roddey, Toni; Brizzolara, Kelli | 2018 |
| Does Maintained Spinal Manipulation Therapy for Chronic Nonspecific Low Back Pain Result in Better Long-Term Outcome? | Senna, Mohammed K.; Machaly, Shereen A. | 2011 |
| Comparison between high-velocity low-amplitude manipulation and muscle energy technique on pain and trunk neuromuscular postural control in male workers with chronic low back pain: A randomised crossover trial | Sturion, Leandro A.; Nowotny, Alexandre H.; Barillec, Fabrice; Barette, Gilles; Santos, Gabriela K.; Teixeira, Fellipe A.; Fernandes, Karen P.; Silva, Rubens da | 2020 |
| Effects of Thoracic Mobilization and Manipulation on Function and Mental State in Chronic Lower Back Pain | Sung, Youn-Bum; Lee, Jung-Ho; Park, Young-Han | 2014 |
| Comparison of Short-Term Response to Two Spinal Manipulation Techniques for Patients With Low Back Pain in a Military Beneficiary Population | Sutlive, Thomas G.; Mabry, Lance M.; Easterling, Emmanuel J.; Durbin, Jose D.; Hanson, Stephen L.; Wainner, Robert S.; Childs, John D. | 2009 |
| Evaluation of functional disability and pain in patients with chronic low back pain submitted to physiotherapy | Teixeira, Andrei Luiz Sales; Silva, Noberto Fernandes da; Filho, Edson Meneses da Silva | 2016 |
| Effect of Spinal Manipulative and Mobilization Therapies in Young Adults With Mild to Moderate Chronic Low Back Pain: A Randomized Clinical Trial | Thomas, James S.; Clark, Brian C.; Russ, David W.; France, Christopher R.; Ploutz-Snyder, Robert; Corcos, Daniel M.; for the RELIEF Study Investigators | 2020 |
| United Kingdom back pain exercise and manipulation (UK BEAM) randomised trial: effectiveness of physical treatments for back pain in primary care | Team, UK BEAM Trial | 2004 |
| Short-Term Effect of Spinal Manipulation on Pain Perception, Spinal Mobility, and Full Height Recovery in Male Subjects With Degenerative Disk Disease: A Randomized Controlled Trial | Vieira-Pellenz, Felipe; Oliva-Pascual-Vaca, Ángel; Rodriguez-Blanco, Cleofás; Heredia-Rizo, Alberto Marcos; Ricard, François; Almazán-Campos, Ginés | 2014 |
| Effects of Chiropractic Care on Strength, Balance, and Endurance in Active-Duty U.S. Military Personnel with Low Back Pain: A Randomized Controlled Trial | Vining, Robert; Long, Cynthia R.; Minkalis, Amy; Gudavalli, M. Ram; Xia, Ting; Walter, Joan; Coulter, Ian; Goertz, Christine M. | 2020 |
| Osteopathic manipulative treatment in obese patients with chronic low back pain: A pilot study | Vismara, Luca; Cimolin, Veronica; Menegoni, Francesco; Zaina, Fabio; Galli, Manuela; Negrini, Stefano; Villa, Valentina; Capodaglio, Paolo | 2012 |
| Spinal High-Velocity Low Amplitude Manipulation in Acute Nonspecific Low Back Pain: A Double-Blinded Randomized Controlled Trial in Comparison With Diclofenac and Placebo | von Heymann, Wolfgang J.; Schloemer, Patrick; Timm, Juergen; Muehlbauer, Bernd | 2013 |
| The McKenzie Method Compared With Manipulation When Used Adjunctive to Information and Advice in Low Back Pain Patients Presenting With Centralization or Peripheralization: A Randomized Controlled Trial | Petersen, Tom; Larsen, Kristian; Nordsteen, Jan; Olsen, Steen; Fournier, Gilles; Jacobsen, Soren | 2011 |
| A Clinical Prediction Rule to Identify Patients With Low Back Pain Who Are Likely to Experience Short-Term Success Following Lumbar Stabilization Exercises: A Randomized Controlled Validation Study | Rabin, Alon; Shashua, Anat; Pizem, Koby; Dickstein, Ruthy; Dar, Gali | 2014 |
| Manipulation does not add to the effect of extension exercises in chronic low-back pain (LBP). A randomized, controlled, double blind study | Rasmussen, Jens; Lætgaard, Jes; Lindecrona, Anne-Louise; Qvistgaard, Etienne; Bliddal, Henning | 2008 |
| Comparison of physical treatments versus a brief pain-management programme for back pain in primary care: a randomised clinical trial in physiotherapy practice | Hay, EM; Mullis, R; Lewis, M; Vohora, K; Main, CJ; Watson, P; Dziedzic, KS; Sim, J; Lowe, C Minns; Croft, PR | 2005 |
| A Randomized Clinical Trial Comparing Chiropractic Adjustments to Muscle Relaxants for Subacute Low Back Pain | Hoiriis, Kathryn T.; Pfleger, Bruce; McDuffie, Frederic C.; Cotsonis, George; Elsangak, Omar; Hinson, Roger; Verzosa, Gregoria T. | 2004 |
| A Randomized Clinical Trial of Manipulative Therapy and Interferential Therapy for Acute Low Back Pain | Hurley, Deirdre A.; McDonough, Suzanne M.; Dempster, Martin; Moore, Ann P.; Baxter, G. David | 2004 |
| The Effectiveness of Thai Massage and Joint Mobilization | Chantip Juntakarn, M. A.; Thavat Prasartritha, M. D.; Prapoj Petrakard, M. D. | 2017 |
| A randomised controlled trial of spinal manipulative therapy in acute low back pain | Jüni, P.; Battaglia, M.; Nüesch, E.; Hämmerle, G.; Eser, P.; Beers, R. van; Vils, D.; Bernhard, J.; Ziswiler, H.-R.; Dähler, M.; Reichenbach, S.; Villiger, P. M. | 2009 |
| Osteopathic Manual Treatment and Ultrasound Therapy for Chronic Low Back Pain: A Randomized Controlled Trial | Licciardone, John C.; Minotti, Dennis E.; Gatchel, Robert J.; Kearns, Cathleen M.; Singh, Karan P. | 2013 |
| Osteopathic Manipulative Treatment for Chronic Low Back Pain: A Randomized Controlled Trial | Licciardone, John C.; Stoll, Scott T.; Fulda, Kimberly G.; Russo, David P.; Siu, Jeff; Winn, William; Swift, Jon Jr | 2003 |
| Comparative effectiveness of Chuna manual therapy versus conventional usual care for non-acute low back pain: a pilot randomized controlled trial | Lim, Kyeong-Tae; Hwang, Eui-Hyoung; Cho, Jae-Heung; Jung, Jae-Young; Kim, Koh-Woon; Ha, In-Hyuk; Kim, Me-riong; Nam, Kibong; Lee A, Min ho; Lee, Jun-Hwan; Kim, Namkwen; Shin, Byung-Cheul | 2019 |
| Comparing targeted thrust manipulation with general thrust manipulation in patients with low back pain. A general approach is as effective as a specific one. A randomised controlled trial | McCarthy, Christopher J.; Potter, Louise; Oldham, Jackie A. | 2019 |
| A prospective randomised controlled trial of spinal manipulation and ultrasound in the treatment of chronic low back pain | Mohseni-Bandpei, Mohammad A.; Critchley, Jacqueline; Staunton, Thomas; Richardson, Barbara | 2006 |
| The effect on clinical outcomes when targeting spinal manipulation at stiffness or pain sensitivity: a randomized trial | Nim, Casper Glissmann; Kawchuk, Gregory Neil; Schiøttz-Christensen, Berit; O’Neill, Søren | 2020 |
| Short-term Effects of Thoracic Spine Thrust Manipulation, Exercise, and Education in Individuals With Low Back Pain: A Randomized Controlled Trial | Fisher, Laura R.; Alvar, Brent A.; Maher, Sara F.; Cleland, Joshua A. | 2020 |
| Adding Chiropractic Manipulative Therapy to Standard Medical Care for Patients With Acute Low Back Pain: Results of a Pragmatic Randomized Comparative Effectiveness Study | Goertz, Christine M.; Long, Cynthia R.; Hondras, Maria A.; Petri, Richard; Delgado, Roxana; Lawrence, Dana J.; Owens, Edward F.; Meeker, William C. | 2013 |
| A Randomized Controlled Clinical Trial of Stay-Active Care Versus Manual Therapy in Addition to Stay-Active Care: Functional Variables and Pain | Grunnesjö, Marie I.; Bogefeldt, Johan P.; Svärdsudd, Kurt F.; Blomberg, Stefan I. E. | 2004 |
| Dose-response and efficacy of spinal manipulation for care of chronic low back pain: a randomized controlled trial | Haas, Mitchell; Vavrek, Darcy; Peterson, David; Polissar, Nayak; Neradilek, Moni B. | 2014 |
| Manipulative Therapy and Clinical Prediction Criteria in Treatment of Acute Nonspecific Low Back Pain | Hallegraeff, H. J. M.; Winters, Jan C.; de Greef, Mathieu; Lucas, Gees | 2009 |
| Assessment of diclofenac or spinal manipulative therapy, or both, in addition to recommended first-line treatment for acute low back pain: a randomised controlled trial | Hancock, Mark J; Maher, Chris G; Latimer, Jane; McLachlan, Andrew J; Cooper, Chris W; Day, Richard O; Spindler, Megan F; McAuley, James H | 2007 |
| Manual therapy followed by specific active exercises versus a placebo followed by specific active exercises on the improvement of functional disability in patients with chronic non specific low back pain: a randomized controlled trial | Balthazard, Pierre; de Goumoens, Pierre; Rivier, Gilles; Demeulenaere, Philippe; Ballabeni, Pierluigi; Dériaz, Olivier | 2012 |
| Efficacy of Treating Low Back Pain and Dysfunction Secondary to Osteoarthritis: Chiropractic Care Compared With Moist Heat Alone | Beyerman, Kathleen L.; Palmerino, Mark B.; Zohn, Lee E.; Kane, Gary M.; Foster, Kathy A. | 2006 |
| Spinal Manipulative Therapy–Specific Changes in Pain Sensitivity in Individuals With Low Back Pain (NCT01168999) | Bialosky, Joel E.; George, Steven Z.; Horn, Maggie E.; Price, Donald D.; Staud, Roland; Robinson, Michael E. | 2014 |
| Effect of spinal manipulative therapy on mechanical pain sensitivity in patients with chronic nonspecific low back pain: a pilot randomized, controlled trial | Bond, Bryan M.; Kinslow, Chris D.; Yoder, Adam W.; Liu, Wen | 2020 |
| Supervised exercise, spinal manipulation, and home exercise for chronic low back pain: a randomized clinical trial | Bronfort, Gert; Maiers, Michele J.; Evans, Roni L.; Schulz, Craig A.; Bracha, Yiscah; Svendsen, Kenneth H.; Grimm, Richard H.; Owens, Edward F.; Garvey, Timothy A.; Transfeldt, Ensor E. | 2011 |
| Short-term effectiveness of spinal manipulative therapy versus functional technique in patients with chronic nonspecific low back pain: a pragmatic randomized controlled trial | Castro-Sánchez, Adelaida María; Lara-Palomo, Inmaculada C.; Matarán-Peñarrocha, Guillermo A.; Fernández-de-las-Peñas, César; Saavedra-Hernández, Manuel; Cleland, Joshua; Aguilar-Ferrándiz, María Encarnación | 2016 |
| A prospective study of patients with chronic back pain randomised to group exercise, physiotherapy or osteopathy | Chown, Marjorie; Whittamore, Lynne; Rush, Mark; Allan, Sally; Stott, David; Archer, Mark | 2008 |
| Early use of thrust manipulation versus non-thrust manipulation: A randomized clinical trial | Cook, Chad; Learman, Kenneth; Showalter, Chris; Kabbaz, Vincent; O'Halloran, Bryan | 2013 |
| Osteopathic manipulation treatment versus therapeutic exercises in patients with chronic nonspecific low back pain: A randomized, controlled and double-blind study | de Oliveira Meirelles, Frederico; de Oliveira Muniz Cunha, Júlio César; da Silva, Elirez Bezerra | 2020 |
| Directed vertebral manipulation is not better than generic vertebral manipulation in patients with chronic low back pain: a randomised trial | de Oliveira, Ronaldo Fernando; Costa, Leonardo Oliveira Pena; Nascimento, Leonardo Penteado; Rissato, Lívia Leticia | 2020 |
| The effect of spinal manipulation on brain neurometabolites in chronic nonspecific low back pain patients: a randomized clinical trial | Didehdar, Daryoush; Kamali, Fahimeh; Yoosefinejad, Amin Kordi; Lotfi, Mehrzad | 2020 |
| Spinal Manipulative Therapy for Chronic Lower Back Pain in Older Veterans: A Prospective, Randomized, Placebo-Controlled Trial | Dougherty, Paul E.; Karuza, Jurgis; Dunn, Andrew S.; Savino, Dorian; Katz, Paul | 2014 |
| Immediate effects of spinal manipulation in pain and global joint mobility in patients with chronic nonspecific low back pain | Fernandes, Walkyria Vilas Boas; Pizzol, Franciéli Dall; Capote, Andrielle Elaine; Melo, Sibele de Andrade; Schleder, Juliana Carvalho | 2016 |
| Effectiveness of mechanical diagnosis and therapy in patients with back pain who meet a clinical prediction rule for spinal manipulation | Schenk, Ron; Dionne, Carol; Simon, Corey; Johnson, Robert | 2012 |
| Short term modulation of trunk neuromuscular responses following spinal manipulation: a control group study | Harvey, Marie-Pierre; Descarreaux, Martin | 2013 |
| Similar Effects of Thrust and Nonthrust Spinal Manipulation Found in Adults With Subacute and Chronic Low Back Pain: A Controlled Trial With Adaptive Allocation | Xia, Ting; Long, Cynthia R.; Gudavalli, Maruti R.; Wilder, David G.; Vining, Robert D.; Rowell, Robert M.; Reed, William R.; DeVocht, James W.; Goertz, Christine M.; Owens, Edward F.; Meeker, William C. | 2016 |
| A randomized, controlled trial of osteopathic manipulative treatment for acute low back pain in active duty military personnel | Cruser, des Anges; Maurer, Douglas; Hensel, Kendi; Brown, Sarah K.; White, Kathryn; Stoll, Scott T. | 2012 |
| Effectiveness of four conservative treatments for subacute low back pain: a randomized clinical trial | Hsieh, Chang-Yu J.; Adams, Alan H.; Tobis, Jerome; Hong, Chang-Zern; Danielson, Clark; Platt, Katherine; Hoehler, Fred; Reinsch, Sibylle; Rubel, Arthur | 2002 |
| A Randomized Controlled Trial Comparing 2 Types of Spinal Manipulation and Minimal Conservative Medical Care for Adults 55 Years and Older With Subacute or Chronic Low Back Pain | Hondras, Maria A.; Long, Cynthia R.; Cao, Ying; Rowell, Robert M.; Meeker, William C. | 2009 |
| Comparison of general exercise, motor control exercise and spinal manipulative therapy for chronic low back pain: A randomized trial | Ferreira, Manuela L.; Ferreira, Paulo H.; Latimer, Jane; Herbert, Robert D.; Hodges, Paul W.; Jennings, Matthew D.; Maher, Christopher G.; Refshauge, Kathryn M. | 2007 |
| Spinal manipulation compared with back school and with individually delivered physiotherapy for the treatment of chronic low back pain: a randomized trial with one-year follow-up | Cecchi, Francesca; Molino-Lova, Raffaello; Chiti, Massimiliano; Pasquini, Guido; Paperini, Anita; Conti, Andrea A; Macchi, Claudio | 2010 |
| Manual therapy versus therapeutic exercise in non-specific chronic neck pain: a randomized controlled trial | Bernal-Utrera, Carlos; Gonzalez-Gerez, Juan Jose; Anarte-Lazo, Ernesto; Rodriguez-Blanco, Cleofas | 2020 |
| Influence of the cervical spine manipulation in the neck disability index in patients with chronic neck pain: A preliminary study | Miranda, Iã Ferreira; Facchini, Daniel; Manfio, Eliane Fátima | 2015 |
| Chronic spinal pain syndromes: a clinical pilot trial comparing acupuncture, a nonsteroidal anti-inflammatory drug, and spinal manipulation | Giles, L. G.; Müller, R. | 1999 |
| Osteopathic manipulative treatment combined with exercise improves pain and disability in individuals with non-specific chronic neck pain: A pragmatic randomized controlled trial | Groisman, Sandro; Malysz, Tais; de Souza da Silva, Luciano; Rocha Ribeiro Sanches, Tamara; Camargo Bragante, Karoline; Locatelli, Franciele; Pontel Vigolo, Cleder; Vaccari, Simone; Homercher Rosa Francisco, Cristina; Monteiro Steigleder, Simone; Jotz, Geraldo Pereira | 2020 |
| Intensive training, physiotherapy, or manipulation for patients with chronic neck pain. A prospective, single-blinded, randomized clinical trial | Jordan, A.; Bendix, T.; Nielsen, H.; Hansen, F. R.; Høst, D.; Winkel, A. | 1998 |
| Effect of thoracic manipulation and deep craniocervical flexor training on pain, mobility, strength, and disability of the neck of patients with chronic nonspecific neck pain: a randomized clinical trial | Lee, Kwan-Woo; Kim, Won-Ho | 2016 |
| The effect of spinal manipulation on the efficacy of a rehabilitation protocol for patients with chronic neck pain: a pilot study | Murphy, Bernadette; Taylor, Heidi Haavik; Marshall, Paul | 2010 |
| Immediate and Short-Term Effects of Upper Thoracic Manipulation on Myoelectric Activity of Sternocleidomastoid Muscles in Young Women With Chronic Neck Pain: A Randomized Blind Clinical Trial | Pires, Paulo Fernandes; Packer, Amanda Carine; Dibai-Filho, Almir Vieira; Rodrigues-Bigaton, Delaine | 2015 |
| Acute effects of single and multiple level thoracic manipulations on chronic mechanical neck pain: a randomized controlled trial | Puntumetakul, Rungthip; Suvarnnato, Thavatchai; Werasirirat, Phurichaya; Uthaikhup, Sureeporn; Yamauchi, Junichiro; Boucaut, Rose | 2015 |
| Does the Addition of Manual Therapy Approach to a Cervical Exercise Program Improve Clinical Outcomes for Patients with Chronic Neck Pain in Short- and Mid-Term? A Randomized Controlled Trial | Rodríguez-Sanz, Jacobo; Malo-Urriés, Miguel; Corral-de-Toro, Jaime; López-de-Celis, Carlos; Lucha-López, María Orosia; Tricás-Moreno, José Miguel; Lorente, Ana I.; Hidalgo-García, César | 2020 |
| Effects of the Manual Therapy Approach of Segments C0-1 and C2-3 in the Flexion-Rotation Test in Patients with Chronic Neck Pain: A Randomized Controlled Trial | Rodríguez-Sanz, Jacobo; Malo-Urriés, Miguel; Lucha-López, María Orosia; Pérez-Bellmunt, Albert; Carrasco-Uribarren, Andoni; Fanlo-Mazas, Pablo; Corral-de-Toro, Jaime; Hidalgo-García, César | 2021 |
| Endocrine response after cervical manipulation and mobilization in people with chronic mechanical neck pain: a randomized controlled trial | Valera-Calero, Antonio; Lluch Girbés, Enrique; Gallego-Izquierdo, Tomás; Malfliet, Anneleen; Pecos-Martín, Daniel | 2019 |
| The effectiveness of manual physical therapy and exercise for mechanical neck pain: a randomized clinical trial | Walker, Michael J.; Boyles, Robert E.; Young, Brian A.; Strunce, Joseph B.; Garber, Matthew B.; Whitman, Julie M.; Deyle, Gail; Wainner, Robert S. | 2008 |
| Changes in proprioception and pain in patients with neck pain after upper thoracic manipulation | Yang, Jinmo; Lee, Byoungkwon; Kim, Changbeom | 2015 |
| The effect of two weeks of spinal manipulative therapy and home stretching exercises on pain and disability in patients with persistent or recurrent neck pain; a randomized controlled trial | Bakken, Anders Galaasen; Eklund, Andreas; Warnqvist, Anna; O’Neill, Søren; Axén, Iben | 2021 |
| Immediate Effects on Neck Pain and Active Range of Motion After a Single Cervical High-Velocity Low-Amplitude Manipulation in Subjects Presenting with Mechanical Neck Pain: A Randomized Controlled Trial | Martínez-Segura, Raquel; Fernández-de-las-Peñas, César; Ruiz-Sáez, Mariana; López-Jiménez, Cristina; Rodríguez-Blanco, Cleofás | 2006 |
| Thoracic Spine Manipulation for the Management of Patients With Neck Pain: A Randomized Clinical Trial | González-Iglesias, Javier; Fernández-de-las-Peñas, Cesar; Cleland, Joshua A.; del Rosario Gutiérrez-Vega, Maria | 2009 |
| A Randomized Controlled Trial Comparing Manipulation With Mobilization for Recent Onset Neck Pain | Leaver, Andrew M.; Maher, Christopher G.; Herbert, Robert D.; Latimer, Jane; McAuley, James H.; Jull, Gwendolen; Refshauge, Kathryn M. | 2010 |
| Thoracic Spine Thrust Manipulation Versus Cervical Spine Thrust Manipulation in Patients With Acute Neck Pain : A Randomized Clinical Trial | Puentedura, Emilio J.; Landers, Merrill R.; Cleland, Joshua A.; Mintken, Paul; Huijbregts, Peter; Fernandez-De-Las-Peñas, Cesar | 2011 |
| Short-Term Combined Effects of Thoracic Spine Thrust Manipulation and Cervical Spine Nonthrust Manipulation in Individuals With Mechanical Neck Pain: A Randomized Clinical Trial | Masaracchio, Michael; Cleland, Joshua; Hellman, Madeleine; Hagins, Marshall | 2013 |
| Intramuscular Ketorolac Versus Osteopathic Manipulative Treatment in the Management of Acute Neck Pain in the Emergency Department: A Randomized Clinical Trial | McReynolds, Tamara M.; Sheridan, Barry J. | 2005 |
| Effects of Upper and Lower Cervical Spinal Manipulative Therapy on Blood Pressure and Heart Rate Variability in Volunteers and Patients With Neck Pain: A Randomized Controlled, Cross-Over, Preliminary Study | Win, Ni Ni; Jorgensen, Anna Maria S.; Chen, Yu Sui; Haneline, Michael T. | 2015 |
| A clinical trial investigating the possible effect of the supine cervical rotatory manipulation and the supine lateral break manipulation in the treatment of mechanical neck pain: a pilot study | van Schalkwyk, R.; Parkin-Smith, G. F. | 2000 |
| A feasibility study assessing manual therapies to different regions of the spine for patients with subacute or chronic neck pain | Strunk, Richard G.; Hondras, Maria A. | 2008 |
| A pilot randomized clinical trial on the relative effect of instrumental (MFMA) versus manual (HVLA) manipulation in the treatment of cervical spine dysfunction | Wood, T. G.; Colloca, C. J.; Matthews, R. | 2001 |
| A randomised controlled trial of preventive spinal manipulation with and without a home exercise program for patients with chronic neck pain | Martel, Johanne; Dugas, Claude; Dubois, Jean-Daniel; Descarreaux, Martin | 2011 |
| Improvement after chiropractic care in cervicocephalic kinesthetic sensibility and subjective pain intensity in patients with nontraumatic chronic neck pain | Palmgren, Per J.; Sandström, Peter J.; Lundqvist, Fredrik J.; Heikkilä, Hannu | 2006 |
| Chiropractic manipulative therapy and low-level laser therapy in the management of cervical facet dysfunction: a randomized controlled study | Saayman, Lindie; Hay, Caroline; Abrahamse, Heidi | 2011 |
| Short-term effects of spinal thrust joint manipulation in patients with chronic neck pain: a randomized clinical trial | Saavedra-Hernández, Manuel; Arroyo-Morales, Manuel; Cantarero-Villanueva, Irene; Fernández-Lao, Carolina; Castro-Sánchez, Adelaida M.; Puentedura, Emilio J.; Fernández-de-las-Peñas, César | 2013 |
| Supervised exercise with and without spinal manipulation performs similarly and better than home exercise for chronic neck pain: a randomized controlled trial | Evans, Roni; Bronfort, Gert; Schulz, Craig; Maiers, Michele; Bracha, Yiscah; Svendsen, Kenneth; Grimm, Richard; Garvey, Timothy; Transfeldt, Ensor | 2012 |
| Comparison of the effectiveness of three manual physical therapy techniques in a subgroup of patients with low back pain who satisfy a clinical prediction rule: a randomized clinical trial | Cleland, Joshua A.; Fritz, Julie M.; Kulig, Kornelia; Davenport, Todd E.; Eberhart, Sarah; Magel, Jake; Childs, John D. | 2009 |
| Mobilization versus manipulations versus sustain apophyseal natural glide techniques and interaction with psychological factors for patients with chronic neck pain: randomized controlled trial | Lopez-Lopez, A.; Alonso Perez, J. L.; González Gutierez, J. L.; La Touche, R.; Lerma Lara, S.; Izquierdo, H.; Fernández-Carnero, J. | 2015 |
| Role of manual therapy with exercise regime versus exercise regime alone in the management of non-specific chronic neck pain | Akhter, Saeed; Khan, Muhammad; Ali, Syed Shahzad; Soomro, Rabial Rani | 2014 |
| Immediate effects of thoracic manipulation in patients with neck pain: a randomized clinical trial | Cleland, Joshua A.; Childs, John D.; McRae, Meghann; Palmer, Jessica A.; Stowell, Thomas | 2005 |
| Is a behavioral graded activity program more effective than manual therapy in patients with subacute neck pain? Results of a randomized clinical trial | Pool, Jan J. M.; Ostelo, Raymond W. J. G.; Knol, Dirk L.; Vlaeyen, Johan W. S.; Bouter, Lex M.; de Vet, Henrica C. W. | 2010 |
| Osteopathic treatment of patients with chronic non-specific neck pain: a randomised controlled trial of efficacy | Schwerla, Florian; Bischoff, Anne; Nurnberger, Andrea; Genter, Pia; Guillaume, Jean-Pierre; Resch, Karl-Ludwig | 2008 |
| A randomized clinical trial of exercise and spinal manipulation for patients with chronic neck pain | Bronfort, G.; Evans, R.; Nelson, B.; Aker, P. D.; Goldsmith, C. H.; Vernon, H. | 2001 |
| Two-year follow-up of a randomized clinical trial of spinal manipulation and two types of exercise for patients with chronic neck pain | Evans, Roni; Bronfort, Gert; Nelson, Brian; Goldsmith, Charles H. | 2002 |
| Short-term effects of thrust versus nonthrust mobilization/manipulation directed at the thoracic spine in patients with neck pain: a randomized clinical trial | Cleland, Joshua A.; Glynn, Paul; Whitman, Julie M.; Eberhart, Sarah L.; MacDonald, Cameron; Childs, John D. | 2007 |
| Inclusion of thoracic spine thrust manipulation into an electro-therapy/thermal program for the management of patients with acute mechanical neck pain: a randomized clinical trial | González-Iglesias, Javier; Fernández-de-las-Peñas, Cesar; Cleland, Joshua A.; Alburquerque-Sendín, Francisco; Palomeque-del-Cerro, Luis; Méndez-Sánchez, Roberto | 2009 |
| Functional Magnetic Resonance Imaging of Cerebral Hemodynamic Responses to Pain Following Thoracic Thrust Manipulation in Individuals With Neck Pain: A Randomized Trial | Sparks, Cheryl L.; Liu, Wen C.; Cleland, Joshua A.; Kelly, Joseph P.; Dyer, Sarah J.; Szetela, Kathryn M.; Elliott, James M. | 2017 |
| Immediate effects of a thoracic spine thrust manipulation on the autonomic nervous system: a randomized clinical trial | Sillevis, Rob; Cleland, Joshua; Hellman, Madeleine; Beekhuizen, Kristina | 2010 |
| Orthopaedic manual therapy, McKenzie method or advice only for low back pain in working adults: a randomized controlled trial with one year follow-up | Paatelma, Markku; Kilpikoski, Sinikka; Simonen, Riitta; Heinonen, Ari; Alen, Markku; Videman, Tapio | 2008 |
| Spinal manipulation plus laser therapy versus laser therapy alone in the treatment of chronic non-specific low back pain: a randomized controlled study | Nambi, Gopal; Kamal, Walid; Es, Shanmugananth; Joshi, Siddhart; Trivedi, Parth | 2018 |
| A comparison of osteopathic spinal manipulation with standard care for patients with low back pain | Andersson, G. B.; Lucente, T.; Davis, A. M.; Kappler, R. E.; Lipton, J. A.; Leurgans, S. | 1999 |
| Manual therapy and exercise therapy in patients with chronic low back pain: a randomized, controlled trial with 1-year follow-up | Aure, Olav Frode; Nilsen, Jens Hoel; Vasseljen, Ottar | 2003 |
| Immediate effects of a high-velocity spine manipulation in paraspinal muscles activity of nonspecific chronic low-back pain subjects | Bicalho, Eduardo; Setti, João Antônio Palma; Macagnan, Jones; Cano, José Luis Rivas; Manffra, Elisangela Ferretti | 2010 |
| A controlled, multicentre trial of manual therapy in low-back pain. Initial status, sick-leave and pain score during follow-up | Blomberg, S.; Svärdsudd, K.; Mildenberger, F. | 1992 |
| Trunk exercise combined with spinal manipulative or NSAID therapy for chronic low back pain: a randomized, observer-blinded clinical trial | Bronfort, G.; Goldsmith, C. H.; Nelson, C. F.; Boline, P. D.; Anderson, A. V. | 1996 |
| Spinal manipulation, medication, or home exercise with advice for acute and subacute neck pain: a randomized trial | Bronfort, Gert; Evans, Roni; Anderson, Alfred V.; Svendsen, Kenneth H.; Bracha, Yiscah; Grimm, Richard H. | 2012 |
| Spinal manipulation and home exercise with advice for subacute and chronic back-related leg pain: a trial with adaptive allocation | Bronfort, Gert; Hondras, Maria A.; Schulz, Craig A.; Evans, Roni L.; Long, Cynthia R.; Grimm, Richard | 2014 |
| Single-blind randomised controlled trial of chemonucleolysis and manipulation in the treatment of symptomatic lumbar disc herniation | Burton, A. K.; Tillotson, K. M.; Cleary, J. | 2000 |
| The immediate effect of manipulation versus mobilization on pain and range of motion in the cervical spine: a randomized controlled trial | Cassidy, J. D.; Lopes, A. A.; Yong-Hing, K. | 1992 |
| A comparison of physical therapy, chiropractic manipulation, and provision of an educational booklet for the treatment of patients with low back pain | Cherkin, D. C.; Deyo, R. A.; Battié, M.; Street, J.; Barlow, W. | 1998 |
| The Hmax/Mmax ratio as an outcome measure for acute low back pain | Cramer, G. D.; Humphreys, C. R.; Hondras, M. A.; McGregor, M.; Triano, J. J. | 1993 |
| Upper cervical and upper thoracic manipulation versus mobilization and exercise in patients with cervicogenic headache: a multi-center randomized clinical trial | Dunning, James R.; Butts, Raymond; Mourad, Firas; Young, Ian; Fernandez-de-Las Peñas, Cesar; Hagins, Marshall; Stanislawski, Thomas; Donley, Jonathan; Buck, Dustin; Hooks, Todd R.; Cleland, Joshua A. | 2016 |
| Acute low back pain. Comparison of two conservative treatment approaches | Farrell, J. P.; Twomey, L. T. | 1982 |
| Efficacy of classification-based cognitive functional therapy in patients with non-specific chronic low back pain: a randomized controlled trial | Vibe Fersum, K.; O'Sullivan, P.; Skouen, J. S.; Smith, A.; Kvåle, A. | 2013 |
| Chronic spinal pain: a randomized clinical trial comparing medication, acupuncture, and spinal manipulation | Giles, Lynton G. F.; Muller, Reinhold | 2003 |
| Back pain: a randomized clinical trial of rotational manipulation of the trunk | Glover, J. R.; Morris, J. G.; Khosla, T. | 1974 |
| Dose response and efficacy of spinal manipulation for chronic cervicogenic headache: a pilot randomized controlled trial | Haas, Mitchell; Spegman, Adele; Peterson, David; Aickin, Mikel; Vavrek, Darcy | 2010 |
| Dose-response and efficacy of spinal manipulation for care of cervicogenic headache: a dual-center randomized controlled trial | Haas, Mitchell; Bronfort, Gert; Evans, Roni; Schulz, Craig; Vavrek, Darcy; Takaki, Leslie; Hanson, Linda; Leininger, Brent; Neradilek, Moni B. | 2018 |
| A benefit of spinal manipulation as adjunctive therapy for acute low-back pain: a stratified controlled trial | Hadler, N. M.; Curtis, P.; Gillings, D. B.; Stinnett, S. | 1987 |
| Effects of different treatment modalities on gait symmetry and clinical measures for sacroiliac joint patients | Herzog, W.; Conway, P. J.; Willcox, B. J. | 1991 |
| Functional outcomes of low back pain: comparison of four treatment groups in a randomized controlled trial | Hsieh, C. Y.; Phillips, R. B.; Adams, A. H.; Pope, M. H. | 1992 |
| A randomized trial of medical care with and without physical therapy and chiropractic care with and without physical modalities for patients with low back pain: 6-month follow-up outcomes from the UCLA low back pain study | Hurwitz, Eric L.; Morgenstern, Hal; Harber, Philip; Kominski, Gerald F.; Belin, Thomas R.; Yu, Fei; Adams, Alan H.; University of California-Los Angeles | 2002 |
| A randomized trial of chiropractic manipulation and mobilization for patients with neck pain: clinical outcomes from the UCLA neck-pain study | Hurwitz, Eric L.; Morgenstern, Hal; Harber, Philip; Kominski, Gerald F.; Yu, Fei; Adams, Alan H. | 2002 |
| A randomized controlled trial of exercise and manipulative therapy for cervicogenic headache | Jull, Gwendolen; Trott, Patricia; Potter, Helen; Zito, Guy; Niere, Ken; Shirley, Debra; Emberson, Jonathan; Marschner, Ian; Richardson, Carolyn | 2002 |
| The effectiveness of manual therapy, physiotherapy, and treatment by the general practitioner for nonspecific back and neck complaints. A randomized clinical trial | Koes, B. W.; Bouter, L. M.; van Mameren, H.; Essers, A. H.; Verstegen, G. M.; Hofhuizen, D. M.; Houben, J. P.; Knipschild, P. G. | 1992 |
| The effectiveness of Long's manipulation on patients with chronic mechanical neck pain: a randomized controlled trial | Lin, Jian Hua; Shen, Tong; Chung, Raymond Chi Keung; Chiu, Thomas Tai Wing | 2013 |
| Manipulation or microdiskectomy for sciatica? A prospective randomized clinical study | McMorland, Gordon; Suter, Esther; Casha, Steve; du Plessis, Stephan J.; Hurlbert, R. John | 2010 |
| Manipulative therapy in the treatment of benign cervicobrachialgia of mechanical origin | Moretti, B.; Vetro, A.; Garofalo, R.; Moretti, L.; Patella, S.; Patella, V.; Simone, C. | 2004 |
| Long-term follow-up of a randomized clinical trial assessing the efficacy of medication, acupuncture, and spinal manipulation for chronic mechanical spinal pain syndromes | Muller, Reinhold; Giles, Lynton G. F. | 2005 |
| A pilot randomized controlled trial comparing the efficacy of exercise, spinal manipulation, and neuro emotional technique for the treatment of pregnancy-related low back pain | Peterson, Caroline D.; Haas, Mitchell; Gregory, W. Thomas | 2012 |
| A prospective randomized three-week trial of spinal manipulation, transcutaneous muscle stimulation, massage and corset in the treatment of subacute low back pain | Pope, M. H.; Phillips, R. B.; Haugh, L. D.; Hsieh, C. Y.; MacDonald, L.; Haldeman, S. | 1994 |
| Chiropractic adjustive manipulation on subjects with acute low back pain: visual analog pain scores and plasma beta-endorphin levels | Sanders, G. E.; Reinert, O.; Tepe, R.; Maloney, P. | 1990 |
| Chiropractic manipulation in the treatment of acute back pain and sciatica with disc protrusion: a randomized double-blind clinical trial of active and simulated spinal manipulations | Santilli, Valter; Beghi, Ettore; Finucci, Stefano | 2006 |
| Active or passive treatment for neck-shoulder pain in occupational health care? A randomized controlled trial | Savolainen, Aslak; Ahlberg, Jari; Nummila, Hanna; Nissinen, Maunu | 2004 |
| Osteopathic Manipulative Therapy in Women With Postpartum Low Back Pain and Disability: A Pragmatic Randomized Controlled Trial | Schwerla, Florian; Rother, Katrin; Rother, Denis; Ruetz, Michaela; Resch, Karl-Ludwig | 2015 |
| A randomized clinical trial of manual versus mechanical force manipulation in the treatment of sacroiliac joint syndrome | Shearar, Kirstin A.; Colloca, Christopher J.; White, Horace L. | 2005 |
| Manipulation for chronic neck pain. A double-blind controlled study | Sloop, P. R.; Smith, D. S.; Goldenberg, E.; Doré, C. | 1982 |
| Manipulative therapy versus education programs in chronic low back pain | Triano, J. J.; McGregor, M.; Hondras, M. A.; Brennan, P. C. | 1995 |
| Validation of a novel sham cervical manipulation procedure | Vernon, Howard T.; Triano, John J.; Ross, James K.; Tran, Steven K.; Soave, David M.; Dinulos, Maricelle D. | 2012 |
| Short-term usual chiropractic care for spinal pain: a randomized controlled trial | Walker, Bruce F.; Hebert, Jeff J.; Stomski, Norman J.; Losco, Barrett; French, Simon D. | 2013 |
| An open study of diflunisal, conservative and manipulative therapy in the management of acute mechanical low back pain | Waterworth, R. F.; Hunter, I. A. | 1985 |
| Dorsal manipulation in whiplash injury treatment: a randomized controlled trial | Fernandez-de-las-Penas, , C, Fernandez-Carnero, J, Plaza Fernandez, A, Lomas-Vega, R; Miangolarra-Page, JC | 2004 |
| Benefits of Thoracic Thrust Manipulation when Applied with a Multi-Modal Treatment Approach in Individuals with Mechanical Neck Pain: A Pilot Randomized Trial | Khoja, Samannaaz; Browder, David; Daliman, Daniel; Piva, Sara | 2015 |
| Manipulation in the treatment of acute low back pain | Morton, JE | 1999 |
| A comparison of spinal manipulation methods and usual medical care for low back pain: a randomized clinical trial | Schneider, , MJ, Haas, M, Glick, RM, Stevans, JM; Landsittel, D | 2014 |
| Effectiveness of low back pain manipulative therapy in combination with physical therapy as compared to standard physical therapy | Venegas-Rios, HL | 2009 |
| Short term trial of chiropractic adjustments for the relief of chronic low back pain | Waagen, , GN, Haldeman, S, Cook, G, Lopez, D; DeBoer, KF | 1986 |
| Comparison of two chiropractic techniques on pain and lateral flexion in neck pain patients: a pilot study | Yurkiw, , D; Mior, S | 1996 |
| Immediate Effects of Osteopathic Treatment Versus Therapeutic Exercise on Patients With Chronic Cervical Pain | Galindez-Ibarbengoetxea, X; Setuain, I; Ramírez-Velez, R; Andersen, LL; González-Izal, M; Jauregi, A; Izquierdo, M | 2018 |
| Immediate and Short-term Effects of Thoracic Spine Manipulation in Patients With Cervical Radiculopathy: a Randomized Controlled Trial | Young, IA; Pozzi, F; Dunning, J; Linkonis, R; Michener, LA | 2019 |
| Effects of Instrumental, Manipulative and Soft Tissue Approaches for the Suboccipital Region in Subjects with Chronic Mechanical Neck Pain. A Randomized Controlled Trial | Arjona Retamal, JJ; Fernández Seijo, A; Torres Cintas, JD; de-la-Llave-Rincón, AI; Caballero Bragado, A | 2021 |
| Pragmatic application of manipulation versus mobilization to the upper segments of the cervical spine plus exercise for treatment of cervicogenic headache: a randomized clinical trial | Lerner-Lentz, A; O'Halloran, B; Donaldson, M; Cleland, JA | 2021 |
| Comparison of an exercise program with and without manual therapy for patients with chronic neck pain and upper cervical rotation restriction. Randomized controlled trial | Rodriguez-Sanz, J; Malo-Urries, M; Lucha-Lopez, MO; Lopez-De-Celis, C; Perez-Bellmunt, A; Corral-De-Toro, J; Hidalgo-Garcia, C | 2021 |
| Individualised manual therapy plus guideline-based advice vs advice alone for people with clinical features of lumbar zygapophyseal joint pain: a randomised controlled trial | Ford, JJ; Slater, SL; Richards, MC; Surkitt, LD; Chan, AYP; Taylor, NF; Hahne, AJ | 2019 |
| Spinal manipulation and perineural electrical dry needling in patients with cervicogenic headache: a multicenter randomized clinical trial | Dunning, J; Butts, R; Zacharko, N; Fandry, K; Young, I; Wheeler, K; Day, J; Fernández-de-Las-Peñas, C | 2021 |
| Effectiveness of Exercise Therapy and Manipulation on Sacroiliac Joint Dysfunction: a Randomized Controlled Trial | Nejati, P; Safarcherati, A; Karimi, F | 2019 |
| Optimization of Spinal Manipulative Therapy Protocols: a Factorial Randomized Trial Within a Multiphase Optimization Framework | Fritz, JM; Sharpe, J; Greene, T; Lane, E; Hadizadeh, M; McFadden, M; Santillo, D; Farley, J; Magel, J; Thackeray, A; et al. | 2021 |
| A Single-Center Study Comparing the Effects of Thoracic Spine Manipulation vs Mobility Exercises in 26 Office Workers with Chronic Neck Pain: a Randomized Controlled Clinical Study | Seo, J; Song, C; Shin, D | 2022 |
| The Efficiency of Manual Therapy and Sacroiliac and Lumbar Exercises in Patients with Sacroiliac Joint Dysfunction Syndrome | Javadov, A; Ketenci, A; Aksoy, C | 2021 |
| The comparative effects of spinal manipulation, myofascial release and exercise in tension-type headache patients with neck pain: a randomized controlled trial | Corum, M; Aydin, T; Medin Ceylan, C; Kesiktas, FN | 2021 |
| Manipulative therapy of sacral torsion versus myofascial release in patients clinically diagnosed posterior pelvic pain: a consort compliant randomized controlled trial | Castro-Sánchez, AM; Gil-Martínez, E; Fernández-Sánchez, M; Lara-Palomo, IC; Nastasia, I; de Los Ángeles Querol-Zaldívar, M; Aguilar-Ferrándiz, ME | 2021 |
| Fascial treatment versus manual therapy (HVLA) in patients with chronic neck pain: a randomized controlled trial | Brück, K; Jacobi, K; Schmidt, T | 2021 |
| A study to observe the effects of physiotherapy with and without manual therapy in the management postural neck pain: a randomized control trial | Nasir, MF; Jawed, R; Baig, NN; Younus, M; Arshad, A; Tahir, A | 2021 |
| Effects of spinal manipulative therapy biomechanical parameters on clinical and biomechanical outcomes of participants with chronic thoracic pain: a randomized controlled experimental trial | Pagé, I; Descarreaux, M | 2019 |
| Short-term effectiveness of the flexion-distraction technique in comparison with high-velocity vertebral manipulation in patients suffering from low-back pain | Carrasco-Martínez, F; Ibáñez-Vera, AJ; Martínez-Amat, A; Hita-Contreras, F; Lomas-Vega, R | 2019 |
| Spinal Manipulation for Subacute and Chronic Lumbar Radiculopathy: a Randomized Controlled Trial | Ghasabmahaleh, SH; Rezasoltani, Z; Dadarkhah, A; Hamidipanah, S; Mofrad, RK; Najafi, S | 2021 |
| Effects of thoracic spine manipulation on pressure pain sensitivity of rhomboid muscle active trigger points: a randomized controlled trial | Haleema, B; Riaz, H | 2021 |
| Thoracic spine thrust manipulation for individuals with cervicogenic headache: a crossover randomized clinical trial | McDevitt, AW; Cleland, JA; Rhon, DI; Altic, RAK; Courtney, DJ; Glynn, PE; Mintken, PE | 2022 |
| Clinical Efficacy and Safety of "Three-Dimensional Balanced Manipulation" in the Treatment of Cervical Spondylotic Radiculopathy by Finite Element Analysis | Cao, S; Chen, Y; Zhang, F; Sun, S; Wang, C; Hou, G; Wang, D; Sun, G; Shi, B | 2021 |
| Multidisciplinary integrative care versus chiropractic care for low back pain: a randomized clinical trial | Bronfort, G; Maiers, M; Schulz, C; Leininger, B; Westrom, K; Angstman, G; Evans, R | 2022 |
| Chiropractic management of dominating one-sided pelvic girdle pain in pregnant women; a randomized controlled trial | Gausel, AM; Kjærmann, I; Malmqvist, S; Andersen, K; Dalen, I; Larsen, JP; Økland, I | 2017 |
| Adding Chiropractic Treatment to Individual Rehabilitation for Persistent Pelvic Girdle Pain 3 to 6 Months After Delivery: a Pilot Randomized Trial | Gausel, AM; Dalen, I; Kjærmann, I; Malmqvist, S; Andersen, K; Larsen, JP; Økland, I | 2019 |
| Comparing the effect of thrust manipulation and muscle energy technique on pain and disability in patients with sacroiliac joint dysfunction | Fatima, I; Goyal, M; Singh, G | 2021 |
| Duration of the effects of spinal manipulation on pain intensity and electromyographic activity of paravertebral parts of individuals with chronic mechanical low back pain | Boas Fernandes, WV; Silveira Bicalho, E; Capote, AE; Ferretti Manffra, E | 2016 |
| Spine manipulation for high-speed in professional nursing with chronic low back pain | Faitão, CA; Fernandes, WVB | 2011 |
| Vertebral manipulation in chronic low back pain: a prospective randomised study of 85 cases | Elleuch, M; Ghroubi, S | 2009 |
| A randomised, placebo-controlled, clinical trial for the short-term effectiveness of manipulative therapy and acupuncture on pain caused by mechanical thoracic spine dysfunction | Lehtola, V; Korhonen, I; Airaksinen, O | 2010 |
| Cognitive functional therapy compared with core exercise and manual therapy in patients with chronic low back pain: randomised controlled trial | Castro, J; Correia, L; Donato, BS; Arruda, B; Agulhari, F; Pellegrini, MJ; Belache, FTC; de Souza, CP; Fernandez, J; Nogueira, LAC; et al. | 2022 |
| Effect of osteopathic manipulation of the sacroiliac joint vs electrotherapy on pain and functional disability in patients with low back pain: a pilot study | Rodriguez-Pastor, JA; Caro-Puertolas, B; Cana-Pino, A; Sanchez-Preciado, AM; Garrido-Ardila, EM; Apolo-Arenas, MD | 2022 |
| Randomized trial of general strength and conditioning versus motor control and manual therapy for chronic low back pain on physical and self-report outcomes | Tagliaferri, SD; Miller, CT; Ford, JJ; Hahne, AJ; Main, LC; Rantalainen, T; Connell, DA; Simson, KJ; Owen, PJ; Belavy, DL | 2020 |
| Comparison of manipulation and stabilization exercises in patients with sacroiliac joint dysfunction patients: a randomized clinical trial | Kamali, F; Zamanlou, M; Ghanbari, A; Alipour, A; Bervis, S | 2018 |
| Effect of thrust versus non-thrust mobilization directed at the thoracic spine in patients with mechanical neck pain: a randomized control trial | Harihara Prakash R; Mehta J; Patel D | 2020 |
| Short-term effects of the traction-manipulation protocol in dizziness intensity and disability in cervicogenic dizziness: a randomized controlled trial [with consumer summary] | Carrasco-Uribarren A; Rodriguez-Sanz J; Lopez-de-Celis C; Perez-Guillen S; Tricas-Moreno JM; Cabanillas-Barea S | 2022 |
| The effects of osteopathic manipulative treatment on pain and disability in patients with chronic neck pain: a single-blinded randomized controlled trial | Cholewicki J; Popovich JM; Reeves NP; de Stefano LA; Rowan JJ; Francisco TJ; Prokop LL; Zatkin MA; Lee AS; Sikorskii A; Pathak PK; Choi J; Radcliffe CJ; Ramadan A | 2021 |
| Massage manipulation versus low back muscle exercise for lumbar intervertebral instability: a preliminary randomized clinical trial | Zhou Z; Zhang Y; Chen W; Wang J | 2020 |
| Effects of low amplitude high velocity thurst manipulation as compare to grade III maitland mobilization of thoracic spine on mechanical neck pain and disability | Perveen S.; Zahra S.; Mahmood T.; Haider R.; Ayub A. | 2020 |
| Comparing manual and mechanically assisted manipulations of the thoracic spine in neck pain patients: A pilot study | Langenfeld A.; Humphreys B.K.; De Bie R.A.; Swanenburg J. | 2018 |
| Comparison of two adjustive indicators in patients with acute low back pain. Chiropractic Technique | Gemmell HA | 1998 |
| Comparison of the Effects of Cervical Thrust Manipulation and Exercise in Mechanical Neck Pain: A Randomized Controlled Trial | Akgüller, Tuğba; Coşkun, Reşat; Analay Akbaba, Yıldız | 2023 |
| Comparative short-term effects of two thoracic spinal manipulation techniques in subjects with chronic mechanical neck pain: A randomized controlled trial | Casanova-Méndez, Amaloha; Oliva-Pascual-Vaca, Ángel; Rodríguez-Blanco, Cleofás; Heredia-Rizo, Alberto Marcos; Gogorza-Arroitaonandia, Kristobal; Almazán-Campos, Ginés | 2014 |
| The effect of spinal manipulative therapy (SMT) on pain reduction and range of motion in patients with acute unilateral neck pain: a pilot study | Pikula, John R. | 1999 |
| A preliminary study comparing the use of cervical/upper thoracic mobilization and manipulation for individuals with mechanical neck pain | Griswold, David; Learman, Ken; O'Halloran, Bryan; Cleland, Josh | 2015 |
| The effect of two manipulative therapy techniques and their outcome in patients with sacroiliac joint syndrome | Kamali, Fahimeh; Shokri, Esmaeil | 2012 |
| Treatment of the sacroiliac joint in patients with leg pain: a randomized-controlled trial | Visser, L. H.; Woudenberg, N. P.; Bont, J. de; Eijs, F. van; Verwer, K.; Jenniskens, H.; Oudsten, B. L. den | 2013 |
| Effects of spinal manipulation or mobilization as an adjunct to neurodynamic mobilization for lumbar disc herniation with radiculopathy: a randomized clinical trial | Danazumi, Musa Sani; Nuhu, Jibril Mohammed; Ibrahim, Shehu Usman; Falke, Mubarak Abubakar; Rufai, Salim Abubakar; Abdu, Usman Garba; Adamu, Isa Abubakar; Usman, Musbahu Hamisu; Daniel Frederic, Abah; Yakasai, Abdulsalam Mohammed | 2023 |
| Spinal manipulative therapy and exercise for seniors with chronic neck pain | Maiers, Michele; Bronfort, Gert; Evans, Roni; Hartvigsen, Jan; Svendsen, Kenneth; Bracha, Yiscah; Schulz, Craig; Schulz, Karen; Grimm, Richard | 2014 |
| The Effects of Spinal Manipulation Added to Exercise on Pain and Quality of Life in Patients with Thoracic Spinal Pain: A Randomized Controlled Trial | Waqas, Muhammad Sharif; Karimi, Hossein; Ahmad, Ashfaq; Rafiq, Shazia; Anwar, Naveed; Liaqat, Sidrah | 2023 |
| Comparison of two different manual techniques for an exercise program for the management of chronic neck pain: a randomized clinical trial study | Lytras D; Sykaras E; Iakovidis P; Kasimis K; Kottaras A; Mouratidou C | 2023 |
| Comparative effects of Mulligan's mobilization, spinal manipulation, and conventional massage therapy in cervicogenic headache-a prospective, randomized, controlled trial | Nambi G; Alghadier M; Ebrahim EE; Vellaiyan A; Tedla JS; Reddy RS; Kakaraparthi VN; Aldhafian OR; Alshahrani NN; Saleh AK | 2022 |
| A single-center study comparing the effects of thoracic spine manipulation versus mobility exercises in 26 office workers with chronic neck pain: a randomized controlled clinical study | Seo J; Song C; Shin D | 2022 |
| Immediate effects of a single session of cervical spine manipulation on cervical movement patterns in people with nonspecific neck pain: A randomized controlled trial. | Serra-Añó P; Venegas W; Page A | 2023 |
| Immediate systemic neuroimmune responses following spinal mobilisation and manipulation in people with non-specific neck pain: a randomised placebo-controlled trial | Lutke Schipholt, IJ; Coppieters, MW; Reijm, M; Bontkes, HJ; Scholten-Peeters, GGM | 2023 |
| The effects of osteopathic manipulative treatment on pain and disability in patients with chronic low back pain: a single-blinded randomized controlled trial. | Popovich, John M Jr.; ; Cholewicki, Jacek; Reeves, N Peter; DeStefano, Lisa A; Rowan, Jacob J; Francisco, Timothy J; Prokop, Lawrence L; Zatkin, Mathew A; Lee, Angela S; Sikorskii, Alla; Pathak, Pramod K; Choi, Jongeun; Radcliffe, Clark J; Ramadan, Ahmed | 2024 |
| Effect of thoracic manipulation on neck pain in the mobility group: A randomized controlled trial. | Yoshida, Ryota; ; Ichikawa, Kazuna; Nagahori, Hiraku; Tazawa, Tomohiro; Kuruma, Hironobu | 2024 |
| Effects of osteopathic manipulative treatment associated with transcranial direct current stimulation in individuals with chronic low back pain: A double-blind, randomised placebo-controlled trial. | Armbrust, Danilo; ; Areas, Guilherme Peixoto Tinoco; Fonseca, Carlos Luques; Areas, Fernando Zanela da Silva; Duarte, Natalia de Almeida Carvalho; Santana, Silvia Ataide Alves; Dumont, Arislander Jonathan Lopes; Neto, Hugo Pasin; Oliveira, Claudia Santos | 2024 |
| Clinical relevance of combined treatment with exercise in patients with chronic low back pain: a randomized controlled trial. | Blanco-Gimenez, P; ; Vicente-Mampel, J; Gargallo, P; Baraja-Vegas, L; Bautista, I J; Ros-Bernal, F; Barrios, C | 2024 |
| Effect of exercise and manual therapy or kinesiotaping on sEMG and pain perception in chronic low back pain: a randomized trial. | Blanco-Gimenez, P; ; Vicente-Mampel, J; Gargallo, P; Maroto-Izquierdo, S; Martin-Ruiz, J; Jaenada-Carrilero, E; Barrios, C | 2024 |
| Dry Needling Versus Manual Therapy for Patients With Mechanical Neck Pain: A Randomized Controlled Trial. | Pandya, Jeevan; ; Puentedura, Emilio J; Koppenhaver, Shane; Cleland, Josh | 2024 |
| One spinal manipulation session reduces local pain sensitivity but does not affect postural stability in individuals with chronic low back pain: a randomised, placebo-controlled trial. | Freitas, Joao Paulo; ; Correa, Leticia Amaral; Bittencourt, Juliana Valentim; Armstrong, Karine Marcondes; Meziat-Filho, Ney; Nogueira, Leandro Alberto Calazans | 2024 |
| Comparison of Short-Term Effects of Different Spinal Manipulations in Patients with Chronic Non-Specific Neck Pain: A Randomized Controlled Trial. | Garcia-Gonzalez, Jessica; ; Romero-Del Rey, Raul; Martinez-Martin, Virginia; Requena-Mullor, Mar; Alarcon-Rodriguez, Raquel | 2024 |
| Assessment of Long-term Effects of Adding Osteopathic Manipulative Treatment to Neck Exercises for Individuals With Non-specific Chronic Neck Pain: A Randomized Trial. | Groisman, Sandro; ; da Silva, Luciano de Souza; Sanches, Tamara Rocha Ribeiro; Rocha, Clarice Sperotto Dos Santos; Malysz, Tais; Jotz, Geraldo Pereira | 2023 |
| Spinal manipulation and electrical dry needling as an adjunct to conventional physical therapy in patients with lumbar spinal stenosis: a multi-center randomized clinical trial | Young, I; Dunning, J; Butts, R; Bliton, P; Zacharko, N; Garcia, J; Mourad, F; Charlebois, C; Gorby, P; Fernandez-de-Las-Penas, C | 2024 |
| Comparative effectiveness of cervical versus thoracic spinal-thrust manipulation for care of cervicogenic headache: a randomized controlled trial | Nambi G; Alghadier M; Eltayeb MM; Aldhafian OR; Saleh AK; Alsanousi N; Albarakati AJA; Omar MA; Ibrahim MNA; Attallah AA; Ismail MA; Elfeshawy M | 2024 |
| Spinal Manipulation and Clinician-Supported Biopsychosocial Self-Management for Acute Back Pain: The PACBACK Randomized Clinical Trial | Bronfort G.; Meier E.N.; Leininger B.; Schneider M.; Evans R.; Greco C.; Hanson L.; McFarland C.; Chou R.; Connett J.; Delitto A.; George S.Z.; Glick R.M.; Keefe F.; Licciardone J.; Schulz C.; Turk D.; Heagerty P.J. | 2026 |
| Osteopathic manipulative treatment, pain neuroscience education and clinical hypnosis as pain management interventions in chronic low back pain: a randomized sham-controlled feasibility pilot trial | Luchesi G.L.S.; da Silva A.K.F.; Chaves T.C.; Jassi F.J. | 2026 |
| Acute Impact of Spinal Manipulation on Pain and Muscle Mechanical Properties in Chronic Low Back Pain: An Assessor Blinded, Randomized Controlled Trial | Araci A.; Eda Kurt E.; Tas S. | 2025 |
| Comfort during side posture lumbopelvic manipulation in a low back pain population-effects of a typical versus modified flexed lumbopelvic position: a crossover randomized control trial | Wang S.; Frangakis E.M.; Funabashi M.; Hogg-Johnson S. | 2026 |
| Efficacy and safety of Shi's cervical manipulation versus diclofenac for patients with acute and subacute neck pain: a multicenter clinical randomized trial | Zhang M.; Lin X.; Guoqing D.U.; Liu C.; Yuan W.; Wei L.I.; Yang J.; Chen B.; Xiaoyue Y.U.; Xiong Y.; Jiang E.; Gao N.; Jiang S.; Zhenqiu X.U.; Wang X.; Zhan H.; Yufeng W.U. | 2025 |
| The impact of spinal manipulation on lumbar proprioception and its link to pain relief: a randomized controlled trial | Nyiro L.; Dorig M.; Suter M.; Connolly L.; Vogel N.; Stadler C.; John-Cecere G.; Schweinhardt P.; Meier M.L. | 2025 |
| Cervical Manipulation Promotes Rapid Recovery of Neck Pain and Stiffness, Further Facilitates Correction of Cervical Alignment in Patients with Mechanical Neck Pain | Wu K.; Deng R.; Zhao X.; Wang Z.; Ru Q.; Chen X.; Zhang B.; Kang R.; Chen L. | 2025 |
| Effects of instrument-assisted manipulation and mobilization with movement in chronic non-specific low back pain: A randomized controlled trial | Erol E.; Burak M.; Elbasan B. | 2025 |
| Effect of osteopathic manipulative treatment on pain, flexibility, autonomic modulation of heart rate, energy and thermal profile in patients with chronic low back pain. blind randomized clinical trial | Hartz C.S.; Almeida de Molon Mendes M.; Buck K.H.; Moreno M.A.; Bortolazzo G.L. | 2025 |
| Osteopathic diagnosis and treatment of the spine in patients with chronic back pain through the lens of medical infrared thermography: A randomized controlled pilot study | Bohlen L.; Biester A.; Rapp O.; Wentzel J.; Liem T.; Cerritelli F.; Schmidt T. | 2025 |
| The EMU manipulation study: A randomized trial investigating the efficacy of matched or unmatched cervical/thoracic manipulations on neck pain | Swanson B.T.; Cimen M.; DiMercurio C.D.; Dunne A.G.; Dunne M.S.; Hubbard C.; Kirck B.C.; Nadeau R.M.; Artzer P. | 2025 |
| The Immediate Hypoalgesic Effects of Mobilization and Manipulation in Patients with Non-Specific Chronic Low Back Pain: A Cross-Over Randomized Controlled Trial. | Sampsonis, Thomas; Karanasios, Stefanos; Gioftsos, George | 2025 |
| The Effects of Cervical Manipulation Compared with a Conventional Physiotherapy Program for Patients with Acute Whiplash Injury: a Randomized Controlled Trial | Parera-Turull, J; Garolera, M; Navarro, JB; Bech-Decareda, DE; Gual-Beltran, J; Toledo-Marhuenda, JV; Poveda-Pagan, EJ | 2025 |
| Comparison of general exercises, motor control exercises and spinal manipulation in chronic low back pain patients | Khalid A; Anwar N; Tauqeer S; Khalid K; Zakir A; Munir S | 2024 |

**Excluded Studies**

| Title | Authors | Year | Notes |
| --- | --- | --- | --- |
| Neuromechanical responses to spinal manipulation and mobilization: A crossover randomized clinical trial. | Lardon A; Pagé I; Nougarou F; Descarreaux M | 2022 | Exclusion reason: Wrong outcomes |
| The effect of spinal manipulative therapy and home stretching exercises on heart rate variability in patients with persistent or recurrent neck pain: A randomized controlled trial. | Bakken AG; Eklund A; Hallman DM; Axén I | 2021 | Exclusion reason: Wrong outcomes |
| Naprapathy versus orthopaedic standard care for common musculoskeletal disorders: An 8-year follow-up of a pragmatic randomized controlled trial in Sweden. | Lilje S; Eklund A; Wykman A; Sundberg T; Skillgate E | 2021 | Exclusion reason: Secondary analysis of primary data |
| Approaches to cervical spine mobilization for neck pain: A pilot randomized controlled trial. | Lagoutaris C; Sullivan J; Hancock M; Leaver AM | 2020 | Exclusion reason: Non-SMT intervention |
| Comparative effectiveness of 2 manual therapy techniques in the management of lumbar radiculopathy: A randomized clinical trial. | Bello B; Danazumi MS; Kaka B | 2019 | Exclusion reason: Non-SMT intervention |
| Active visceral manipulation associated with conventional physiotherapy in people with chronic low back pain and visceral dysfunction: A preliminary, randomized, controlled, double-blind clinical trial. | Santos LV; Córdoba LL; Lopes JBP; Oliveira CS; Grecco LAC; Andrade ACBN; Neto HP | 2019 | Exclusion reason: Non-SMT intervention |
| Effect of cervical vs. thoracic spinal manipulation on peripheral neural features and grip strength in subjects with chronic mechanical neck pain: a randomized controlled trial | Bautista-Aguirre, Francisco; Oliva-Pascual-Vaca, Ángel; Heredia-Rizo, Alberto M.; Boscá-Gandía, Juan J.; Ricard, François; Rodriguez-Blanco, Cleofás | 2017 | Exclusion reason: Wrong outcomes |
| Dose-response for chiropractic care of chronic low back pain | Haas, Mitchell; Groupp, Elyse; Kraemer, Dale F. | 2004 | Exclusion reason: Wrong comparator/same intervention |
| Clinical research on lumbar oblique-pulling manipulation in combination with sling exercise therapy for patients with chronic nonspecific low back pain | Wang, Shang-Quan; Chen, Ming; Wei, Xu; Gao, Xin-Xia; Zhao, Guo-Dong | 2019 | Exclusion reason: Insufficient reporting |
| Hypoalgesic effects of three different manual therapy techniques on cervical spine and psychological interaction: A randomized clinical trial | Alonso-Perez, Jose Luis; Lopez-Lopez, Almudena; Touche, Roy La; Lerma-Lara, Sergio; Suarez, Emilio; Rojas, Javier; Bishop, Mark D.; Villafañe, Jorge Hugo; Fernández-Carnero, Josué | 2017 | Exclusion reason: Wrong patient population |
| The effect of thoracic spine mobilization and stabilization exercise on the muscular strength and flexibility of the trunk of chronic low back pain patients | Yang, Seong-Rae; Kim, Kyung; Park, Sun-Ja | 2015 | Exclusion reason: Wrong outcomes |
| Effectiveness of traditional bone setting in treating chronic low back pain: A randomised pilot trial | Zaproudina, N.; Hietikko, T.; Hänninen, O. O. P.; Airaksinen, O. | 2009 | Exclusion reason: Non-SMT intervention |
| The effectiveness of pain neuroscience education combined with manual therapy and home exercise for chronic low back pain: A single-blind randomized controlled trial | Saracoglu, Ismail; Arik, Meltem Isintas; Afsar, Emrah; Gokpinar, Hasan Huseyin | 2022 | Exclusion reason: Non-SMT intervention |
| Efficacy of Manual Therapy versus Conventional Physical Therapy in Chronic Low Back Pain Due to Lumbar Spondylosis. A Pilot Study | Sharma, Arti; Alahmari, Khalid; Ahmed, Irshad | 2015 | Exclusion reason: Non-SMT intervention |
| The effect of manual therapy and exercise in patients with chronic low back pain: Double blind randomized controlled trial | Ulger, Ozlem; Demirel, Aynur; Oz, Müzeyyen; Tamer, Seval | 2017 | Exclusion reason: Not clear if SMT is provided |
| Effects of a Single Session of Posterior-to-Anterior Spinal Mobilization and Press-up Exercise on Pain Response and Lumbar Spine Extension in People With Nonspecific Low Back Pain | Powers, Christopher M; Beneck, George J; Kulig, Kornelia; Landel, Robert F; Fredericson, Michael | 2008 | Exclusion reason: Non-SMT intervention |
| Stabilizing training compared with manual treatment in sub-acute and chronic low-back pain | Rasmussen-Barr, E; Nilsson-Wikmar, L; Arvidsson, I | 2003 | Exclusion reason: Non-SMT intervention |
| A Randomized Trial Investigating a Chiropractic Manual Placebo: A Novel Design Using Standardized Forces in the Delivery of Active and Control Treatments | Hawk, Cheryl; Long, Cynthia R.; Rowell, Robert M.; Gudavalli, M. Ram; Jedlicka, James | 2005 | Exclusion reason: Non-SMT intervention |
| A Randomized, Single-Blind, Placebo-Controlled Study on the Efficacy of the Arthrokinematic Approach-Hakata Method in Patients with Chronic Nonspecific Low Back Pain | Kogure, Akira; Kotani, Kazuhiko; Katada, Shigehiko; Takagi, Hiroshi; Kamikozuru, Masahiro; Isaji, Takashi; Hakata, Setsuo | 2015 | Exclusion reason: Non-SMT intervention |
| Spinal mobilization vs conventional physiotherapy in the management of chronic low back pain due to spinal disk degeneration: a randomized controlled trial | Krekoukias, Georgios; Gelalis, Ioannis D.; Xenakis, Theodoros; Gioftsos, Georgios; Dimitriadis, Zacharias; Sakellari, Vasiliki | 2017 | Exclusion reason: Non-SMT intervention |
| Effects of traditional Thai massage versus joint mobilization on substance P and pain perception in patients with non-specific low back pain | Mackawan, Surussawadi; Eungpinichpong, Wichi; Pantumethakul, Rungthip; Chatchawan, Uraiwon; Hunsawong, Tokamol; Arayawichanon, Pricha | 2007 | Exclusion reason: Non-SMT intervention |
| Comparative Effectiveness of Chuna Manipulative Therapy for Non-Acute Lower Back Pain: A Multi-Center, Pragmatic, Randomized Controlled Trial | Park, Sun-Young; Hwang, Eui-Hyoung; Cho, Jae-Heung; Kim, Koh-Woon; Ha, In-Hyuk; Kim, Me-riong; Nam, Kibong; Lee, Min ho; Lee, Jun-Hwan; Kim, Namkwen; Shin, Byung-Cheul | 2020 | Exclusion reason: Non-SMT intervention |
| Effects of spinal manipulation on sensorimotor function in low back pain patients – A randomised controlled trial | Goertz, Christine M.; Xia, Ting; Long, Cynthia R.; Vining, Robert D.; Pohlman, Katherine A.; DeVocht, James W.; Gudavalli, Maruti R.; Owens, Edward F.; Meeker, William C.; Wilder, David G. | 2016 | Exclusion reason: Wrong outcomes |
| A Randomized Controlled Trial Investigating the Efficiency of Musculoskeletal Physiotherapy on Chronic Low Back Disorder | Goldby, Lucy Jane; Moore, Ann P.; Doust, Jo; Trew, Marion E. | 2006 | Exclusion reason: Non-SMT intervention |
| Physiotherapy Based on a Biobehavioral Approach with or Without Orthopedic Manual Physical Therapy in the Treatment of Nonspecific Chronic Low Back Pain: A Randomized Controlled Trial | Grande-Alonso, Mónica; Suso-Martí, Luis; Cuenca-Martínez, Ferran; Pardo-Montero, Joaquín; Gil-Martínez, Alfonso; La Touche, Roy | 2019 | Exclusion reason: Non-SMT intervention |
| A randomized controlled trial of the effects of muscle stretching, manual therapy and steroid injections in addition to ‘stay active’ care on health-related quality of life in acute or subacute low back pain | Grunnesjö, Marie I; Bogefeldt, Johan P; Blomberg, Stefan IE; Strender, Lars-Erik; Svärdsudd, Kurt F | 2011 | Exclusion reason: Wrong outcomes |
| Comparison of two mobilization techniques in management of chronic non-specific low back pain | Ali, Md. Nasir; Sethi, Kritika; Noohu, Majumi M. | 2019 | Exclusion reason: Non-SMT intervention |
| Spinal Manipulative Therapy Has an Immediate Effect on Thermal Pain Sensitivity in People With Low Back Pain: A Randomized Controlled Trial | Bialosky, Joel E.; Bishop, Mark D.; Robinson, Michael E.; Zeppieri, Giorgio, Jr; George, Steven Z. | 2009 | Exclusion reason: Wrong outcomes |
| Magnetic Resonance Imaging Zygapophyseal Joint Space Changes (Gapping) in Low Back Pain Patients Following Spinal Manipulation and Side-Posture Positioning: A Randomized Controlled Mechanisms Trial With Blinding | Cramer, Gregory D.; Cambron, Jerrilyn; Cantu, Joe A.; Dexheimer, Jennifer M.; Pocius, Judith D.; Gregerson, Douglas; Fergus, Michael; McKinnis, Ray; Grieve, Thomas J. | 2013 | Exclusion reason: Wrong study design |
| Changes in Cytokines, Sensory Tests, and Self-reported Pain Levels After Manual Treatment of Low Back Pain | Degenhardt, Brian F.; Johnson, Jane C.; Fossum, Christian; Andicochea, Chad T.; Stuart, Melissa K. | 2017 | Exclusion reason: Not clear if SMT is provided |
| The Nordic Maintenance Care program: Effectiveness of chiropractic maintenance care versus symptom-guided treatment for recurrent and persistent low back pain—A pragmatic randomized controlled trial | Eklund, Andreas; Jensen, Irene; Lohela-Karlsson, Malin; Hagberg, Jan; Leboeuf-Yde, Charlotte; Kongsted, Alice; Bodin, Lennart; Axén, Iben | 2018 | Exclusion reason: Wrong comparator/same intervention |
| Effectiveness of spinal mobilization and postural correction exercises in the management of cervicogenic headache: A randomized controlled trial | Rani, Monika; Kaur, Jaspreet | 2022 | Exclusion reason: Non-SMT intervention |
| Efficacy of osteopathic manipulative treatment for low back pain in euhydrated and hypohydrated conditions: a randomized crossover trial | Parker, Justine; Heinking, Kurt P.; Kappler, Robert E. | 2012 | Exclusion reason: Wrong comparator/same intervention |
| Naprapathic Manual Therapy or Evidence-based Care for Back and Neck Pain: A Randomized, Controlled Trial | Skillgate, Eva; Vingård, Eva; Alfredsson, Lars | 2007 | Exclusion reason: Not clear if SMT is provided |
| A Comparison of the Effects of Stabilization Exercises Plus Manual Therapy to Those of Stabilization Exercises Alone in Patients With Nonspecific Mechanical Neck Pain: A Randomized Clinical Trial | Celenay, Seyda Toprak; Akbayrak, Turkan; Kaya, Derya Ozer | 2016 | Exclusion reason: Non-SMT intervention |
| Effectiveness of Dry Needling, Manual Therapy, and Kinesio Taping® for Patients with Chronic Myofascial Neck Pain: A Single-Blind Clinical Trial | Sobhani, Vahid; Shamsoddini, Alireza; Khatibi-Aghda, Amideddin; Mazloum, Vahid; Hesari Kia, Hamid; Emami Meybod, Mohammad Kazem | 2017 | Exclusion reason: Non-SMT intervention |
| Effectiveness and Cost-Effectiveness of Tuina for Chronic Neck Pain: A Randomized Controlled Trial Comparing Tuina with a No-Intervention Waiting List | Pach, Daniel; Piper, Mike; Lotz, Fabian; Reinhold, Thomas; Dombrowski, Mirja; Chang, Yinghui; Liu, Bin; Blödt, Susanne; Rotter, Gabriele; Icke, Katja; Witt, Claudia M. | 2018 | Exclusion reason: Non-SMT intervention |
| Stretching as an adjunct to chiropractic manipulation of chronic neck pain -- before, after or not at all? A prospective randomized controlled clinical trial | Allan M, Brantingham JW, Menezes A |  | Exclusion reason: Full text not accessible |
| Effect of manual therapy and stretching on neck muscle strength and mobility in chronic neck pain | Häkkinen, Arja; Salo, Petri; Tarvainen, Ulla; Wirén, Kaija; Ylinen, Jari | 2007 | Exclusion reason: Non-SMT intervention |
| Immediate effects of atlanto-occipital joint manipulation on active mouth opening and pressure pain sensitivity in women with mechanical neck pain | Mansilla-Ferragut, Pilar; Fernández-de-Las Peñas, César; Alburquerque-Sendín, Francisco; Cleland, Joshua A.; Boscá-Gandía, Juan José | 2009 | Exclusion reason: Wrong outcomes |
| Manual therapy, physical therapy, or continued care by a general practitioner for patients with neck pain. A randomized, controlled trial | Hoving, Jan Lucas; Koes, Bart W.; de Vet, Henrica C. W.; van der Windt, Danielle A. W. M.; Assendelft, Willem J. J.; van Mameren, Henk; Devillé, Walter L. J. M.; Pool, Jan J. M.; Scholten, Rob J. P. M.; Bouter, Lex M. | 2002 | Exclusion reason: Non-SMT intervention |
| Immediate effects of the central posteroanterior mobilization technique on pain and range of motion in patients with mechanical neck pain | Kanlayanaphotporn, Rotsalai; Chiradejnant, Adit; Vachalathiti, Roongtiwa | 2010 | Exclusion reason: Non-SMT intervention |
| A pilot study for a randomized clinical trial assessing chiropractic care, medical care, and self-care education for acute and subacute neck pain patients | Evans, Roni; Bronfort, Gert; Bittell, Sarah; Anderson, Alfred V. | 2003 | Exclusion reason: Wrong outcomes |
| The addition of cervical thrust manipulations to a manual physical therapy approach in patients treated for mechanical neck pain: a secondary analysis | Boyles, Robert E.; Walker, Michael J.; Young, Brian A.; Strunce, Joseph; Wainner, Robert S. | 2010 | Exclusion reason: Not original research paper |
| Primary care randomized clinical trial: manual therapy effectiveness in comparison with TENS in patients with neck pain | Escortell-Mayor, E.; Riesgo-Fuertes, R.; Garrido-Elustondo, S.; Asúnsolo-Del Barco, A.; Díaz-Pulido, B.; Blanco-Díaz, M.; Bejerano-Álvarez, E.; TEMA-TENS Group | 2011 | Exclusion reason: Non-SMT intervention |
| Stretching exercises vs manual therapy in treatment of chronic neck pain: a randomized, controlled cross-over trial | Ylinen, Jari; Kautiainen, Hannu; Wirén, Kaija; Häkkinen, Arja | 2007 | Exclusion reason: Non-SMT intervention |
| Effectiveness of traditional bone setting in chronic neck pain: randomized clinical trial | Zaproudina, Nina; Hänninen, Osmo O. P.; Airaksinen, Olavi | 2007 | Exclusion reason: Non-SMT intervention |
| Joint mobilization vs massage for chronic mechanical neck pain: a pilot study to assess recruitment strategies and estimate outcome measure variability | Madson, Timothy J.; Cieslak, Kathryn R.; Gay, Ralph E. | 2010 | Exclusion reason: Non-SMT intervention |
| The immediate effects of mobilization technique on pain and range of motion in patients presenting with unilateral neck pain: a randomized controlled trial | Kanlayanaphotporn, Rotsalai; Chiradejnant, Adit; Vachalathiti, Roongtiwa | 2009 | Exclusion reason: Non-SMT intervention |
| Manual Therapy, Therapeutic Patient Education, and Therapeutic Exercise, an Effective Multimodal Treatment of Nonspecific Chronic Neck Pain: A Randomized Controlled Trial | Beltran-Alacreu, Hector; López-de-Uralde-Villanueva, Ibai; Fernández-Carnero, Josué; La Touche, Roy | 2015 | Exclusion reason: Wrong comparator/same intervention |
| Effectiveness of manual therapy or pulsed shortwave diathermy in addition to advice and exercise for neck disorders: a pragmatic randomized controlled trial in physical therapy clinics | Dziedzic, Krysia; Hill, Jonathan; Lewis, Martyn; Sim, Julius; Daniels, Jane; Hay, Elaine M. | 2005 | Exclusion reason: Not clear if SMT is provided |
| Bone setting for prolonged neck pain: a randomized clinical trial | Hemmilä, Heikki M. | 2005 | Exclusion reason: Non-SMT intervention |
| Independent evaluation of a clinical prediction rule for spinal manipulative therapy: a randomised controlled trial | Hancock, Mark J.; Maher, Christopher G.; Latimer, Jane; Herbert, Robert D.; McAuley, James H. | 2008 | Exclusion reason: Secondary analysis of primary data |
| Comparison of Dry Needling versus Orthopedic Manual Therapy in Patients with Myofascial Chronic Neck Pain: A Single-Blind, Randomized Pilot Study | Campa-Moran, Irene; Rey-Gudin, Etelvina; Fernández-Carnero, Josué; Paris-Alemany, Alba; Gil-Martinez, Alfonso; Lerma Lara, Sergio; Prieto-Baquero, Almudena; Alonso-Perez, José Luis; La Touche, Roy | 2015 | Exclusion reason: Non-SMT intervention |
| Effect of Seven Sessions of Posterior-to-Anterior Spinal Mobilisation versus Prone Press-ups in Non-Specific Low Back Pain - Randomized Clinical Trial | Shah, Shlesha G.; Kage, Vijay | 2016 | Exclusion reason: Non-SMT intervention |
| The effectiveness of manual therapy and proprioceptive neuromuscular facilitation compared to kinesiotherapy: a four-arm randomized controlled trial | Zaworski, Kamil; Latosiewicz, Robert | 2021 | Exclusion reason: Non-SMT intervention |
| A randomized clinical trial of manual therapy for cervico-brachial pain syndrome -- a pilot study | Allison, G. T.; Nagy, B. M.; Hall, T. | 2002 | Exclusion reason: Non-SMT intervention |
| A comparison of selected osteopathic treatment and relaxation for tension-type headaches | Anderson, Rosemary E.; Seniscal, Caryn | 2006 | Exclusion reason: Wrong outcomes |
| The efficacy of manual treatment in low back pain: a clinical trial | Arkuszewski, Z. |  | Exclusion reason: Full text not accessible |
| Manual therapy with steroid injections in low-back pain. Improvement of quality of life in a controlled trial with four months' follow-up | Blomberg, S.; Svärdsudd, K.; Tibblin, G. | 1993 | Exclusion reason: Wrong outcomes |
| Manual therapy with steroid injections--a new approach to treatment of low back pain. A controlled multicenter trial with an evaluation by orthopedic surgeons | Blomberg, S.; Hallin, G.; Grann, K.; Berg, E.; Sennerby, U. | 1994 | Exclusion reason: Wrong outcomes |
| Efficacy of high-velocity low-amplitude manipulative technique in subjects with low-back pain during menstrual cramping | Boesler, D.; Warner, M.; Alpers, A.; Finnerty, E. P.; Kilmore, M. A. | 1993 | Exclusion reason: Wrong outcomes |
| Spinal manipulation vs. amitriptyline for the treatment of chronic tension-type headaches: a randomized clinical trial | Boline, P. D.; Kassak, K.; Bronfort, G.; Nelson, C.; Anderson, A. V. | 1995 | Exclusion reason: Wrong patient population |
| Spinal manipulation, epidural injections, and self-care for sciatica: a pilot study for a randomized clinical trial | Bronfort, Gert; Evans, Roni L.; Maiers, Michele; Anderson, Alfred V. | 2004 | Exclusion reason: Wrong outcomes |
| Predictors of functional outcome in patients with chronic low back pain undergoing back school, individual physiotherapy or spinal manipulation | Cecchi, F.; Negrini, S.; Pasquini, G.; Paperini, A.; Conti, A. A.; Chiti, M.; Zaina, F.; Macchi, C.; Molino-Lova, R. | 2012 | Exclusion reason: Secondary analysis of primary data |
| Chiropractic spinal manipulative therapy for cervicogenic headache: a single-blinded, placebo, randomized controlled trial | Chaibi, Aleksander; Knackstedt, Heidi; Tuchin, Peter J.; Russell, Michael Bjørn | 2017 | Exclusion reason: Wrong outcomes |
| A perspective for considering the risks and benefits of spinal manipulation in patients with low back pain | Childs, John D.; Flynn, Timothy W.; Fritz, Julie M. | 2006 | Exclusion reason: Wrong outcomes |
| Multicentre trial of physiotherapy in the management of sciatic symptoms | Coxhead, C. E.; Inskip, H.; Meade, T. W.; North, W. R.; Troup, J. D. | 1981 | Exclusion reason: Wrong outcomes |
| Chronic neck pain: a comparison of acupuncture treatment and physiotherapy | David, J.; Modi, S.; Aluko, A. A.; Robertshaw, C.; Farebrother, J. | 1998 | Exclusion reason: Non-SMT intervention |
| Evaluation of a modified clinical prediction rule for use with spinal manipulative therapy in patients with chronic low back pain: a randomized clinical trial | Dougherty, Paul E.; Karuza, Jurgis; Savino, Dorian; Katz, Paul | 2014 | Exclusion reason: SMT effect not estimable/multimodal |
| Manipulation in treatment of low back pain: a multicentre study | Doran, D. M.; Newell, D. J. | 1975 | Exclusion reason: Not clear if SMT is provided |
| Low back pain treated by manipulation; a controlled series | Coyer, A. B.; Curwen, I. H. | 1955 | Exclusion reason: Wrong study design |
| [Randomized clinical trial for primary care patients with neck pain: manual therapy versus electrical stimulation] | Escortell Mayor, Esperanza; Lebrijo Pérez, Gerardo; Pérez Martín, Yolanda; Asúnsolo del Barco, Angel; Riesgo Fuertes, Rosario; Saa Requejo, Carmen; TEMA-TENS Group | 2008 | Exclusion reason: Non-SMT intervention |
| Lumbar spinal manipulation on trial. Part I--clinical assessment | Evans, D. P.; Burke, M. S.; Lloyd, K. N.; Roberts, E. E.; Roberts, G. M. | 1978 | Exclusion reason: Wrong outcomes |
| Changes in recruitment of transversus abdominis correlate with disability in people with chronic low back pain | Ferreira, P. H.; Ferreira, M. L.; Maher, C. G.; Refshauge, K.; Herbert, R. D.; Hodges, P. W. | 2010 | Exclusion reason: Non-SMT intervention |
| A controlled trial of manipulation in a selected group of patients with low back pain favouring one side | Fisk, J. W. | 1979 | Exclusion reason: Wrong study design |
| A randomized, controlled trial of manual therapy and specific adjuvant exercise for chronic low back pain | Geisser, Michael E.; Wiggert, Elizabeth A.; Haig, Andrew J.; Colwell, Miles O. | 2005 | Exclusion reason: Non-SMT intervention |
| Controlled comparison of short-wave diathermy treatment with osteopathic treatment in non-specific low back pain | Gibson, T.; Grahame, R.; Harkness, J.; Woo, P.; Blagrave, P.; Hills, R. | 1985 | Exclusion reason: Not clear if SMT is provided |
| A clinical trial of rotational manipulation of the spine in back pain cases occurring in a factory | Glover, J. R. | 1966 | Exclusion reason: Protocol paper |
| A randomized trial of manipulation for low-back pain in a medical setting | Godfrey, C. M.; Morgan, P. P.; Schatzker, J. | 1984 | Exclusion reason: Wrong outcomes |
| Short-term effects of lumbar posteroanterior mobilization in individuals with low-back pain | Goodsell, M.; Lee, M.; Latimer, J. | 2000 | Exclusion reason: Non-SMT intervention |
| Does folk medicine work? A randomized clinical trial on patients with prolonged back pain | Hemmilä, H. M.; Keinänen-Kiukaanniemi, S. M.; Levoska, S.; Puska, P. | 1997 | Exclusion reason: Non-SMT intervention |
| Long-term effectiveness of bone-setting, light exercise therapy, and physiotherapy for prolonged back pain: a randomized controlled trial | Hemmilä, Heikki M.; Keinänen-Kiukaanniemi, Sirkka M.; Levoska, Sinikka; Puska, Pekka | 2002 | Exclusion reason: Non-SMT intervention |
| Pregnancy Research on Osteopathic Manipulation Optimizing Treatment Effects: the PROMOTE study | Hensel, Kendi L.; Buchanan, Steve; Brown, Sarah K.; Rodriguez, Mayra; Cruser, des Anges | 2015 | Exclusion reason: Wrong patient population |
| Spinal manipulation for low back pain | Hoehler, F. K.; Tobis, J. S.; Buerger, A. A. | 1981 | Exclusion reason: Wrong outcomes |
| Manual therapy, physical therapy, or continued care by the general practitioner for patients with neck pain: long-term results from a pragmatic randomized clinical trial | Hoving, Jan L.; de Vet, Henrica C. W.; Koes, Bart W.; Mameren, Henk van; Devillé, Walter L. J. M.; van der Windt, Daniëlle A. W. M.; Assendelft, Willem J. J.; Pool, Jan J. M.; Scholten, Rob J. P. M.; Korthals-de Bos, Ingeborg B. C.; Bouter, Lex M. | 2006 | Exclusion reason: Non-SMT intervention |
| Manipulation of the cervical spine--a pilot study | Howe, D. H.; Newcombe, R. G.; Wade, M. T. | 1983 | Exclusion reason: Wrong outcomes |
| Osteopathic manipulation in the treatment of muscle-contraction headache | Hoyt, W. H.; Shaffer, F.; Bard, D. A.; Benesler, J. S.; Blankenhorn, G. D.; Gray, J. H.; Hartman, W. T.; Hughes, L. C. | 1979 | Exclusion reason: Lack of info |
| Adverse reactions to chiropractic treatment and their effects on satisfaction and clinical outcomes among patients enrolled in the UCLA Neck Pain Study | Hurwitz, Eric L.; Morgenstern, Hal; Vassilaki, Maria; Chiang, Lu-May | 2004 | Exclusion reason: Secondary analysis of primary data |
| A randomized trial of chiropractic and medical care for patients with low back pain: eighteen-month follow-up outcomes from the UCLA low back pain study | Hurwitz, Eric L.; Morgenstern, Hal; Kominski, Gerald F.; Yu, Fei; Chiang, Lu-May | 2006 | Exclusion reason: Secondary analysis of primary data |
| Mobilization and manipulation for low-back pain | Jayson, M. I.; Sims-Williams, H.; Young, S.; Baddeley, H.; Collins, E. | 1981 | Exclusion reason: Wrong outcomes |
| Efficacy of lumbar mobilization on postpartum low back pain in Egyptian females: A randomized control trial | Kamel, Dalia M.; Raoof, Neveen A. Abdel; Tantawy, Sayed A. | 2016 | Exclusion reason: Non-SMT intervention |
| Postural and symptomatic improvement after physiotherapy in patients with dizziness of suspected cervical origin | Karlberg, M.; Magnusson, M.; Malmström, E. M.; Melander, A.; Moritz, U. | 1996 | Exclusion reason: Non-SMT intervention |
| Early individualised manipulative rehabilitation following lumbar open laser microdiscectomy improves early post-operative functional disability: A randomized, controlled pilot study | Kim, Byungho J.; Ahn, Junghoon; Cho, Heecheol; Kim, Dongyun; Kim, Taeyeong; Yoon, Bumchul | 2016 | Exclusion reason: Not clear if SMT is provided |
| Manipulative rehabilitation applied soon after lumbar disc surgery improves late post-operative functional disability: A preliminary 2-year follow-up study | Kim, Byungho J.; Kim, Taeyeong; Ahn, Junghoon; Cho, Heecheol; Kim, Dongyun; Yoon, Bumchul | 2017 | Exclusion reason: Non-SMT intervention |
| Strain-counterstrain to treat restrictions of the mobility of the cervical spine in patients with neck pain: a sham-controlled randomized trial | Klein, Reinhold; Bareis, Alexa; Schneider, Antonius; Linde, Klaus | 2013 | Exclusion reason: Non-SMT intervention |
| [Cervicobrachialgia. A controlled trial with conventional therapy and manipulation] | Kogstad, O. A.; Karterud, S.; Gudmundsen, J. | 1978 | Exclusion reason: Wrong outcomes |
| Cost effectiveness of physiotherapy, manual therapy, and general practitioner care for neck pain: economic evaluation alongside a randomised controlled trial | Korthals-de Bos, Ingeborg B. C.; Hoving, Jan L.; van Tulder, Maurits W.; Rutten-van Mölken, Maureen P. M. H.; Adèr, Herman J.; de Vet, Henrica C. W.; Koes, Bart W.; Vondeling, Hindrik; Bouter, Lex M. | 2003 | Exclusion reason: Non-SMT intervention |
| The immediate effects of upper thoracic translatoric spinal manipulation on cervical pain and range of motion: a randomized clinical trial | Krauss, John; Creighton, Doug; Ely, Jonathan D.; Podlewska-Ely, Joanna | 2008 | Exclusion reason: Wrong outcomes |
| [Comparative study on effects of manipulation treatment and transcutaneous electrical nerve stimulation on patients with cervicogenic headache] | Li, Chen; Zhang, Xiu-ling; Ding, Hong; Tao, Yue-qiang; Zhan, Hong-sheng | 2007 | Exclusion reason: Wrong outcomes |
| Osteopathic manipulative treatment of back pain and related symptoms during pregnancy: a randomized controlled trial | Licciardone, John C.; Buchanan, Steve; Hensel, Kendi L.; King, Hollis H.; Fulda, Kimberly G.; Stoll, Scott T. | 2010 | Exclusion reason: Non-SMT intervention |
| Osteopathic manual treatment in patients with diabetes mellitus and comorbid chronic low back pain: subgroup results from the OSTEOPATHIC Trial | Licciardone, John C.; Kearns, Cathleen M.; Hodge, Lisa M.; Minotti, Dennis E. | 2013 | Exclusion reason: Secondary analysis of primary data |
| Outcomes of osteopathic manual treatment for chronic low back pain according to baseline pain severity: results from the OSTEOPATHIC Trial | Licciardone, John C.; Kearns, Cathleen M.; Minotti, Dennis E. | 2013 | Exclusion reason: Secondary analysis of primary data |
| Treatment of protrusion of lumbar intervertebral disc by pulling and turning manipulations | Liu, J.; Zhang, S. | 2000 | Exclusion reason: SMT intervention not specified |
| An open controlled assessment of osteopathic manipulation in nonspecific low-back pain | MacDonald, R. S.; Bell, C. M. | 1990 | Exclusion reason: Wrong study design |
| Back pain and sciatica: controlled trials of manipulation, traction, sclerosant and epidural injections | Mathews, J. A.; Mills, S. B.; Jenkins, V. M.; Grimes, S. M.; Morkel, M. J.; Mathews, W.; Scott, C. M.; Sittampalam, Y. | 1987 | Exclusion reason: Wrong outcomes |
| Early mobilization of acute whiplash injuries | Mealy, K.; Brennan, H.; Fenelon, G. C. | 1986 | Exclusion reason: Non-SMT intervention |
| A randomized controlled trial of the effect of spinal manipulation in the treatment of cervicogenic headache | Nilsson, N. | 1995 | Exclusion reason: Wrong outcomes |
| Lasting changes in passive range motion after spinal manipulation: a randomized, blind, controlled trial | Nilsson, N.; Christensen, H. W.; Hartvigsen, J. | 1996 | Exclusion reason: Wrong outcomes |
| The effect of spinal manipulation in the treatment of cervicogenic headache | Nilsson, N.; Christensen, H. W.; Hartvigsen, J. | 1997 | Exclusion reason: Wrong outcomes |
| Treatment of acute cervical pain--a comparative group study | Nordemar, Rolf; Thörner, Conny | 1981 | Exclusion reason: Non-SMT intervention |
| Relative therapeutic efficacy of vertebral manipulation and conventional treatment in back pain management | Nwuga, V. C. | 1982 | Exclusion reason: Non-SMT intervention |
| Effects of different treatments on postural performance in patients with cervical root compression. A randomized prospective study assessing the importance of the neck in postural control | Persson, L.; Karlberg, M.; Magnusson, M. | 1996 | Exclusion reason: Non-SMT intervention |
| Cervical radiculopathy: pain, muscle weakness and sensory loss in patients with cervical radiculopathy treated with surgery, physiotherapy or cervical collar. A prospective, controlled study | Persson, L. C.; Moritz, U.; Brandt, L.; Carlsson, C. A. | 1997 | Exclusion reason: Non-SMT intervention |
| Long-lasting cervical radicular pain managed with surgery, physiotherapy, or a cervical collar. A prospective, randomized study | Persson, L. C.; Carlsson, C. A.; Carlsson, J. Y. | 1997 | Exclusion reason: Non-SMT intervention |
| Pain, coping, emotional state and physical function in patients with chronic radicular neck pain. A comparison between patients treated with surgery, physiotherapy or neck collar--a blinded, prospective randomized study | Persson, L. C.; Lilja, A. | 2001 | Exclusion reason: Non-SMT intervention |
| Conservative treatment in patients sick-listed for acute low-back pain: a prospective randomised study with 12 months' follow-up | Seferlis, T.; Németh, G.; Carlsson, A. M.; Gillström, P. | 1998 | Exclusion reason: Not clear if SMT is provided |
| Comparison between the effects of Chuna manipulation therapy and cervical traction treatment on pain in patients with herniated cervical disc: a randomized clinical pilot trial | Shin, Byung-Cheul; Kim, Sang-Don; Lee, Myeong Soo | 2006 | Exclusion reason: Not original research paper |
| Cost and effectiveness analysis of chiropractic and physiotherapy treatment for low back and neck pain. Six-month follow-up | Skargren, E. I.; Oberg, B. E.; Carlsson, P. G.; Gade, M. | 1997 | Exclusion reason: SMT effect not estimable/multimodal |
| One-year follow-up comparison of the cost and effectiveness of chiropractic and physiotherapy as primary management for back pain. Subgroup analysis, recurrence, and additional health care utilization | Skargren, E. I.; Carlsson, P. G.; Oberg, B. E. | 1998 | Exclusion reason: Secondary analysis of primary data |
| The long-term effects of naprapathic manual therapy on back and neck pain - results from a pragmatic randomized controlled trial | Skillgate, Eva; Bohman, Tony; Holm, Lena W.; Vingård, Eva; Alfredsson, Lars | 2010 | Exclusion reason: Not clear if SMT is provided |
| Cervical lateral glide increases nociceptive flexion reflex threshold but not pressure or thermal pain thresholds in chronic whiplash associated disorders: A pilot randomised controlled trial | Sterling, Michele; Pedler, Ashley; Chan, Cliffton; Puglisi, Madonna; Vuvan, Viana; Vicenzino, Bill | 2010 | Exclusion reason: Non-SMT intervention |
| A randomized-control study of active and passive treatments for chronic low back pain following L5 laminectomy | Timm, K. E. | 1994 | Exclusion reason: Non-SMT intervention |
| Musculoskeletal manipulation in the treatment of low back pain | Tobis, J. S.; Hoehler, F. K. | 1983 | Exclusion reason: Wrong outcomes |
| United Kingdom back pain exercise and manipulation (UK BEAM) randomised trial: cost effectiveness of physical treatments for back pain in primary care | UK BEAM Trial Team | 2004 | Exclusion reason: Wrong outcomes |
| Pressure pain threshold evaluation of the effect of spinal manipulation in the treatment of chronic neck pain: a pilot study | Vernon, H. T.; Aker, P.; Burns, S.; Viljakaanen, S.; Short, L. | 1990 | Exclusion reason: Wrong outcomes |
| Randomized osteopathic manipulation study (ROMANS): pragmatic trial for spinal pain in primary care | Williams, Nefyn H.; Wilkinson, Clare; Russell, Ian; Edwards, Rhiannon T.; Hibbs, Richard; Linck, Pat; Muntz, Rachel | 2003 | Exclusion reason: Not clear if SMT is provided |
| Treatment of pelvic joint dysfunction in primary care--a controlled study | Wreje, U.; Nordgren, B.; Aberg, H. | 1992 | Exclusion reason: Non-SMT intervention |
| Mobilization versus massage therapy in the treatment of cervicogenic headache: a clinical study | Youssef, Enas F.; Shanb, Al-Sayed A. | 2013 | Exclusion reason: Non-SMT intervention |
| A controlled trial of rotational manipulation in low back pain | Buerger, A A | 1980 | Exclusion reason: Wrong outcomes |
| Short-term effects of thoracic manipulation on lower trapezius muscle strength | Cleland, , J, Selleck, B, Stowell, T, Browne, L, Alberini, S, St Cyr, H; Caron, T | 2004 | Exclusion reason: Wrong outcomes |
| Manipulation in low back pain | Helliwell, , PS; Cunliffe, G | 1987 | Exclusion reason: Wrong outcomes |
| A randomized clinical trial comparing chiropractic To conservative medical care for subacute low back pain | Hoiriis, K | 2001 | Exclusion reason: Conference abstract |
| Rehabilitation with osteopathic manipulative treatment after lumbar disc surgery: a randomised, controlled pilot study | Kim, , BJ, Ahn, J, Cho, H, Kim, D, Kim, T; Yoon, B | 2015 | Exclusion reason: Non-SMT intervention |
| The comparison of the results of manual therapy versus physiotherapy methods used in treatment of patients with low back pain syndromes | Kinalski, , R, Kuwik, W; Pietrzak, D | 1989 | Exclusion reason: Wrong outcomes |
| A randomised controlled trial on the effectiveness of osteopathic manipulative treatment of chronic low back pain | Mandara, A.; Fusaro, A.; Musicco, M.; Bado, F. | 2008 | Exclusion reason: Conference abstract |
| Manipulation and traction for lumbago and sciatica: physiotherapeutic techniques used in two controlled trials | Mathews, , W, Morkel, M; Mathews, J | 1988 | Exclusion reason: Non-SMT intervention |
| A clinical trial investigating the effect of two manipulative approaches in the treatment of mechanical neck pain: a pilot study | Parkin-Smith, , GF; Penter, CS | 1998 | Exclusion reason: Full text not accessible |
| Efficacy of various forms of conservative treatment in low back pain. A comparative study | Postacchini F, Facchini M, Palieri P | 1988 | Exclusion reason: Wrong outcomes |
| A randomized trial comparing manual physical therapy to therapeutic exercises, to a combination of therapies, for the treatment of cervical radiculopathy | Ragonese, J | 2009 | Exclusion reason: Not original research paper |
| Manipulation in treatment of low-back pain : a randomized clinical trial | RASMUSSEN, GG. | 1979 | Exclusion reason: Full text not accessible |
| A comparison of spinal manipulation with physical therapy for treatment of patients with mechanical low back pain | Rayegani, M.; Bahrami, M. H.; Farjad, M.; Ahrari, M. N.; Valaie, N. | 2002 | Exclusion reason: Not includable language |
| Chiropractic adjustments: results of a controlled clinical trial in Egypt | Rupert RL, Wagnon R, Thompson P, Ezzeldin MT | 1985 | Exclusion reason: Full text not accessible |
| Mulligan versus maitland mobilizations in patients with chronic low back dysfunction | Samir, S.M.; Zaky, L.A.; Soliman, M.O. | 2016 | Exclusion reason: Non-SMT intervention |
| Evaluation of the Toftness System of Chiropractic Adjusting for subjects with chronic back pain, chronic tension headaches, or primary dysmenorrhea | Snyder, , BJ; Sanders, GE | 1996 | Exclusion reason: Full text not accessible |
| Preventive Spinal Manipulation for patient with Chronic Neck Pain | Thistle, S | 2011 | Exclusion reason: Not original research paper |
| PAIN, RANGE OF MOTION AND BACK STRENGTH IN CHRONIC MECHANICAL LOW BACK PAIN BEFORE & AFTER LUMBAR MOBILISATION | Verma, Yogita; Goyal, Manu; Narkeesh, Dr |  | Exclusion reason: Non-SMT intervention |
| Associations of cytokine concentrations with key osteopathic lesions and clinical outcomes in patients with nonspecific chronic low back pain: results from the OSTEOPATHIC Trial | Licciardone, John C.; Kearns, Cathleen M.; Hodge, Lisa M.; Bergamini, Michael V. W. | 2012 | Exclusion reason: Secondary analysis of primary data |
| Efficacy of C1-C2 Sustained Natural Apophyseal Glide (SNAG) Versus Posterior Anterior Vertebral Mobilization (PAVMs) in the Management of Cervicogenic Headache | Khan, Muhammad; Ali, Syed Shahzad; Soomro, Rabail Rani | 2014 | Exclusion reason: Non-SMT intervention |
| The effect of spinal manipulation on pain and prostaglandin levels in women with primary dysmenorrhea | Kokjohn, K.; Schmid, D. M.; Triano, J. J.; Brennan, P. C. | 1992 | Exclusion reason: Wrong patient population |
| Effects of cervical manipulation on pain, grip force control, and upper extremity muscle activity: a randomized controlled trial | Bracht, MA; Coan, ACB; Yahya, A; Dos Santos, MJ | 2018 | Exclusion reason: Wrong outcomes |
| Immediate Effects of Upper Cervical Translatoric Mobilization on Cervical Mobility and Pressure Pain Threshold in Patients With Cervicogenic Headache: a Randomized Controlled Trial | Malo-Urriés, M; Tricás-Moreno, JM; Estébanez-de-Miguel, E; Hidalgo-García, C; Carrasco-Uribarren, A; Cabanillas-Barea, S | 2017 | Exclusion reason: Non-SMT intervention |
| Comparison of Cyriax manipulation with traditional physical therapy for the management of cervical discogenic problems. A randomized control trial | Razzaq, A; Sajjad, AG; Yasin, S; Tariq, R; Ashraf, F | 2020 | Exclusion reason: Non-SMT intervention |
| Treating cervical vertigo by rotatiing and tracting manipulation and herbal decoction | Zhao, XW; Bi, FB | 2015 | Exclusion reason: Wrong patient population |
| Comparison of short-term effects of chiropractic cervical manipulation and kinesio taping treatments for patients diagnosed with mechanical neck pain | Aslan, M; Alptekin, HK; Ozden, AV; Vural, M; Suleyman, T; Oncu Alptekin, J | 2021 | Exclusion reason: Full text not accessible |
| The immediate effects of cervical spine manipulation on pain and biochemical markers in females with acute non-specific mechanical neck pain: a randomized clinical trial | Lohman, EB; Pacheco, GR; Gharibvand, L; Daher, N; Devore, K; Bains, G; AlAmeri, M; Berk, LS | 2019 | Exclusion reason: Wrong outcomes |
| Comparison of sub-occipital myofascial release and cervical mobilization in managing cervicogenic headache | Shabbir, M; Rafique, S; Majeed, R; Mahjabeen, H; Waris, M; Hamza, U | 2021 | Exclusion reason: Non-SMT intervention |
| Prospective, Randomized, Double-Blind, Placebo-Controlled Clinical Trial Assessing the Effects of Applying a Force to C5 by a Mechanically Assisted Instrument on Referred Pain to the Shoulder | Hardas, GM; Murrell, GAC | 2018 | Exclusion reason: Wrong patient population |
| Efficacy of cervical spine mobilization versus peripherl nerve slider techniques in cervicobrachial pain syndrome - a randomized clinical trial | Miriam, Marks; T, Schöttker-Königer; A, Probst | 2011 | Exclusion reason: Non-SMT intervention |
| Efficacy of Manual and Manipulative Therapy in the Perception of Pain and Cervical Motion in Patients With Tension-Type Headache: a Randomized, Controlled Clinical Trial | Espí-López, GV; Gómez-Conesa, A | 2014 | Exclusion reason: Wrong patient population |
| To Compare the Immediate Effects of Active Cranio Cervical Flexion Exercise Versus Passive Mobilization of Upper Cervical Spine on Pain, Range of Motion and Cranio Cervical Flexion Test in Patients with Chronic Neck Pain | Kaur, A; Mali, K; Mitra, M | 2018 | Exclusion reason: Non-SMT intervention |
| The impact of spinal manipulation on lower extremity motor control in lumbar spinal stenosis patients: a single- blind randomized clinical trial | Aziz, M; Johnson, M; Passmore, S; Goytan, M; Glazebrook, C | 2016 | Exclusion reason: Conference abstract |
| Rehabilitation effect of exercise with soft tissue manipulation in patients with lumbar muscle strain | Li, H; Zhang, H; Liu, S; Wang, Y; Gai, D; Lu, Q; Gan, H; Shi, Y; Qi, W | 2017 | Exclusion reason: Non-SMT intervention |
| The short-term effects of instrument-based mobilization compared with manual mobilization for low back pain: a randomized clinical trial | Alshami, A; Alqassab, FH | 2022 | Exclusion reason: Non-SMT intervention |
| Immediate Changes After Manual Therapy in Patients With Persistent, Nonspecific Back Pain: a Randomized Controlled Trial | Espí-López, GV; Ruescas-Nicolau, MA; Sanchez-Sanchez, ML; Arnal-Gómez, A; Balasch-Bernat, M; Marques-Sule, E | 2018 | Exclusion reason: Non-SMT intervention |
| Effects of manual therapy, sacroiliac and lumbar exercises in patients with sacroiliac joint dysfunction syndrome | Javadov, A; Ketenci, A | 2018 | Exclusion reason: Conference abstract |
| Clinical observation of chiropractic manipulation in the treatment of degenerative scoliosis | Li, LG; Gao, JH; Gao, CY; Sun, W; Luo, J; Yang, KX; Yu, J; Li, JG; Wang, BJ; Yang, W; et al. | 2022 | Exclusion reason: Non-SMT intervention |
| Dynamic surface electromyography response in nonspecific chronic low back pain treated by spine manipulation - A randomized, placebo-controlled, clinical-trial | Fernandes, WVB; Politti, F; De Cordoba Lanza, F; Lucareli, PRG; Correa, JCF | 2017 | Exclusion reason: Conference abstract |
| Effects of routine physical therapy with and without lumbar mobilization in patients with grade i spondylolisthesis | Khan, U; Fasih, M; Fatima, A; Arif, A; Hanif, K; Abbas, ZU | 2021 | Exclusion reason: Non-SMT intervention |
| Effectiveness of Cervical Spine High-Velocity, Low-Amplitude Thrust Added to Behavioral Education, Soft Tissue Mobilization, and Exercise for People With Temporomandibular Disorder With Myalgia: a Randomized Clinical Trial | Reynolds, B; Puentedura, EJ; Kolber, MJ; Cleland, JA | 2020 | Exclusion reason: Wrong patient population |
| Spinal manipulation and exercise for chronic neck pain: are they more effective when delivered alone or in combination? | Wilson, S | 2001 | Exclusion reason: Not original research paper |
| Non-operative management of lumbar spinal stenosis: a randomized controlled trial comparing a comprehensive vs. a self-directive approach | Ammendolia, C; Cote, P; Southerst, D; Schneider, M; Budgell, B; Bombardier, C; Hawker, G; Rampersaud, YR | 2017 | Exclusion reason: Conference abstract |
| Effect of postero-anterior lumber spine mobilization versus mckenzie press-ups in non-specific low back pain | Ikram, A; Farooq, N; Khan, U; Muhammad, A; Niaz, M; Mehmood, A | 2021 | Exclusion reason: Non-SMT intervention |
| No difference in pressure pain threshold and temporal summation after lumbar spinal manipulation compared to sham: a randomised controlled trial in adults with low back pain | Aspinall, SL; Jacques, A; Leboeuf-Yde, C; Etherington, SJ; Walker, BF | 2019 | Exclusion reason: Wrong outcomes |
| Lumbar Spine Mobilization: measurement and Effects | Mehyar, F | 2017 | Exclusion reason: Not original research paper |
| Backing Down on Pain: osteopathic Manual Treatment Improves Chronic Low Back Pain | Quick, M; Kiefer, D | 2013 | Exclusion reason: Not original research paper |
| To investigate the immediate and long term effects of thoracic manipulation (TM) in patients with chronic mechanical neck pain: a randomized controlled trial | Lau, MH | 2011 | Exclusion reason: Not original research paper |
| Immediate effects of cervical mobilisations on global perceived effect, movement associated pain and neck kinematics in patients with non-specific neck pain. A double blind placebo randomised controlled trial | Lascurain-Aguirrebeña, I; Newham, DJ; Casado-Zumeta, X; Lertxundi, A; Critchley, DJ | 2018 | Exclusion reason: Non-SMT intervention |
| Immediate effects of cervical mobilisations on neck muscle activity during active neck movements in patients with non-specific neck pain. A double blind placebo controlled trial | Lascurain-Aguirrebeña, I; Newham, DJ; Casado-Zumeta, X; Lertxundi, A; Critchley, DJ | 2021 | Exclusion reason: Non-SMT intervention |
| Effects of sling-based manual therapy on cervicothoracic junction in patients with neck pain and forward head posture: a randomized clinical trial | Kim, SY; An, CM; Cha, YS; Kim, DH | 2021 | Exclusion reason: Non-SMT intervention |
| Effect of manual therapy with exercise in patients with chronic cervical radiculopathy: a randomized clinical trial | Alshami, AM; Bamhair, DA | 2021 | Exclusion reason: Non-SMT intervention |
| Upper cervical and upper thoracic spine mobilization versus deep cervical flexors exercise in individuals with forward head posture: a randomized clinical trial investigating their effectiveness | Cho, J; Lee, E; Lee, S | 2019 | Exclusion reason: Non-SMT intervention |
| Efficacy of manual therapy on psychological status and pain in patients with neck pain. A randomized clinical trial | Alansari, SM; Youssef, EF; Shanb, AA | 2021 | Exclusion reason: Non-SMT intervention |
| Does Upper Cervical Manual Therapy Provide Additional Benefit in Disability and Mobility over a Physiotherapy Primary Care Program for Chronic Cervicalgia? A Randomized Controlled Trial | González-Rueda, V; Hidalgo-García, C; Rodríguez-Sanz, J; Bueno-Gracia, E; Pérez-Bellmunt, A; Rodríguez-Rubio, PR; López-de-Celis, C | 2020 | Exclusion reason: Non-SMT intervention |
| Comparison between Manual Traction, Manual Opening technique and Combination in Patients with cervical radiculopathy: randomized Control Trial | Afzal, R; Ghous, M; Shakil Ur Rehman, S; Masood, T | 2019 | Exclusion reason: Non-SMT intervention |
| Effectiveness of manual therapy to the cervical spine with and without manual therapy to the upper thoracic spine in the management of non-specific neck pain; a randomized controlled trial | Arsh, A; Darain, H; Iqbal, M; Rahman, MU; Ullah, I; Khalid, S | 2020 | Exclusion reason: Non-SMT intervention |
| Comparison of an Iranian Traditional Massage (Fateh Method) with Physiotherapy and Acupuncture for Patients with Chronic Low Back Pain: a Randomized Controlled Trial | Namiranian, P; Karimi, M; Razavi, SZE; Garoos, AF; Ayati, MH | 2022 | Exclusion reason: Non-SMT intervention |
| Effects of osteopathic manipulative treatment and bio-electromagnetic energy regulation therapy on lower back pain | Auger, K; Shedlock, G; Coutinho, K; Myers, NE; Lorenzo, S | 2021 | Exclusion reason: Non-SMT intervention |
| Effect of manual approaches with osteopathic modality on brain correlates of interoception: an fMRI study | Cerritelli, F; Chiacchiaretta, P; Gambi, F; Perrucci, MG; Barassi, G; Visciano, C; Bellomo, RG; Saggini, R; Ferretti, A | 2020 | Exclusion reason: Non-SMT intervention |
| Comparison of immediate effects of sling-based manual therapy on specific spine levels in subjects with neck pain and forward head posture: a randomized clinical trial | Kim, DH; Kim, SY | 2020 | Exclusion reason: Non-SMT intervention |
| Randomized controlled trials of needle knife therapy combined with rotation traction manipulation for the treatment of cervical spondylotic radiculopathy | Zhou, ZL; Su, GH; Zheng, BZ; Zuo, YZ; Wei, FL | 2016 | Exclusion reason: Wrong comparator/same intervention |
| The effect of manual therapy and stabilizing exercises on forward head and rounded shoulder postures: a six-week intervention with a one-month follow-up study | Fathollahnejad, K; Letafatkar, A; Hadadnezhad, M | 2019 | Exclusion reason: Non-SMT intervention |
| Comparative Effectiveness and Safety of Concomitant Treatment with Chuna Manual Therapy and Usual Care for Whiplash Injuries: a Multicenter Randomized Controlled Trial | Kim, BJ; Park, AL; Hwang, MS; Heo, I; Park, SY; Cho, JH; Kim, KW; Lee, JH; Ha, IH; Park, KS; et al. | 2022 | Exclusion reason: Not clear if SMT is provided |
| Chuna Manual Therapy vs Usual Care for Patients With Nonspecific Chronic Neck Pain: a Randomized Clinical Trial | Lee, J; Cho, JH; Kim, KW; Lee, JH; Kim, MR; Kim, J; Kim, MY; Cho, HW; Lee, YJ; Lee, SH; et al. | 2021 | Exclusion reason: Non-SMT intervention |
| Acute electromyographic responses of deep thoracic paraspinal muscles to spinal manual therapy interventions. An experimental, randomized cross-over study | Fryer, G; Bird, M; Robbins, B; Johnson, JC | 2017 | Exclusion reason: Wrong outcomes |
| Immediate effects of thoracic manipulation on cervical joint position sense in individuals with mechanical neck pain: a randomized controlled trial | Erdem, EU; Ünver, B; Akbas, E; Kinikli, GI | 2021 | Exclusion reason: Wrong outcomes |
| Sacroiliac joint mobilisation versus transcutaneous electrical nerve stimulation for pregnancy induced posterior pelvic pain-a randomised clinical trial | Vaidya, SM | 2018 | Exclusion reason: Non-SMT intervention |
| Comparison of high velocity low amplitude manipulation and dry needle treatment in patients with nonspecific back pain | Alptekin, HK; Ozden, AV; Alkan, M; Oncu Alptekin, J | 2021 | Exclusion reason: Full text not accessible |
| Clinical outcomes and cost-effectiveness of massage chair therapy versus basic physiotherapy in lower back pain patients: a randomized controlled trial | Kim, SK; Min, A; Jeon, C; Kim, T; Cho, S; Lee, SC; Lee, CK | 2020 | Exclusion reason: Non-SMT intervention |
| Effectiveness of muscle energy technique as compared to Maitland mobilisation for the treatment of chronic sacroiliac joint dysfunction | Zaidi, F; Ahmed, I | 2020 | Exclusion reason: Non-SMT intervention |
| Efficacy of Mulligan mobilisation technique in chronin non-spesific low back pain | Simsek, S; Yagci, N | 2019 | Exclusion reason: Conference abstract |
| Mobilisation with movement versus postero-anterior mobilisation in chronic non specific low back pain | Elrazik, RKA; Samir, SM; Zaki, LA; Koura, GA | 2016 | Exclusion reason: Non-SMT intervention |
| Osteopathic Manual Treatment Compared to Kaltenborn-Evjenth Orthopedic Manual Therapy for Chronic Low Back Pain: a Randomized Study | Lizis, P; Kobza, W; Jaszczur-Nowicki, J; Wisniewski, D | 2021 | Exclusion reason: Protocol paper |
| Immediate effect of a spinal mobilisation intervention on muscle stiffness, tone and elasticity in subjects with lower back pain – A randomized cross-over trial | Hamilton, RI; Garden, CLP; Brown, SJ | 2022 | Exclusion reason: Non-SMT intervention |
| Two types of spinal manipulations and minimal conservative medical care for older adults with subacute or chronic low back pain of a very low level intensity | Rannou, F | 2009 | Exclusion reason: Commentary |
| Physiotherapy rehabilitation for osteoporotic vertebral fracture-a randomised controlled trial and economic evaluation (PROVE trial) | Barker, KL; Newman, M; Stallard, N; Leal, J; Lowe, CM; Javaid, MK; Noufaily, A; Hughes, T; Smith, D; Gandhi, V; et al. | 2020 | Exclusion reason: Non-SMT intervention |
| Immediate effects of spinal manipulation on dynamic electromyographic activity of nonspecific chronic low back pain subjects | Fernandes, WVB; Politti, F; De Cordoba Lanza, F; Lucareli, PG; Correa, JCF | 2017 | Exclusion reason: Conference abstract |
| Short or long-term treatment of spinal disability in older adults with manipulation and exercise: a randomized clinical trial | Maiers, M; Hartvigsen, J; Evans, R; Westrom, K; Wang, Q; Schulz, C; Bronfort, G | 2018 | Exclusion reason: Conference abstract |
| Effect of mobilization time by maitland method in nonspecific low back pain and neck pain | de Aguiar, LES; Oliveira, MRT; Caldas, RR; Correia, MC; Rocha, S; Carneiro, MIS; da Silva Tenório, A; Guerino, MR; Monte-Silva, KK; das Graças Rodrigues de Araújo, M | 2014 | Exclusion reason: Non-SMT intervention |
| The Effect of Mulligan Mobilization Technique in Older Adults with Neck Pain: a Randomized Controlled, Double-Blind Study | Buyukturan, O; Buyukturan, B; Sas, S; Karartı, C; Ceylan, İ | 2018 | Exclusion reason: Non-SMT intervention |
| Comparative effectiveness of proprioceptive neuromuscular facilitation and passive vertebral mobilization for neck disability in patients with mechanical neck pain: a randomized controlled trial | Ashfaq, M; Babur, MN; Malick, WH; Hussain, MA; Awan, WA | 2022 | Exclusion reason: Non-SMT intervention |
| Effects of Joint Mobilization Versus Acupuncture on Pain and Functional Ability in People with Chronic Neck Pain: a Randomized Controlled Trial of Comparative Effectiveness | Voulgarakis, P; Iakovidis, P; Lytras, D; Chatziprodromidou, IP; Kottaras, A; Apostolou, T | 2021 | Exclusion reason: Non-SMT intervention |
| Chiropractic care vs medical care for low back pain: assessment of long-term follow-up data | Cambron, JA | 2005 | Exclusion reason: Non-SMT intervention |
| Quality of Life in aged with low back pain treated with osteopathy | Pedro Odimar dos, Santos; Samaria Ali, Cader; Danielli Braga de, Mello; Marco José Mendonça de, Sousa; Eliana da Silva, Coelho; Estélio Henrique Martin, Dantas | 2011 | Exclusion reason: Full text not accessible |
| Inflammatory response following a short-term course of chiropractic treatment in subjects with and without chronic low back pain | Roy, RA; Boucher, JP; Comtois, AS | 2010 | Exclusion reason: Wrong study design |
| Effect of sacroiliac manipulation on postural sway in quiet standing: a randomized controlled trial | Reza Farazdaghi, M; Motealleh, A; Abtahi, F; Panjan, A; arabon, N; Ghaffarinejad, F | 2018 | Exclusion reason: Wrong outcomes |
| Vertebral manipulations in chronic mechanical low back pain | Mellado, VJ; Reyes, MC; Berral, CJ; Kuschnaroff, M | 2010 | Exclusion reason: Wrong study design |
| Use of osteopathic manipulative treatment for low back pain patients with and without pain medication history | Montrose, S; Vogel, M; Barber, KR | 2021 | Exclusion reason: Wrong study design |
| Fascial manipulation vs. standard physical therapy practice for low back pain diagnoses: a pragmatic study | Harper, B; Steinbeck, L; Aron, A | 2018 | Exclusion reason: Wrong study design |
| No Differences in Outcomes in People with Low Back Pain Who Met the Clinical Prediction Rule for Lumbar Spine Manipulation When a Pragmatic Non-thrust Manipulation Was Used as the Comparator | Learman, K; Showalter, C; O'Halloran, B; Donaldson, M; Cook, C | 2014 | Exclusion reason: Secondary analysis of primary data |
| Spinal manipulation provides better short and long-term reduction in pain and disability for patients with non-specific chronic low back pain | Cecchi, F; Molino-Lova, R; Chiti, M; Pasquini, G; Paperini, A; Conti, AA; MacChi, C | 2010 | Exclusion reason: Not original research paper |
| Chiropractic and self-care for back-related leg pain: design of a randomized clinical trial | Schulz, CA; Hondras, MA; Evans, RL; Gudavalli, MR; Long, CR; Owens, EF; Wilder, DG; Bronfort, G | 2011 | Exclusion reason: Protocol paper |
| Gait kinetic and kinematic changes in chronic low back pain patients and the effect of manual therapy: a randomized controlled trial | Krekoukias, G; Sakellari, V; Anastasiadi, E; Gioftsos, G; Dimitriadis, Z; Soultanis, K; Gelalis, ID | 2021 | Exclusion reason: Non-SMT intervention |
| Osteopathy modulates brain-heart interaction in chronic pain patients: an ASL study | Cerritelli, F; Chiacchiaretta, P; Gambi, F; Saggini, R; Perrucci, MG; Ferretti, A | 2021 | Exclusion reason: Not clear if SMT is provided |
| A randomized trial of combined manipulation, stabilizing exercises, and physsician consultation compared to physician consultation alone for chronic low back pain | Niemisto, L; Lahtinen-Suopanki, T; Rissanen, P; Lindgren, KA; Sarna, S; Hurri, H | 2003 | Exclusion reason: Non-SMT intervention |
| The Changes of Motor Control Strategies in Non-specific Chronic Low Back Pain During Spinal Manipulation and Muscle Energy Techniques: a Beta-band Intermuscular Pair-Wise Coherence Analysis | Ghazi, S; Hadian, MR; Shadmehr, A; Talebian, S; Olyaei, GR; Hajouj, E | 2021 | Exclusion reason: Wrong outcomes |
| Interaction between brain and heart: modification of brain-heart interaction in chronic pain by osteopathic treatment | Cerritelli, F; Chiacchiaretta, P; Gambi, F; Ferretti, A | 2022 | Exclusion reason: Wrong outcomes |
| Therapeutic observation of acupuncture-moxibustion plus MEI Te-lan's manipulations for lumbar intervertebral disc herniation (blood stagnation pattern) | Li, A H; Ai, S C; Zhang, H; Wu, L D; Chen, J; Zhao, Q | 2016 | Exclusion reason: Non-SMT intervention |
| Immediate and short-term effects of upper cervical high-velocity, low-amplitude manipulation on standing postural control and cervical mobility in chronic nonspecific neck pain: a randomized controlled trial | Gomez F; Escriba P; Oliva-Pascual-Vaca J; Mendez-Sanchez R; Puente-Gonzalez AS | 2020 | Exclusion reason: Wrong outcomes |
| Increased voluntary activation of the elbow flexors following a single session of spinal manipulation in a subclinical neck pain population | Kingett M; Holt K; Niazi IK; Nedergaard RW; Lee M; Haavik H | 2019 | Exclusion reason: Wrong patient population |
| Osteopathic manipulative therapy in patients with chronic tension-type headache: a pilot study | Deodato M; Guolo F; Monticco A; Fornari M; Manganotti P; Granato A | 2019 | Exclusion reason: Wrong patient population |
| Effect of osteopathic manipulative treatment versus sham treatment on activity limitations in patients with nonspecific subacute and chronic low back pain: a randomized clinical trial | Nguyen C; Boutron I; Zegarra-Parodi R; Baron G; Alami S; Sanchez K; Daste C; Boisson M; Fabre L; Krief P; Krief G; Lefevre-Colau MM; Rannou F | 2021 | Exclusion reason: Not clear if SMT is provided |
| Effect of spinal manipulation on pain sensitivity, postural sway, and health-related quality of life among patients with non-specific chronic low back pain: a randomised control trial | Sarker KK; Sethi J; Mohanty U | 2019 | Exclusion reason: Wrong outcomes |
| One session of spinal manipulation improves the cardiac autonomic control in patients with musculoskeletal pain: a randomized placebo-controlled trial [with consumer summary] | Rodrigues PTV; Correa LA; Reis FJJ; Meziat-Filho NA; Silva BM; Nogueira LAC | 2021 | Exclusion reason: Wrong outcomes |
| Effectiveness of the muscle energy technique versus osteopathic manipulation in the treatment of sacroiliac joint dysfunction in athletes | Garcia-Penalver UJ; Palop-Montoro MV; Manzano-Sanchez D | 2020 | Exclusion reason: Wrong outcomes |
| (Pelvic-sacral tendon-regulation needling technique of acupuncture combined with manipulative reduction in treatment of postpartum pelvic girdle pain: a randomized controlled trial) [Chinese - simplified characters] | Wu J-M; Zhuo Y-Y; Qin X-L; Yu X-Y; Hu S; Ning Y | 2020 | Exclusion reason: Wrong comparator/same intervention |
| Osteopathic manipulative treatment for chronic low back pain | Licciardone J.C. | 2021 | Exclusion reason: Commentary |
| The effect of osteopathic medicine on pain in musicians with nonspecific chronic neck pain: a randomized controlled trial | Rotter G.; Fernholz I.; Binting S.; Keller T.; Roll S.; Kass B.; Reinhold T.; Willich S.N.; Schmidt A.; Brinkhaus B. | 2020 | Exclusion reason: Not clear if SMT is provided |
| Comparison of two different techniques of mobilization in mechanical neck pain with decrease temporomandibular joint mobility | Iqbal; Rishi P.; Sen S. | 2020 | Exclusion reason: Non-SMT intervention |
| Effectiveness of cervical mobilization and stretching exercise with or without upper thoracic mobilization on non-specific neck pain | Safdar M.; Sarfraz N.; Ishaq I.; Sabir M.; Mahmooda S.; Bashir A. | 2020 | Exclusion reason: Non-SMT intervention |
| The effects of neck mobilization in patients with chronic neck pain: A randomized controlled trial | Farooq M.N.; Mohseni-Bandpei M.A.; Gilani S.A.; Ashfaq M.; Mahmood Q. | 2018 | Exclusion reason: Non-SMT intervention |
| Comparison of the effects of classic massage and mobilization applications on balance in patients with chronic neck pain | Toprak Celenay S.; Ozer Kaya D. | 2018 | Exclusion reason: Conference abstract |
| Immediate Effects of Myofascial Release on the Thoracolumbar Fascia and Osteopathic Treatment for Acute Low Back Pain on Spine Shape Parameters: A Randomized, Placebo-Controlled Trial. | Brandl, Andreas; Egner, Christoph; Schleip, Robert | 2021 | Exclusion reason: Wrong outcomes |
| The effects of manual manipulation therapy on pain and dysfunction in patients with lumbar spinal stenosis. | Oh, Hyunju; Choi, SeokJoo; Lee, Sangyong; Lee, Kwansub; Choi, Jioun | 2020 | Exclusion reason: Non-SMT intervention |
| Is Myofascial Release Therapy Cost-Effective When Compared With Manual Therapy to Treat Workers' Mechanical Neck Pains?. | Rodriguez-Fuentes, Ivan; De Toro, Francisco J; Rodriguez-Fuentes, Gustavo; de Oliveira, Iris M; Meijide-Failde, Rosa; Fuentes-Boquete, Isaac M | 2020 | Exclusion reason: Wrong outcomes |
| [Effect of Tongdu Shujin Decoction () plus Lijin () manipulation on degenerative lumbar spinal stenosis]. | Zheng, Jian-Nan; Peng, Rui; Zhou, Xiao-Hong; Chen, Hong-Bo | 2020 | Exclusion reason: Not includable language |
| Fascial manipulation vs. standard physical therapy practice for low back pain diagnoses: A pragmatic study. | Harper, Brent; Steinbeck, Larry; Aron, Adrian | 2019 | Exclusion reason: Non-SMT intervention |
| Management of persistent coccydynia with transrectal manipulation: results of a combined procedure. | Seker, Ali; Sarikaya, Ilker Abdullah; Korkmaz, Ozgur; Yalcin, Sercan; Malkoc, Melih; Bulbul, Ahmet Murat | 2018 | Exclusion reason: Non-SMT intervention |
| Nonoperative treatments for sciatica: A pilot study for a randomized clinical trial | Bronfort, Gert; Evans, Roni L.; Anderson, Alfred V.; Schellhas, Kurt P.; Garvey, Timothy A.; Marks, Ruth Ann; Bittell, Sarah | 2000 | Exclusion reason: SMT effect not estimable/multimodal |
| Shi-style cervical manipulations for cervical radiculopathy | Cui, Xue-jun; Yao, Min; Ye, Xiu-lan; Wang, Ping; Zhong, Wei-hong; Zhang, Rui-chun; Li, Hui-ying; Hu, Zhi-jun; Tang, Zhan-ying; Wang, Wei-min; Qiao, Wei-ping; Sun, Yue-li; Li, Jun; Gao, Yang; Shi, Qi; Wang, Yongjun | 2017 | Exclusion reason: Non-SMT intervention |
| Effect of High-velocity Low-amplitude Manipulation on Cervical Spine Muscle Strength: A Randomized Clinical Trial | Metcalfe, Stan; Reese, Hilary; Sydenham, Robert | 2006 | Exclusion reason: Wrong outcomes |
| Do participants with low back pain who respond to spinal manipulative therapy differ biomechanically from nonresponders, untreated controls or asymptomatic controls? | An, Howard S.; Wong, Arnold Y. L.; Parent, Eric C.; Dhillon, Sukhvinder S.; Prasad, Narasimha; Kawchuk, Gregory N. | 2015 | Exclusion reason: Wrong study design |
| Osteopathy alleviates pain in chronic non‐specific neck pain: randomised controlled trial | Bischoff, A.; Bischoff, Anne; Nürnberger, Andrea; Nürnberger, A.; Voigt, P.; Schwerla, Florian; Resch, Karl-Ludwig; Resch, K. L. | 2010 | Exclusion reason: Full text not accessible |
| The immediate effects of atlanto-occipital joint manipulation and suboccipital muscle inhibition technique on active mouth opening and pressure pain sensitivity over latent myofascial trigger points in the masticatory muscles. | Oliveira-Campelo, Natalia M.; Rubens-Rebelatto, José; Martín-Vallejo, Francisco J.; Alburquerque-Sendín, Francisco; Fernández-de-las-Peñas, César | 2010 | Exclusion reason: Wrong patient population |
| A systematic review and meta-analysis of randomized controlled trials of manipulative therapy for patients with chronic neck pain. | Liu, Zhen; Shi, Jiao; Huang, Yubo; Zhou, Xingchen; Huang, Huazhi; Wu, Hongjiao; Lv, Lijiang; Lv, Zhizhen | 2023 | Exclusion reason: Wrong study design |
| Effectiveness of spinal manipulation and biopsychosocial self-management compared to medical care for low back pain: a randomized trial study protocol. | Bronfort, Gert; Delitto, Anthony; Schneider, Michael; Heagerty, Patrick J; Chou, Roger; Connett, John; Evans, Roni; George, Steven; Glick, Ronald M; Greco, Carol; Hanson, Linda; Keefe, Francis; Leininger, Brent; Licciardone, John; McFarland, Christine; Meier, Eric; Schulz, Craig; Turk, Dennis | 2023 | Exclusion reason: Protocol paper |
| Investigating the hypoalgesic effects of spinal manipulative therapy using hidden pain conditioning and positive expectation in patients with chronic low back pain: protocol for a randomised controlled trial. | Nogueira Carrer, Helen Cristina; Lima, Thamiris Costa de; George, Steven Z; Reis, Felipe Jose Jandre Dos; Dias, Deise Lilian Costa; Campanha, Bianca Emanuelli Saes; Chaves, Thais Cristina | 2023 | Exclusion reason: Protocol paper |
| Immediate Effects of Adding Dry Needling to Thoracic Manipulation and Exercise in Cervical Range of Motion for Adults With Neck Pain: A Randomized Clinical Trial. | Young, Brian A; Boland, David M; Manzo, Abby; Yaw, Haley; Carlson, Brian; Carrier, Spencer; Corcoran, Kameryn; Dial, Megan; Briggs, Robert B; Tragord, Bradley; Koppenhaver, Shane L | 2022 | Exclusion reason: SMT effect not estimable/multimodal |
| The effect of lumbar spinal manipulation on biomechanical factors and perceived transient pain during prolonged sitting: a laboratory-controlled cross-sectional study. | De Carvalho, D E; Callaghan, J P | 2022 | Exclusion reason: Wrong study design |
| Mechanisms of chiropractic spinal manipulative therapy for patients with chronic primary low back pain: protocol for a mechanistic randomised placebo-controlled trial. | Gevers-Montoro, Carlos; Ortega-De Mues, Arantxa; Piche, Mathieu | 2023 | Exclusion reason: Protocol paper |
| Effectiveness of spinal manipulation in addition to pharmacological treatment in fibromyalgia: a blinded randomized trial | Ince B; Kara M; Erdem I; Yurdakul OV; Erden T; Aydin T | 2023 | Exclusion reason: Wrong patient population |
| Effects of manual versus instrumental spinal manipulation on blood flow of the vertebral and internal carotid arteries in participants with chronic nonspecific neck pain: a single-blind, randomized study [with consumer summary] | Kocabey B; Karagozoglu Coskunsu D; Guven K; Agaoglu MH; Yuce S | 2023 | Exclusion reason: Wrong outcomes |
| Effect of osteopathic manipulation of the sacroiliac joint versus electrotherapy on pain and functional disability in patients with low back pain: a pilot study | Rodriguez-Pastor JA; Caro-Puertolas B; Cana-Pino A; Sanchez-Preciado AM; Garrido-Ardila EM; Apolo-Arenas MD | 2022 | Exclusion reason: Full text not accessible |
| Effectiveness of a Manual Therapy Protocol in Women with Pelvic Pain Due to Endometriosis: a Randomized Clinical Trial | Munoz-Gomez, E; Alcaraz-Martinez, AM; Molla-Casanova, S; Sempere-Rubio, N; Aguilar-Rodriguez, M; Serra-Ano, P; Ingles, M | 2023 | Exclusion reason: Wrong patient population |
| Short-Term Effects of Manual Therapy plus Capacitive and Resistive Electric Transfer Therapy in Individuals with Chronic Non-Specific Low Back Pain: a Randomized Clinical Trial Study | Kasimis, K; Iakovidis, P; Lytras, D; Koutras, G; Chatziprodromidou, IP; Fetlis, A; Ntinou, SR; Keklikoglou, NM; Samiotaki, A; Chasapis, G; et al. | 2023 | Exclusion reason: Not clear if SMT is provided |
| Effects of Maitland’s segmental mobilization and general spine mobilization in patients with cervical spondylosis | Afzal, K; Sajjad, AG; Memon, SI; Tariq, R; Sulaiman, M | 2022 | Exclusion reason: Non-SMT intervention |
| An Additive Effect of Instrument-Assisted Soft Tissue Mobilization with Spinal Manipulation in Cervicogenic Headache: a Randomized Controlled Trial. | Nambi, Gopal; ; Alghadier, Mshari; Khanam, Humaira; Pakkir Mohamed, Shahul Hameed; Aldhafian, Osama R; Alshahrani, Naif A; Mani, Paramasivan; Chevidikunnan, Mohamed Faisal; Khan, Fayaz; Albarakati, Alaa Jameel A | 2024 | Exclusion reason: Wrong comparator/same intervention |
| Immediate effects of thoracic mobilization versus soft tissue release on trunk motion, pain, and lumbar muscle activity in patients with chronic low back pain. | Lin, Cheng-Feng; ; Jankaew, Amornthep; Tsai, Min-Chi; Liao, Jen-Chieh | 2024 | Exclusion reason: Non-SMT intervention |
| Combination of Manual Therapy and Dry Needling Effectively Improves Acute Neck Pain and Muscular Tone and Stiffness in Combat Sports Athletes: A Randomized Controlled Study. | Kuzdzal, Adrian; ; Clemente, Filipe Manuel; Klich, Sebastian; Kawczynski, Adam; Trybulski, Robert | 2024 | Exclusion reason: Non-SMT intervention |
| Proprioceptive neuromuscular facilitation therapy versus manual therapy in patients with neck pain: a randomised controlled trial. | Maicki, Tomasz; ; Trabka, Rafal; Wilk-Franczuk, Magdalena; Krzepkowska, Weronika | 2024 | Exclusion reason: Non-SMT intervention |
| Comparison of the effects of dry needling and spinal manipulative therapy versus spinal manipulative therapy alone on functional disability and endurance in patients with nonspecific chronic low back pain: An experimental study. | Khan, Kashmala; ; Ahmad, Ashfaq; Mohseni Bandpei, Muhammad Ali; Kashif, Muhammad | 2024 | Exclusion reason: Wrong comparator/same intervention |
| Effects of cervicothoracic mobility programme on pain, range of motion and function in patients with chronic back pain. | Naseer, Amna; ; Ur Rehman, Syed Shaki; Fatima, Ghulam; Ikram, Mehwish | 2024 | Exclusion reason: Non-SMT intervention |
| Effects of Manual Therapy Plus Pain Neuroscience Education with Integrated Motivational Interviewing in Individuals with Chronic Non-Specific Low Back Pain: A Randomized Clinical Trial Study. | Kasimis, Konstantinos; ; Apostolou, Thomas; Kallistratos, Ilias; Lytras, Dimitrios; Iakovidis, Paris | 2024 | Exclusion reason: SMT effect not estimable/multimodal |
| Effect of osteopathic manipulative treatment and Bio-Electro-Magnetic Energy Regulation (BEMER) therapy on generalized musculoskeletal neck pain in adults. | Palmer, Genevieve M; ; Dominick, Nicholas; Kane, Melissa; Bawek, Sawyer; Burch, Blake; Sanders, Taylor; Phrathep, Davong; Myers, Nicole; Lorenzo, Santiago | 2024 | Exclusion reason: Non-SMT intervention |
| Comparison of muscle energy technique and facet joint mobilisation in the patient with chronic neck pain: A randomized controlled trial. | Tabassum, Huma; ; Mariam, Misbah; Gondal, Kirran Sikandar | 2024 | Exclusion reason: Non-SMT intervention |
| A randomized controlled trial comparing different sites of high-velocity low amplitude thrust on sensorimotor integration parameters. | Niazi, Imran Khan; ; Navid, Muhammad Samran; Merkle, Christopher; Amjad, Imran; Kumari, Nitika; Trager, Robert J; Holt, Kelly; Haavik, Heidi | 2024 | Exclusion reason: Wrong outcomes |
| [Analysis of clinical efficacy and risk factors of relapse in young patients with cervical instability treated by manipulation]. | Lu, Guang-Qi; ; Zhuang, Ming-Hui; Li, Lu-Guang; Chang, Xiao-Juan; Zhu, Li-Guo; Yu, Jie | 2023 | Exclusion reason: Full text not accessible |
| Additional effect of pain neuroscience education to spinal manipulative therapy on pain and disability for patients with chronic low back pain: a randomized controlled trial. | Tavares, Fernando Augusto Goncalves; ; Rossiter, Joao Vitor Alves; Lima, Gabriela Caroline Leandro; de Oliveira, Leticia Gomes; Cavalcante, Witor Souza; Avila, Mariana Arias; George, Steven Zachary; Chaves, Thais Cristina | 2023 | Exclusion reason: Wrong comparator/same intervention |
| Effect of Maitland mobilization on lumbar proprioception, pain, and disability in patients with chronic nonspecific low back pain | Ibrahim, AM; Elkeblawy, MM; Howeidy, MS; Grase, MO; Aneis, YM | 2023 | Exclusion reason: Non-SMT intervention |
| Effect of Manual Techniques on the cervical and lumbar spine on pain and joint range of motion in individuals with Neck Pain and Nonspecific Low Back Pain | RBR-9zk7qx2, | 2023 | Exclusion reason: Not original research paper |
| The Effect of Manual Therapy on Psychological Factors and Quality of Life in Lumbar Disc Herniation Patients: a Single Blinded Randomized Clinical Trial | Taskaya, B; Taskent, I; Çakilli, M; Yilmaz, Ö | 2024 | Exclusion reason: Non-SMT intervention |
| The Effects of cervical mobilization and clinical pilates exercises in cervicogenic headache: randomized controlled trial | Uzun, M; Ekmekyapar Firat, Y; Ergun, N; Akbayrak, T | 2024 | Exclusion reason: Non-SMT intervention |
| Comparison of the immediate effects of cervical manipulation and foam roller applications in individuals with non-specific neck pain | Tahmaz T; Genc H; Demircioglu G | 2023 | Exclusion reason: Not clear if SMT is provided |
| Effectiveness and safety of manual therapy for inpatients with traffic accident-induced acute neck pain: A randomized controlled trial | Choi S.-W.; Kim K.-H.; Yoon J.-Y.; Lee S.-W.; Park J.-W.; Hong H.-W.; Kyeong D.-H.; Kim M.-K.; Kim S.-N.; Kim C.-Y.; Lee Y.-J.; Lee J.-H.; Kim J.-Y.; Ha I.-H. | 2026 | Exclusion reason: Non-SMT intervention |
| Comparison of Different Manual Therapy Techniques in Patients With Chronic Neck Pain | Toprak Celenay S.; Tanik F.; Ozer Kaya D. | 2025 | Exclusion reason: Non-SMT intervention |
| The effectiveness of thoracic spine manipulation on thoracic spine pain | Takatalo J.; Leinonen T.; Rytkonen M.; Lausmaa M.; Pienimaki T.; Hakkinen A.; Ylinen J. | 2025 | Exclusion reason: Conference abstract |
| Can pre-treatment verbal suggestions influence the short-term effects of spinal manipulation in young adults with chronic non-specific low back pain? A randomized controlled trial | Zaworski K.; Baj-Korpak J.; Tokarska-Rodak M.; Plazuk E.; Dyrda A.; Bialosky J.; Rossettini G. | 2025 | Exclusion reason: Wrong comparator/same intervention |
| One Spinal Manipulation Session Does Not Affect Static, Quasi-Static and Dynamic Balance in Individuals with Chronic Low Back Pain: A Randomized Clinical Controlled Trial | Moreira M.R.G.; Rodrigues H.P.; Suguiura I.T.R.; Souza G.M.T.D.; Genelhu F.D.S.; Ferraz A.P.; Takaki W.S.R.; Lee H.D.; Chung W.F.; Carvalho A.R.D.; Bertolini G.R.F. | 2025 | Exclusion reason: Wrong outcomes |
| Long-term impact of self-mobilization via telerehabilitation vs. manual therapy and home exercise on pain and function in cervical degenerative disease | Aygul G.; Tuncer A.; Ozaltin G.E.; Celik E.B.; Aygul B.I. | 2025 | Exclusion reason: Non-SMT intervention |
| Immersive virtual reality versus cervical mobilization: The effects on joint position sense and pain for chronic neck pain - a randomized controlled trial | Cetin H.; Aydogdu S.; Onal B.; Dulger E.; Bilgin S.; Oge H.K.; Kose N. | 2025 | Exclusion reason: Non-SMT intervention |
| EFFECT OF MOTOR CONTROL EXERCISES WITH SPINAL MANUAL THERAPY ON PAIN, RANGE OF MOTION AND POSTURAL BALANCE IN CHRONIC LOW BACK PAIN | Arun B.; Mohan Gandhi V.; Arumugam M. | 2019 | Exclusion reason: Full text not accessible |
| The Effects of Joint Mobilization and Myofascial Release on Muscle Thickness in Non-Specific Low Back Pain: A Randomized Clinical Trial | Javaid H.M.W.; Rehman S.S.U.; Kashif M.; Bashir M.S.; Zia W. | 2025 | Exclusion reason: Non-SMT intervention |
| Effectiveness and cost-effectiveness of chiropractic and physiotherapy for chronic low back pain: a multicenter RCT in Sweden | Gedin F.; Skeppholm M.; Sparring V.; Zethraeus N. | 2025 | Exclusion reason: Not clear if SMT is provided |
| Effects of manual therapy combined with therapeutic exercise on brain structure in patients with chronic nonspecific neck pain: A randomized controlled trial | Chaikla R.; Sremakaew M.; Saekho S.; Kothan S.; Uthaikhup S. | 2025 | Exclusion reason: Non-SMT intervention |
| COLLABORATIVE CARE FOR OLDER ADULTS WITH BACK PAIN (COCOA): RESULTS OF A PILOT RANDOMIZED CONTROLLED TRIAL | Goertz C.; Salsbury S.; Vining R.; Long C.; Hondras M.; Jones M.; Andresen A.; Killinger L.; Wolinsky F.; Wallace R. | 2016 | Exclusion reason: Conference abstract |
| Osteopathic manipulative treatment vs. standard therapy in the management of acute neck and low back pain in the emergency department. | Hochman, Steven M; Vlasica, Katherine; LaPietra, Alexis; Patel, Bhargavi K; Ju, Christine; Mota, Nicholas J; Wilder, Scott | 2026 | Exclusion reason: Non-SMT intervention |
| Chronic pain outcomes among patients treated by osteopathic vs. allopathic physicians: a 36-month follow-up study. | Licciardone, John C; Lewis, Hanna; Dahl, Kaylee; Adams, Branden; Aryal, Subhash | 2026 | Exclusion reason: Wrong study design |
| Effects of combining manual therapies, neck muscle exercises, and therapeutic education pain neuroscience in patients with migraine: a 3-armed randomized clinical trial. | Tolentino, Gabriella de Almeida; Florencio, Lidiane Lima; Pradela, Juliana; Pinheiro-Araujo, Carina Ferreira; Martins, Jaqueline; de Cassia Cabral Norato, Adrila; Sambini, Felipe Daniel; Malosti Teodoro Rodrigues, Jose Lucas; Fernandez-de-Las-Penas, Cesar; Dach, Fabiola; Bevilaqua-Grossi, Debora | 2025 | Exclusion reason: Wrong patient population |
| Manual therapy and neck-specific exercise are equally effective for treating non-specific neck pain but only when exercise adherence is maximised: A randomised controlled trial. | Villanueva-Ruiz, Iker; Falla, Deborah; Saez, Marc; Araolaza-Arrieta, Maialen; Azkue, Jon Jatsu; Arbillaga-Etxarri, Ane; Lersundi, Ana; Lascurain-Aguirrebena, Ion | 2025 | Exclusion reason: Non-SMT intervention |
| Effects of physical therapy on improving disc height index, postural stability, pain and function in persons with discogenic low back pain. | Osama, Muhammad; Naeem, Aamer; Tariq, Sameen; Mushtaq, Sumera; Siddiqi, Furqan Ahmed; Afridi, Sabah | 2025 | Exclusion reason: Non-SMT intervention |
| Assessment of the Effectiveness of Fascial Manipulation in Patients with Degenerative Disc Disease of the Lumbosacral Spine. | Mikolajczyk-Kociecka, Anna; Kociecki, Marek; Cyrylowski, Lech; Szylinska, Aleksandra; Rynio, Pawel; Gebska, Magdalena; Szuba, Ewelina; Kazmierczak, Jaroslaw | 2024 | Exclusion reason: Non-SMT intervention |
| Dry needling versus instrument assisted soft tissue mobilization in patients with cervicogenic headache: a randomized controlled trial | Elhafez, HM; Mohammed, OA; Fahmy, EM; Elkhateeb, YS; Abd El-Azeim, AS | 2025 | Exclusion reason: Non-SMT intervention |
| Effects of cervico-lumbar combination manual therapy in patients with cervical radiculopathy : a randomized controlled pilot trial | Ziqing, L; He, C; Zhangjie, L; Qiangqiang, Y; Lan, S; Chaoyang, D | 2025 | Exclusion reason: Non-SMT intervention |
| Short-term results of instrument-assisted soft tissue mobilization in female patients with myofascial pain syndrome: a randomized blinded sham-controlled study | Cigdem-Karacay, B; Sahbaz, T; Gumusay, D; Kodak, M-I; Tuncay, F; Ozudogru, A; Kocak, F-A | 2025 | Exclusion reason: Non-SMT intervention |
| Is regression in lumbar disk herniation possible by spinal mobilization? A single-blind randomized controlled clinical study | Taskaya, B; Taskent, I; Cakilli, M; Tunca, O | 2025 | Exclusion reason: Non-SMT intervention |
| An additive effect of instrument-assisted soft tissue mobilization with spinal manipulation in cervicogenic headache: a randomized controlled trial [with consumer summary] | Nambi G; Alghadier M; Khanam H; Pakkir Mohamed SH; Aldhafian OR; Alshahrani NA; Mani P; Chevidikunnan MF; Khan F; Albarakati AJA | 2024 | Exclusion reason: Wrong comparator/same intervention |
| Dry needling and upper cervical spinal manipulation in patients with temporomandibular disorder: a multi-center randomized clinical trial | Dunning J; Butts R; Bliton P; Vathrakokoilis K; Smith G; Lineberger C; Eshleman N; Fernandez-de-Las-Penas C; Young IA | 2024 | Exclusion reason: Wrong patient population |
| [Observation of clinical curative effect of "oblique-pulling" maneuver in the treatment of lumbar intervertebral disc herniation] | Zhang, Jun; Han, Lei; Wang, Peng; Yu, Dong; Lu, Min; Lin, Ding-kun; Song, Tie-bing; Lin, Jiang-hao; Sun, Shu-chun | 2010 | Exclusion reason: Not includable language |
| [Clinical research on the fine tuning manipulation of short lever on spine in treating patients with cervical spondylotic myelopathy] | Chen, J | 2009 | Exclusion reason: Not includable language |
| (Clinical study of effect of rotational manipulation in treating cervical spondylotic radiculopathy using visual analog scales) [Chinese - simplified characters] | Zhu LG, Yu J, Gao JH, Li J, Chang C, Fang J | 2005 | Exclusion reason: Not includable language |
| Clinical effect of lever positioning manipulation for lumbar disc herniation and its influence on Cobb angle | Lyu, LJ; Li, JH; Yang, C; Liu, D; Wang, WW; Mao, LY; Wang, S | 2021 | Exclusion reason: Not includable language |
| Spinal mechanical impulsive manipulation versus conventional physiotherapy for chronic low back pain: differences in range of lumbar motion and trunk muscle strength | Zhongliang, Z; Xiaojie, Y; Bangzhong, L; Min, Z; Liya, Z; Dongdong, Y | 2018 | Exclusion reason: Not includable language |
